# Periodicity of the Affinity of Lanmodulin for Trivalent Lanthanides and Actinides: Structural and Electronic Insights from Quantum Chemical Calculations

**DOI:** 10.1021/acs.inorgchem.3c00754

**Published:** 2023-05-02

**Authors:** Mario Prejanò, Marirosa Toscano, Tiziana Marino

**Affiliations:** Dipartimento di Chimica e Tecnologie Chimiche, Università della Calabria, Via P. Bucci, 87036 Rende, Italy

## Abstract

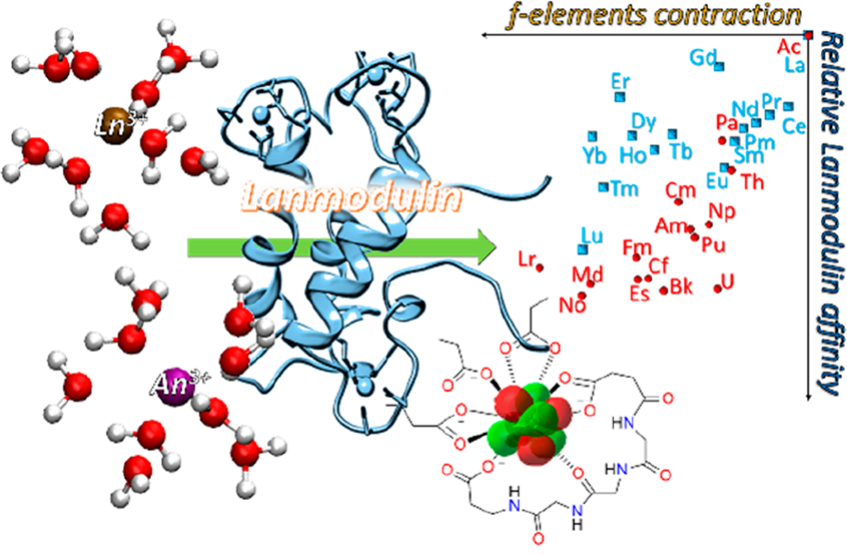

Lanmodulin (LanM) is the first identified macrochelator that has naturally evolved to sequester ions of rare earth elements (REEs) such as Y and all lanthanides, reversibly. This natural protein showed a 10^6^ times better affinity for lanthanide cations than for Ca, which is a naturally abundant and biologically relevant element. Recent experiments have shown that its metal ion binding activity can be further extended to some actinides, like Np, Pu, and Am. For this reason, it was thought that LanM could be adopted for the separation of REE ions and actinides, thus increasing the interest in its potential use for industry-oriented applications. In this work, a systematic study of the affinity of LanM for lanthanides and actinides has been carried out, taking into account all trivalent ions belonging to the 4f (from La to Lu) and 5f (from Ac to Lr) series, starting from their chemistry in solution. On the basis of a recently published nuclear magnetic resonance structure, a model of the LanM-binding site was built and a detailed structural and electronic description of initial aquo– and LanM–metal ion complexes was provided. The obtained binding energies are in agreement with the available experimental data. A possible reason that could explain the origin of the affinity of LanM for these metal ions is also discussed.

## Abstract 2

To provide deeper insights into the interactions of trication species of lanthanides (Ln) and actinides (An) with lanmodulin (LanM), we performed a systematic density functional theory-based investigation on Ln^3+^/An^3+^ aquo- and LanM-bonded complexes. It has been pointed out that the source of LanM binding activity can be related to the 4f and 5f contraction effect and to the contribution of the metal to the composition of the molecular orbitals upon binding the protein.

## Introduction

**Figure 1:**
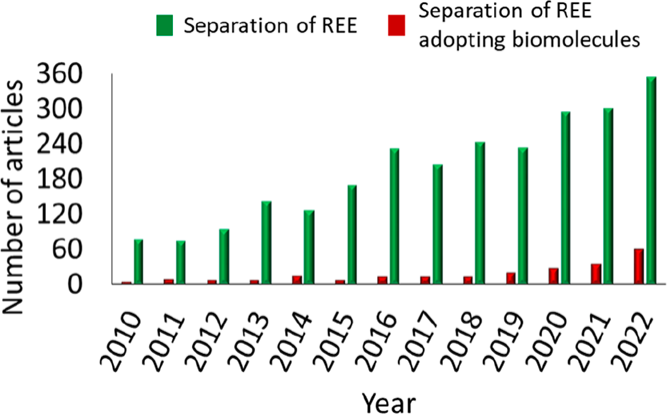
Number of articles regarding the topic of REE separation, in general (green) and mediated by natural molecules (red). The search was conducted on www.scopus.com and updated at March 3, 2023.

Rare earth elements (REEs) are essential metals today that have been widely adopted in a number of industrial applications, and their extraction, separation, and purification are usually performed via chemical and physical methods.^1−6^ With regard to the chemical compounds that can promote the effective separation of REEs, recently, the interest of the scientific community has been directed toward putative systems, mostly biological, that can “interact”, at different levels, with cations of REEs, like Sc, Y, and lanthanides (Ln)^7−9^ (see Figure 1).

Initially, the dearth of naturally occurring macromolecules capable of binding REEs efficiently drove industry and the associated research field to favor small and man-made chelators, thus ignoring proteins for the life cycle of REEs. The later discovery of the acquisition and/or utilization of certain Ln by methylotrophic bacteria then opened to the possibility that natural and efficient macromolecules that bind REEs might exist.^10−13^ It was well-known indeed that, among the macromolecules, a number of proteins could selectively interact with biologically relevant metal ions, usually belonging to the group of alkaline earth metals, like Ca^2+^,^14^ or transition metal series, like cations of Fe and Cu,^15,16^ instead of 4f elements. The reason for such behavior is most likely related to the composition of the protein binding site, which can partially lack enough amino acid residues to accommodate REEs and, more specifically, lanthanides. In addition, the chemistry of f elements significantly differs from that of other blocks of the periodic table, with almost no redox reaction under environmental conditions (except for the Ce^4+^/Ce^3+^ couple),^17^ a coordination number (CN) of 8–10 compared to the usual values of 4–6, and a larger ionic radius of ∼1 Å relative to d-block metals.^18^

Therefore, the uptake of REEs by proteins was mainly studied via metal substitutions within proteins naturally tailored to bind metal ions arising from elements of s- and d-blocks.^19^

Only in 2019, a metal-binding macromolecule that is extremely selective for lanthanide cations was reported, lanmodulin (LanM) that is a small ∼12 kDa protein produced by a certain methylotrophic bacterium, *Methylorubrum extorquens* AM1. It was isolated and characterized as a natural and reversible REE-binding protein that does not need of any co-chelator agent to explicate its activity.^20^ This discovery has opened new horizons in the separation of metal ions, including those of f-block elements.^7,21^

A practical example of such an application is represented by the studies of Cotruvo and co-workers, who explored how bacteria selectively acquire and utilize lanthanides, applying the obtained information in the design of biotechnologies for the detection and capture of REE metal ions.^7,20,21^ The interest in LanM activity has further grown because of the evidence of the extended metal affinity for some actinides (An). It has been experimentally observed that LanM can selectively bind Am and Cm in solution, generating more stable complexes than those obtained with trivalent lanthanide cations having ionic radii similar to those of Nd^3+^ and Sm^3+^.^22,23^

These results evidenced an amazing behavior because for first time LanM, a natural lanthanide-binding protein, was able to discriminate among actinides and lanthanides under certain conditions. It is worth mentioning in this section that, with this finding, it has been proposed “how protein-based biotechnologies may facilitate remediation, detection and potentially separations of lanthanides and heavy actinides”.^22^ Therefore, these outcomes make LanM also a promising actinide-binding protein not only under ideal laboratory conditions but also in more complex, industry-relevant, samples. This represented a further discovery with respect to the LanM’s ability, because the selective extraction of trivalent actinides (An^3+^) over trivalent lanthanides (Ln^3+^) plays a fundamental role in the treatment of nuclear waste, in the partitioning and transmutation strategy,^24^ in the disposal of diagnostic wastes,^25,26^ and in additional fields like sustainable and green chemistry applications, high-tech development, and medical applications.^8^ In this context, leveraging biomolecules for metal extraction technologies became appealing, because these can guarantee quantitative yields, fast kinetics, and high selectivity and fidelity, which are peculiar for most biochemical processes.

Previous studies of LanM’s characterization under physiological conditions (pH 7.2) determined that the metal binding occurs indirectly via a large conformational change in the protein.^10,11^ In addition, structural information arising from nuclear magnetic resonance (NMR) spectroscopy in solution of the structure of Y^3+^-bound LanM has revealed that the LanM’s metal sites share some structural similarities with calmodulin (CaM), the well-known natural Ca^2+^-binding protein.^20^ However, it might bind Ln with a CN of 8 or 9, which is higher than that of typical EF-hand/Ca^2+^ interactions (7) of CaM, depending on the dimensions of the ions and the site.^20,23^

Despite some indications, the mechanism of metal ion recognition by LanM is still not fully clear, and the origin of the pH condition and thermal stability of the LanM–metal complexes is currently unknown. A recent hypothesis pointed out the relationship of the intrinsically disordered nature of the apoprotein that might favor the selectivity for f elements rather than others.^27,28^

Motivated by all of the results mentioned above and by the need for in-depth elucidation, we performed a fully quantum-mechanical study on the behavior of LanM with respect to Ln^3+^ and An^3+^. In detail, driven by the experience gained from previous works on the biorelevance of Ln^3+^,^21,29−35^ and starting from the recently released NMR structure of the LanM–Y^3+^ complex, we chose a representative model of the EF loops of LanM to carry out the investigation. Our aim was to provide, at the atomistic level, deeper insights into the “selective” behavior of protein toward trications of f elements, in terms of electronic and energetic properties. A scheme for calculating relative binding enthalpies was proposed and adopted, considering the energy of geometry-optimized aquo and protein complexes of all trivalent Ln^3+^ (Ln = Ce–Lu) and An^3+^ (An = Th–Lr) ions with respect to the energies of the earliest La^3+^ and Ac^3+^, respectively, and results were compared with the available experimental data. The structural and electronic insights helped to shed light on the origin of the preferences of LanM for f-block elements and can play an important role in improving future applications of this protein system and/or pave the way for the development of LanM-inspired chelators.

## Computational Methods

### Choice of Model

**Figure 2:**
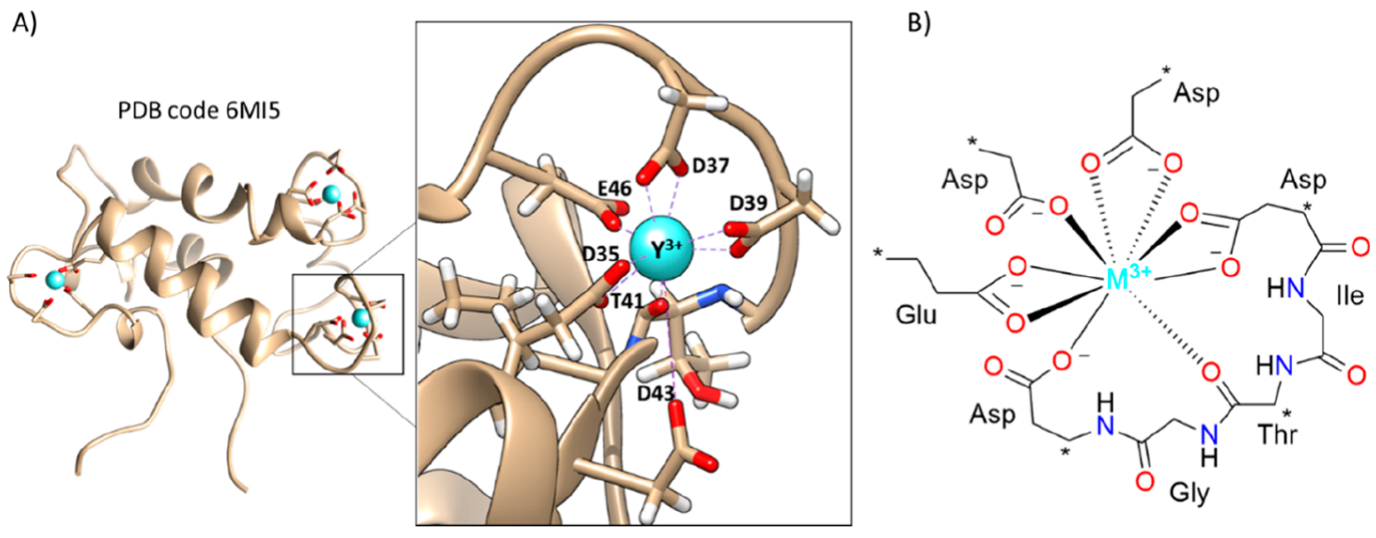
(A) Representation of the LanM–Y^3+^ complex obtained from PDB entry 6MI5.^20^ (B) Schematic representation of the QM cluster model adopted in this work.

In this investigation, 60 complexes (30 aquo and 30 LanM complexes) were optimized to gain structural and electronic information along the series of lanthanides and actinides. To simulate the coordination environment of the ion restricted in the LanM-binding site, a model was built from the solution structure of the LanM–Y^3+^ complex, determined by NMR spectroscopy [Protein Data Bank (PDB) entry 6MI5],^20^ as depicted in Figure 2. An oxidation state of +3 was chosen for both lanthanides and actinides. The trivalent cation corresponds to the dominant form in aqueous solution for Ln^36−39^ and was analogously selected for An in compliance with the limited data in the literature. The chosen model reflects well the oxophilic nature of Ln^3+^ and An^3+^, which are notoriously hard Lewis acids that prefer oxygen donors. In the case of An^3+^, because of the more spatially diffused 5f orbitals, the hard character becomes less evident compared to that of the 4f orbitals of Ln^3+^.

In analogy with the ubiquitous eukaryotic CaM, a Ca^2+^-binding protein,^40−42^ the metal recognition motifs in the LanM are EF hands. Such motifs are ∼29-residue helix–loop–helix motifs that include a 12-residue, carboxylate-rich metal-binding loop flanked by entering and exiting α-helices.^36,43^

LanM possesses four predicted EF loops, EF1 (D35–E46), EF2 (D59–E70), EF3 (D84–E95), and EF4 (N108–E119), which, being at the periphery of the protein surface, are exposed to the solvent and can catch cations. Only three binding sites showed picomolar affinity for Ln, while a fourth is characterized by approximately micromolar affinity, which ensures high selectivity even in the presence of high concentrations of non-Ln cations.^20^

The analysis of the NMR structure revealed that the metal ion is eight- or nine-coordinated, in each EF, and that the ligands are four or five carboxylate side chains and a backbone carbonyl group, corresponding to the conserved carboxylates of D/E and T conserved residues, respectively.^20^ High coordination numbers represent a common aspect observed in Ln^3+^ complexes, such as the high level of carboxylates as ligands because of their hard nature.^44^ To reproduce this behavior, the here-adopted LanM model was selected and thus assumed to be representative of each EF. This model is characterized by eight amino acids, and it can be considered as descriptive of EF1 (D35-D37-D39-G40-T41-I42-D43-E46), EF2 (D59-D61-D63-G64-T65-I66-D67-E70), and EF3 (D84-D86-D88-G89-T90-I91-D92-E95). Amino acids were modeled following the quantum mechanics (QM) cluster approach that has been systematically and successfully adopted in a number of previous investigations aiming to gain atomistic details about different metal-containing systems.^44^

In accordance with this protocol, the residues were truncated at the Cα position and saturated with hydrogens (see Figure 2B). The protonation states of amino acids were assigned in accordance with the available experimental information, i.e., negatively charged carboxylate moieties of E and D residues.^7,22,23^

The C atoms where truncation occurred (labeled with asterisks in Figure 2B) were kept fixed at their initial positions, to avoid artificial movements during the geometry optimizations. This procedure generated a number of imaginary frequencies (by approximately <60*i* cm^–1^) that can be ignored because they do not affect the relative energies of the optimized structures.^45^ In addition, the surrounding protein was modeled as a medium in which the binding site is immersed, as described in Computational Details. The selected models, in summary, consist of 28 atoms (total charge of +3) and 77 atoms (total charge of −2) in the case of aquo and LanM complexes, respectively.

### Computational Details

All of the calculations were carried out using the Gaussian 16 package.^46^ For geometry optimizations, the B3LYP-D3^47−50^ functional was used in conjunction with the 6-31 G(d,p) basis set for the C, N, O, and H atoms. The effective core potential SDD coupled with its related basis set^51^ was selected for metal ions, to ensure quasi-relativistic effects for valence electrons in the treatment of f elements.^29−31^ The same computational procedure was successfully applied in the mechanistic studies of the methanol dehydrogenase (MDH) from *Methylacidiphilum fumariolicum* SolV and other metalloproteins.^29−31,44,52^ The spin multiplicities were chosen following the indications obtained from the systematic analysis of the lanthanide-containing MDH systems, based on unrestricted Kohn–Sham (UKS) calculations.^30^ For the sake of similarity and in agreement with the available data,^53,54^ the same spin states were considered in the presence of actinides. The selected spin multiplicities can be thus summarized as follows: for Ln^3+^- and An^3+^-containing systems, 2*S* + 1 = 1 for La^3+^/Lu^3+^ and Ac^3+^/Lr^3+^, 2*S* + 1 = 2 for Ce^3+^/Yb^3+^ and Th^3+^/No^3+^, 2*S* + 1 = 3 for Pr^3+^/Tm^3+^ and Pa^3+^/Md^3+^, 2*S* + 1 = 4 for Nd^3+^/Er^3+^ and U^3+^/Fm^3+^, 2*S* + 1 = 5 for Sm^3+^/Ho^3+^ and Np^3+^/Es^3+^, 2*S* + 1 = 6 for Sm^+^/Dy^3+^ and Pu^3+^/Cf^3+^, 2*S* + 1 = 7 for Eu^3+^/Tb^3+^ and Am^3+^/Bk^3+^, and 2*S* + 1 = 8 for Gd^3+^ and Cm^3+^, respectively. A summary of the adopted multiplicities is presented in Table S1. Spin contamination was carefully checked by monitoring the expected value of the total spin, ⟨*S*^2^⟩, and the related results are listed in Tables S1 and S2. No significant spin contamination can be observed (<7%). All of the structures were optimized without imposing any symmetry constraints. Optimized geometries were verified to be true minima by vibrational frequency analysis at the same level of theory. Single-point calculations taking into account solvation effects were carried out with the SMD continuum solvation model on every optimized geometry of both Ln^3+^ and An^3+^ aquo complexes and of their corresponding protein models, choosing a dielectric constant (ε) of 4 to simulate the protein environment, as previously demonstrated,^29,30,55^ while water solvent was considered to calculate the solvation effects for the aquo complexes.

More accurate electronic energies were obtained by single-point energy calculations on the optimized structures, selecting the 6-311+G(2d,2p) larger basis set for all atoms, except for f elements. The final energies include, in addition, the zero-point energy corrections and solvation effect. The affinities of LanM for lanthanides and actinides ions were calculated as relative binding enthalpies with respect to La^3+^ and Ac^3+^, considering a process indicating the tendency of each lanthanide/actinide to replace the solvent molecules from its first coordination shell with a carboxylate group of the protein, in accordance with the following reaction:
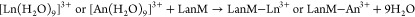
The choice to calculate relative binding enthalpies (Δ*H)* did therefore not need the inclusion of LanM and H_2_O energies, being calculated as the energetic difference as follows:
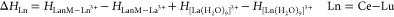

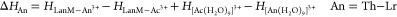
Further details are provided in the Supporting Information. In the expressions presented above, the entropic contributions to the relative binding energies were not further calculated and were assumed to be equal to that of La^3+^ or Ac^3+^ for each lanthanide or actinide, respectively. For all complexes of Ln^3+^ and An^3+^, natural bond orbital (NBO) analysis was performed.

## Results and Discussion

### [Ln(H_2_O)_9_]^3+^ and [An(H_2_O)_9_]^3+^ Complexes

**Figure 3:**
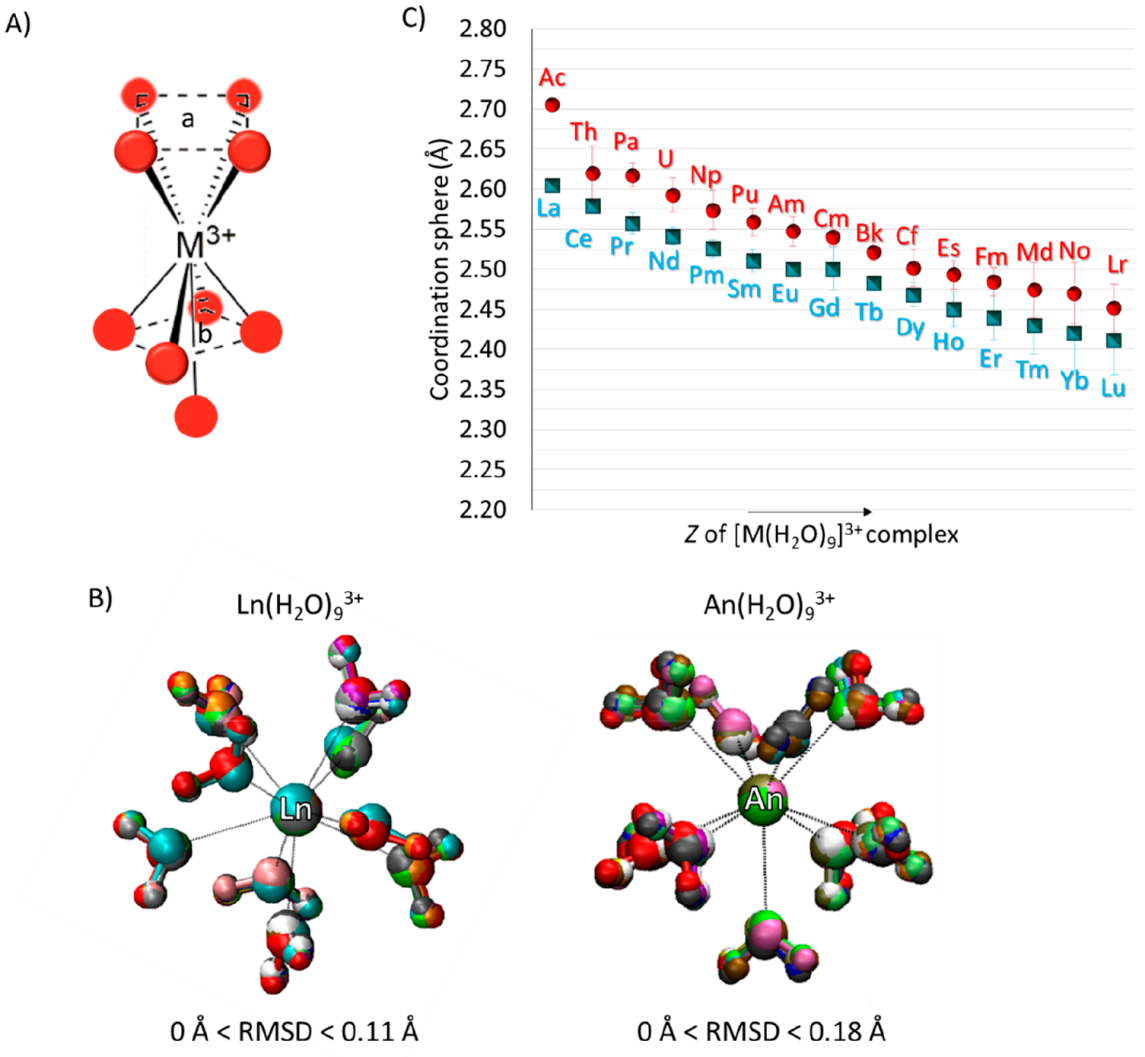
(A) Schematic representation of the geometry obtained for all aquo complexes and (B) superposition of Ln^3+^- and An^3+^-containing systems (each individual optimized structure is shown in Figures S1 and S2, respectively). (C) Average coordination spheres, with relative standard deviations, obtained from the optimized geometries of [Ln(H_2_O)_9_]^3+^ and [An(H_2_O)_9_]^3+^.

For the optimization of the aquo complexes of Ln^3+^ (Ln = La–Lu) and An^3+^ (An = Ac–Lr) species, the initial CN of 9 was set. It is indeed known that the lanthanide’s CN is 8 or 9, while for the actinides, it has been hypothesized, and generally accepted, that the CN is 9 or 10.^56^ It is also suggested that a progressive decrease in the CN, generated by the loss of one water molecule by the metal, along the series can occur from the left to the right of the sixth and seventh periods.^56,57^ No symmetry restraints were imposed during optimization, affording the final structures reported in Figure 3. In the resulting geometries, all of the water molecules tended to maintain the interaction with the metal center, in a capped square antiprismatic-like fashion, which is characterized by a nine-coordinated metal (see Figure 3A). Further optimizations were attempted on 10-coordinated actinides, but the results were not fruitful because a nine-coordinated metal center was again obtained. For this purpose, the study was conducted by considering nine-coordinated geometries for both Ln^3+^ and An^3+^ species.

**Figure 4:**
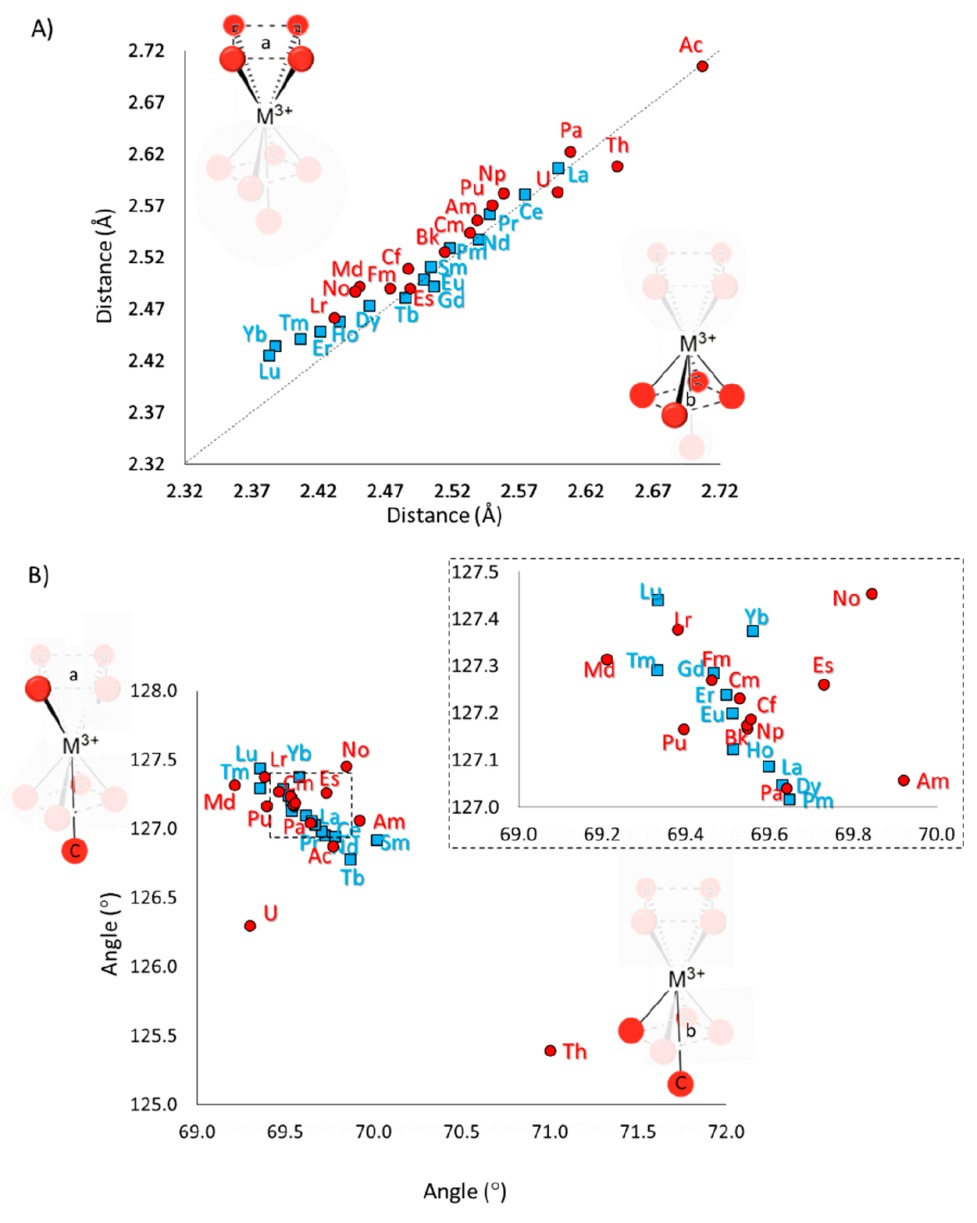
Main (A) bond distances and (B) bond angles obtained from the optimizations of [Ln(H_2_O)_9_]^3+^ and [An(H_2_O)_9_]^3+^ complexes.

The optimized structures present very similar geometries, as can be evinced by the calculated root-mean-square deviations (RMSDs) of 0.11 and 0.18 Å, in the case of superimposed lanthanide and actinide structures, respectively (see Figure 3B and Figures S1 and S2 for each optimized structure). Proceeding through the series, we observed a contraction of the coordination sphere of the metal center, for both lanthanides and actinides. In particular, in the case of the [Ln(H_2_O)_9_]^3+^ series, the average distance from water molecules to the metal varies from 2.60 Å (Ln^3+^ = La^3+^) to 2.41 Å (Ln^3+^ = Lu^3+^), corresponding to a decrease of 7% (see Figure 4A). This result is in agreement with the so-called “lanthanide contraction effect”, the physical phenomenon that characterizes the 4f elements and a more pronounced progressive decrease in ionic radius along the period, with respect to s-, p-, and d-block elements.^57,58^

The explanation of such behavior lies in the minor shielding effect from electrons occupying 4f orbitals of the positively charged nuclei. In the case of the actinides, the variation of the coordination sphere has been observed in the range of 2.71–2.45 Å, from Ac^3+^ to Lr^3+^, which corresponds in turn to a 10% decrease in the distances from the metals (see Figure 3C).

A more detailed analysis of the distances of water molecules from the metals revealed that they do not lie at the same distance from the cations despite very similar structural arrangements (see Figure 4A). In detail, the analysis of correlation graphs between the O–M^3+^ bonds of ligands lying on *a* and *b* planes points out that, for Ln^3+^ = La^3+^–Tb^3+^ very similar O_*a*_–M^3+^ and O_*b*_–M^3+^ distances can be observed (see Figure 4A). The Gd^3+^ aquo complex represents an exception, as indicated by the values of 2.49 and 2–51 Å for the O_*a*_–Gd^3+^ and O_*b*_–Gd^3+^ distances, respectively. In the case of Dy^3+^–Lu^3+^, ligands on *b* tend to move closer to the metal (2.47–2.37 Å O_*b*_–M^3+^ bond vs 2.47–2.42 Å O_*a*_–M^3+^ bond), most likely due to the clashing with the ninth water molecules positioned on the capped face [position C (see Figure 4B)] of the plan that dictates a slightly different displacement of waters.

In analogy to the related 4f elements, also An^3+^ aquo complexes showed a reduction of the metal cation coordination sphere, in agreement with the “actinide contraction effect”. However, the trend obtained for the actinides shows more deviations from ideal symmetry with respect to the lanthanides. Indeed, in addition to the late actinides (An^3+^ = Fm^3+^–Lr^3+^), Pa^3+^ and Np^3+^–Cf^3+^ are characterized by O_*b*_–M^3+^ distances that are shorter than O_*a*_–M^3+^ distances, while in the case Th^3+^ and U^3+^, the opposite has been observed [2.64 Å O_*a*_–Th^3+^ bond vs 2.61 Å O_*b*_–Th^3+^ bond and 2.60 Å O_*a*_–U^3+^ bond vs 2.58 Å O_*b*_–U^3+^ bond, respectively (see Figure 4A)].

The analyses of the bond angles were carried out by calculating the angles between ligands on the *a* or *b* plane with respect to O_C_, i.e., the water molecules on the capped face of the complex [O_*a*_M̂^3+^O_C_ and O_*b*_M̂^3+^O_C_ (see Figure 4B)]. The majority of the [M(H_2_O)_9_]^3+^ complexes presented a very similar distribution of angle values, ranging from 127.0° to 127.5° and from 69.0° to 70.0° in the case of O_*a*_M̂^3+^O_C_ and O_*b*_M̂^3+^O_C_, respectively. As highlighted previously, Th^3+^ and U^3+^ slightly deviate from the general trend, being characterized by O_*a*_M̂^3+^O_C_–O_*b*_M̂^3+^O_C_ pair bond angles of 125.4–71.0° and 126.3–69.3°, respectively. It was interesting to observe that, despite a reduction of the coordination sphere and the ionic radius, the aquo complexes of both 4f and 5f species maintained the bonding to all of the water molecules explicitly included in the model (see Figure S3). Such behavior will not be observed in the case of LanM–Ln^3+^ and LanM–An^3+^ complexes, as will be discussed below, because a reduction in the CN from 9 to 8 was found for late 4f and 5f metals.

**Figure 5:**
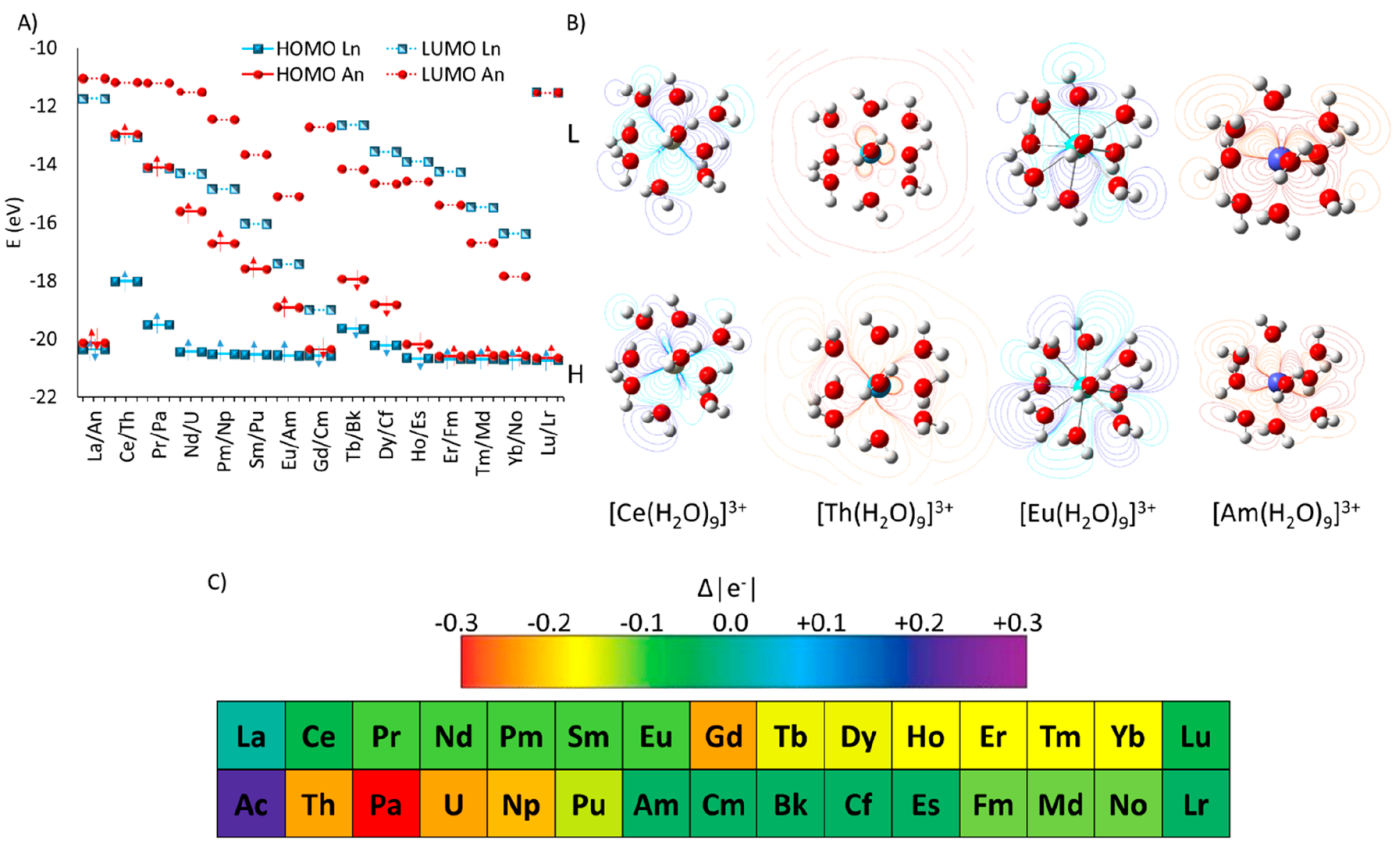
(A) HOMO–LUMO orbitals of all Ln^3+^ and An^3+^ aquo complexes. (B) Calculated counter plots of the HOMO–LUMO of [Ce(H_2_O)_9_]^3+^, [Th(H_2_O)_9_]^3+^, [Eu(H_2_O)_9_]^3+^, and [Am(H_2_O)_9_]^3+^. (C) Relative NBO charges of all trications (all values are listed in Table S3).

Despite the slightly different geometrical parameters discussed above, some interesting differences were observed from the comparison of frontier molecular orbitals (MOs) of 4f- and 5f-containing systems (see Figure 5A). For most of the lanthanides, the contribution to the composition of the highest occupied molecular orbitals (HOMOs) is mainly due to the O atom of water molecules, while the 4f electrons contribute importantly to the lowest unoccupied molecular orbitals (LUMOs). Except for Ce^3+^, such behavior is particularly evident for the first half of lanthanides with respect to the actinides, which, in turn, displayed electrons on HOMOs having 5f character, and lying at higher energies as reported in Figure 5B.

Additional and detailed data for the MOs are collected in the Supporting Information (see Figure S4) as well as the spin densities, which were localized, in all of the considered cases, on the metal center (see Figure S5). The f character on the first virtual orbital, however, favors the stabilization of the LUMOs, for which a decreasing energy trend has been observed among the 4f and 5f series. This energetic behavior is a direct consequence of the lanthanide/actinide contraction effect, for which an increasing Lewis acidity (more stable LUMO) and charge density are usually observed. In particular, the effect of charge density can be observed by analyzing the relative NBO charges, because all Ln^3+^ = Ce^3+^–Yb^3+^ and An^3+^ = Th^3+^–No^3+^ species showed a more negative charge density with respect to La^3+^ (see Figure 5C). Analysis of the spin population revealed that, as expected, the unpaired electrons mainly lie on Ln^3+^ and An^3+^, with spin density maps resembling the shape of 4f and 5f atomic orbitals (see Figure S5).

These observations support, finally, the formation of complexes with LanM, which will be discussed in detail in the next section.

### LanM–Ln^3+^ and LanM–An^3+^ Complexes

The study of LanM’s affinity for Ln^3+^ and An^3+^ started with testing the reproducibility of the chosen NMR structure of the protein-binding Y^3+^ species by the adopted level of theory.^20^ As can be evinced by the comparison of available bond distances, the final obtained structure results shifted an average of 8% from the experimental one after optimization, with an RMSD value of 0.69 Å (see Figure S6 and Table S4).^20^ This small shift was mainly caused by the occurrence of a hydrogen bond interaction between an oxygen of the carboxylate moiety of D37 and the backbone N of T41, which was not observed in the NMR structure (see Figure S6). Overall, the reproduction of the experimental structural data was considered satisfactory, and the model was reputed to be good enough to keep with the investigation.

**Figure 6:**
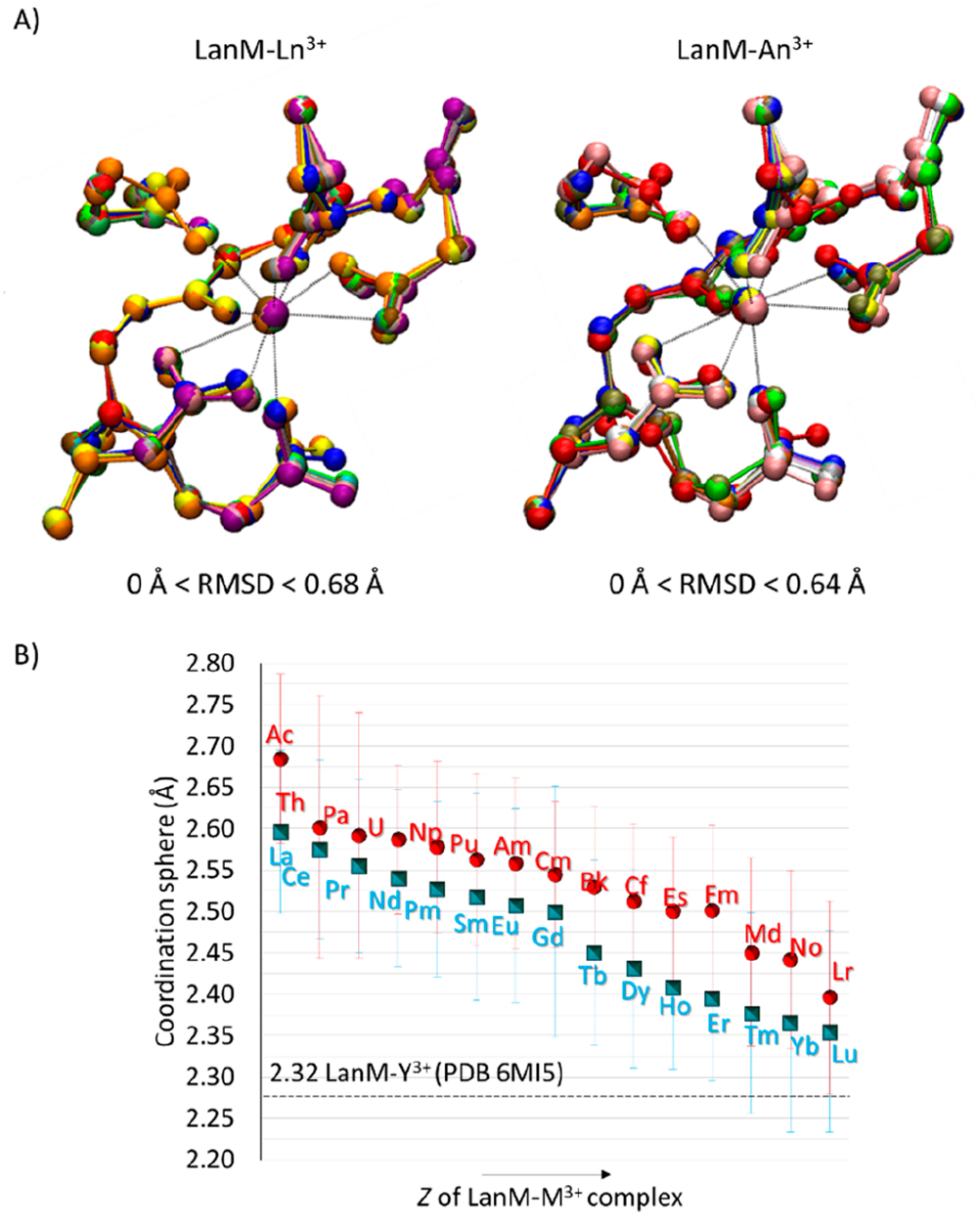
(A) Superimposition of all LanM–Ln^3+^ and LanM–An^3+^ optimized structures (each optimized structure is shown in Figures S7 and S8, respectively). (B) Relative average coordination sphere of the metal, including the standard deviation. For the sake of clarity, hydrogens were not included in the representation. Values of >2.80 Å were not included in the calculation of the average coordination sphere.

After this test, the investigation proceeded with the systematic substitution of Y^3+^ with each Ln^3+^ and An^3+^ and geometry optimization. The structures, depicted in Figure 6A, presented homogeneous geometrical displacements, having RMSD values of superimposed LanM–Ln^3+^ and LanM–An^3+^ complexes in the range of 0.68 and 0.64 Å, respectively (see individual optimized structures in Figures S7 and S8). As in the case of the aquo complexes, the metal’s coordination spheres decreased in size along the series of both Ln^3+^ and An^3+^ species, due to the contraction effect generated by 4f and 5f electrons, respectively. Such a variation was slightly larger in the case of the protein complex. This is evidenced by decreasing values of the average coordination sphere of the metal from 2.60 Å (La^3+^) to 2.35 Å (Lu^3+^) for lanthanides {9% vs 7% of [Ln(H_2_O)_9_]^3+^} and from 2.68 Å (Ac^3+^) to 2.40 Å (Lr^3+^) for actinides {11% vs 10% of [An(H_2_O)_9_]^3+^}.

On the contrary for the relative aquo complexes, a reduction in the CN from 9 to 8 was observed, for both lanthanides and actinides. In particular, this occurred from Ho^3+^ to Lu^3+^ lanthanides and for Md^3+^, No^3+^, and Lr^3+^ actinides, at the expense of one bidentate carboxylate group (see Figure S9). Additional attempts to determine the stability of the actinide coordination sphere were made by testing the possibility of 10 coordination by including in the model one additional explicit water molecule. In analogy to the aquo complexes, 10-coordinated LanM–An^3+^ complexes were not stable due to the loss of one ligand during geometry optimization.

**Figure 7:**
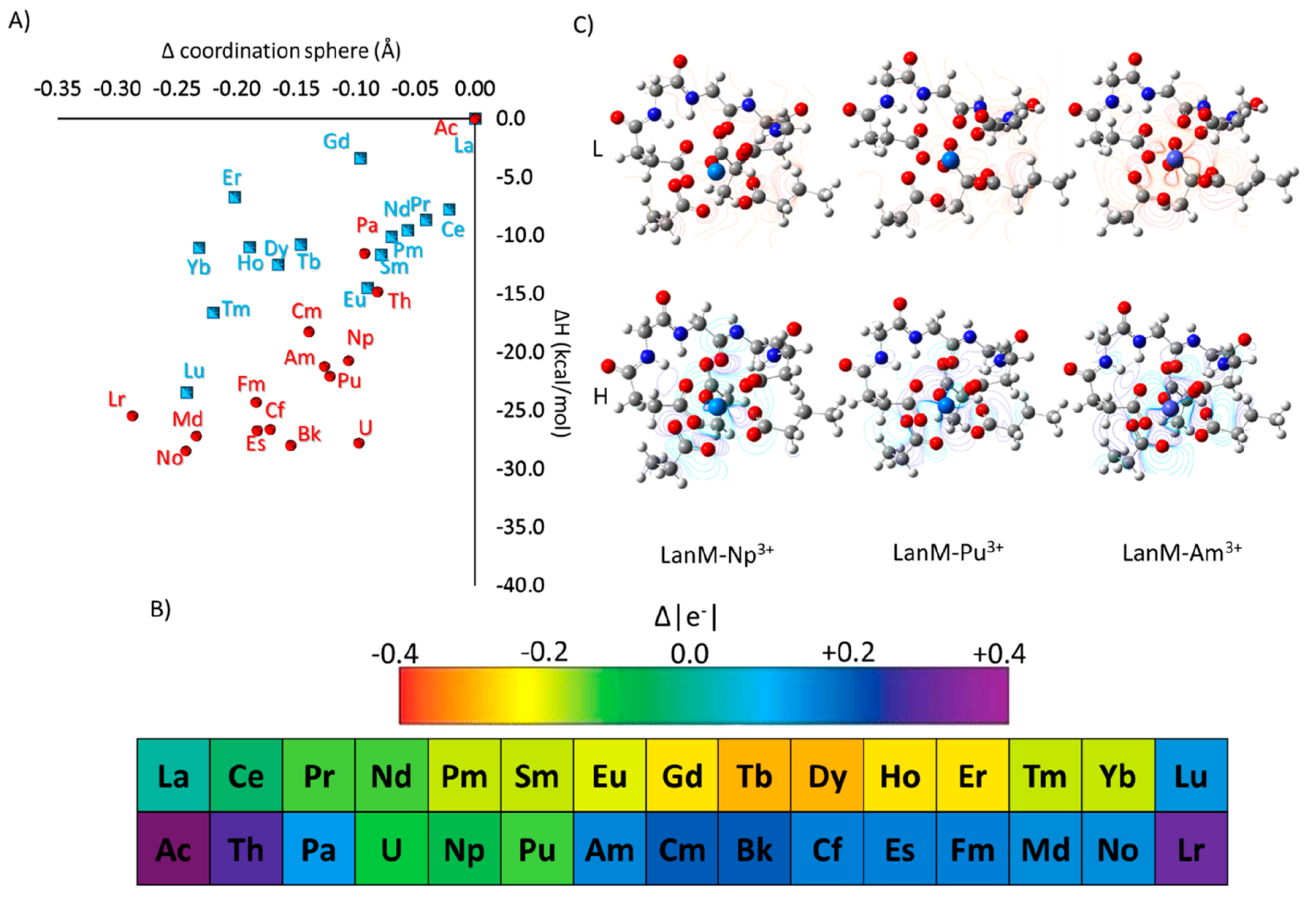
(A) Δ*H*–Δ coordination sphere correlation graph obtained for the LanM–Ln^3+^ and LanM–An^3+^ complexes, relative to the LanM–La^3+^ and LanM–Ac^3+^ complexes, respectively. (B) Relative NBO charges of all trivalent species in the LanM–Ln^3+^ and LanM–An^3+^ complexes (all values are listed in Table S3). (C) HOMO–LUMO representations calculated for the LanM–Np^3+^, LanM–Pu^3+^, and LanM–Am^3+^ complexes.

The calculation of the relative binding affinities for Ln^3+^ revealed a dependency on the contraction of the coordination sphere, as one can see in Figure 7A, in agreement with the experimental measurement of LanM’s time constant exchange of the 4f elements.^23^ Indeed, in general, in the series, negative values of binding affinities were calculated with respect to the La^3+^ species, until reaching the lowest value of −23.4 kcal/mol, obtained for Lu^3+^.

This trend confirms that the dimension of the cations in solution can control LanM’s chelating activity.

A good linear trend for early lanthanides (Ln^3+^ = Ce^3+^–Pm^3+^) and some middle lanthanides, like Sm^3+^ and Eu^3^, has been observed, while deviations for middle (Ln^3+^ = Eu^3+^–Dy^3+^) and some late 4f series elements (Ln = Ho^3+^ and Er^3+^) have been obtained. These deviations are mainly related to some oscillation of the calculated binding enthalpies calculated for Tb^3+^, Dy^3+^, Ho^3+^, and Er^3+^, corresponding to −10.8, −12.5, −11.0, and −6.7 kcal/mol, respectively. A possible explanation of such behavior can be found in the lack of a perfect adaptation by the model to the variation of the CN from 9 to 8, which has been observed to occur for Tb^3+^–Lu^3+^.

The calculations showed that, also in the case of 5f elements, the LanM’s metal affinity increases along the period as the dimension of the cations decreases, with respect to the LanM–Ac^3+^ complex. A good linear correlation has been obtained for some early actinides, like Pa^3+^ and Np^3+^, and some middle ones (Pu and Am), while for the others, some oscillations were observed. For instance, the calculated metal affinity for No^3+^ (−28.5 kcal/mol) was better than that for Lu^3+^ (−25.5 kcal/mol) (Figure 7A), as opposed to the relative lanthanides Yb^3+^–Lu^3+^ (−11.1 kcal/mol vs −23.4 kcal/mol) (see Figure 7A). A possible explanation of such a difference can be also found in the variation of the CN from 9 to 8 and in the partial adaptation of the model, which takes places for both No^3+^ and Lu^3+^.

Interestingly, for Am^3+^ a relative metal binding affinity of −21.2 kcal/mol has been calculated, which is 11.6 kcal/mol better than that of Nd^3+^, in very good agreement with relative affinities recently discussed for LanM.^22^

The protein prefers the binding with Am^3+^ rather than Np^3+^ and can compete with Pu^3+^, for which metal binding affinities of −20.7 and −22.1 kcal/mol were calculated. In addition to the ionic radius of Am^3+^, discussed previously,^23^ the reason for its behavior could lie in the electronic properties of the LanM–Am^3+^ complex.

Once the protein is bound, Am^3+^ displaces a more positive charge than Nd^3+^, Np^3+^, and Pu^3+^, which can generate a more effective ionic interaction with the negatively charged carboxylate groups present in the model, thus stabilizing the formation of the complex (see Figure 7B). Furthermore, in the case of Am^3+^, frontier orbitals become more stable with respect to those of Np^3+^ and Pu^3+^. For the HOMO, a similar composition has been observed, i.e., mainly on the 5f atomic orbital, LUMOs of Np^3+^ and Pu^3+^ are mainly localized on the carboxylate groups of the binding amino acids (see Figure 7C). The HOMO and LUMO of Am^3+^ indeed result in stronger stabilization, due to the f contribution to the composition of the orbitals (see Figure S10). Finally, in analogy to aquo complexes mentioned above, the LanM–Ln^3+^ and LanM–An^3+^ spin density was mainly localized on the metal center (see Figure S11).

As final comment, it is worth noting that despite the calculated An^3+^ trend that can be considered satisfactory, the possibility that for 5f elements other stable oxidation numbers (>3) have to be considered for calculations of accurate binding affinities cannot be excluded.^56,59^ In general, however, this result shows that the affinity of LanM for lanthanides and actinides has to be “handled” with care and that is difficult to accurately, and a priori, predict and/or reproduce the periodic trend, with particular attention being paid to 5f elements. Further investigations in this direction will be carried out in the future.

## Conclusions

In this study, a systematic investigation of the metal ion affinity of a natural Ln binder, lanmodulin, for lanthanide and actinide trivalent cations was carried out. This protein has been identified as a promising biological macrochelator that can discriminate f-block elements from other metals of the periodic table, opening a door to new technologic development of f element-based and f element-oriented applications.

The geometry of nine-coordinated aquo complexes and of LanM with Ln^3+^ (La^3+^–Lu^3+^) and An^3+^ (Ac^3+^–Lr^3+^) ions was optimized starting from the very recent NMR structure of the LanM–Y^3+^ complex in solution, using a model of the lanmdoulin whose binding site was built up to include all relevant residues involved in the interaction with metals.

The analysis of optimized aquo complexes revealed the capped square antiprismatic organization of water molecules around the metal center, which displayed nine coordination in all of the considered cases. The M^3+^–O_w_ distance is on average reduced throughout the period for both Ln^3+^ and An^3+^ series, in accordance with the lanthanide and actinide contraction effects. Some deviations from the ideal geometry were observed in the case of late Ln^3+^ (Ho^3+^–Lu^3+^) and An^3+^ (Cf^3+^–Lr^3+^) aquo complexes, due to the presence of the ninth water molecule on the capped face. Analysis of the HOMO–LUMO composition revealed differences in the composition of these molecular orbitals. A major f character can be observed in both HOMO–LUMO types of the complexes of actinides, with respect to those of lanthanides.

The model built on the basis of the experimental geometry of the LanM–Y^3+^ complex proved to be appropriate for reproducing the periodicity of the affinity of lanmodulin for Ln and An.

In analogy with the aquo complexes, the series of LanM–Ln^3+^ and LanM–An^3+^ complexes were characterized by a contraction of the metal coordination sphere, while a reduction in the CN from 9 to 8 was observed for late lanthanides (Ho^3+^–Lu^3+^) and actinides (Md^3+^–Lr^3+^). To estimate the relative binding affinity (Δ*H*) of the protein for the metal ions, a protocol that considers the energy of aquo and LanM complexes with respect to that of La^3+^ and Ac^3+^ was proposed and adopted.

Interestingly, a linear correlation of the coordination sphere variation and the increasing affinity of the protein for many Ln^3+^ and An^3+^ species was observed. In line with the experimental observation, indeed, it was confirmed that the affinity of LanM increases with a decrease in the Ln^3+^ coordination sphere. The better affinity of the protein for Am^3+^, with respect to earlier actinides or lanthanides, such as Np^3+^ and Nd^3+^, was rationalized in terms of the HOMO–LUMO composition and more positive density charge of the metal ion.

In summary, the calculated periodicity of the affinity of LanM for Ln and An trications was satisfactory, and our analysis shed light on possible origins of the power of binding of LanM to f elements, starting from a comparison with experimental evidence and expanding theoretically the knowledge of the f element properties to hazardous and radioactive species. The authors hope that the results presented here can stimulate further experiments and in-depth analysis in the field.

## Supporting Information Available

Additional results discussed in the text, absolute energies of all species considered in the investigation, and Cartesian coordinates of optimized geometries (PDF)
