# Supplementary material for: Periodicity of the Affinity of Lanmodulin for Trivalent Lanthanides and Actinides: Structural and Electronic Insights from Quantum Chemical Calculations
Source: Inorg Chem. 2023 May 2;62(19):7461–70. doi: 10.1021/acs.inorgchem.3c00754 (PMC10189734; doi:10.1021/acs.inorgchem.3c00754)
Supplement: Supplementary file 1 — ic3c00754_si_001.pdf [file ic3c00754_si_001.pdf]

-Supporting information-

Periodicity of Lanmodulin Affinity towards Trivalent Lanthanides and Actinides: Structural and Electronic Insights from Quantum Chemical Calculations

Mario Prejanò,* Marirosa Toscano and Tiziana Marino*

Correspondence should be addressed to: tiziana.marino65@unical.it ; mario.prejano@unical.it

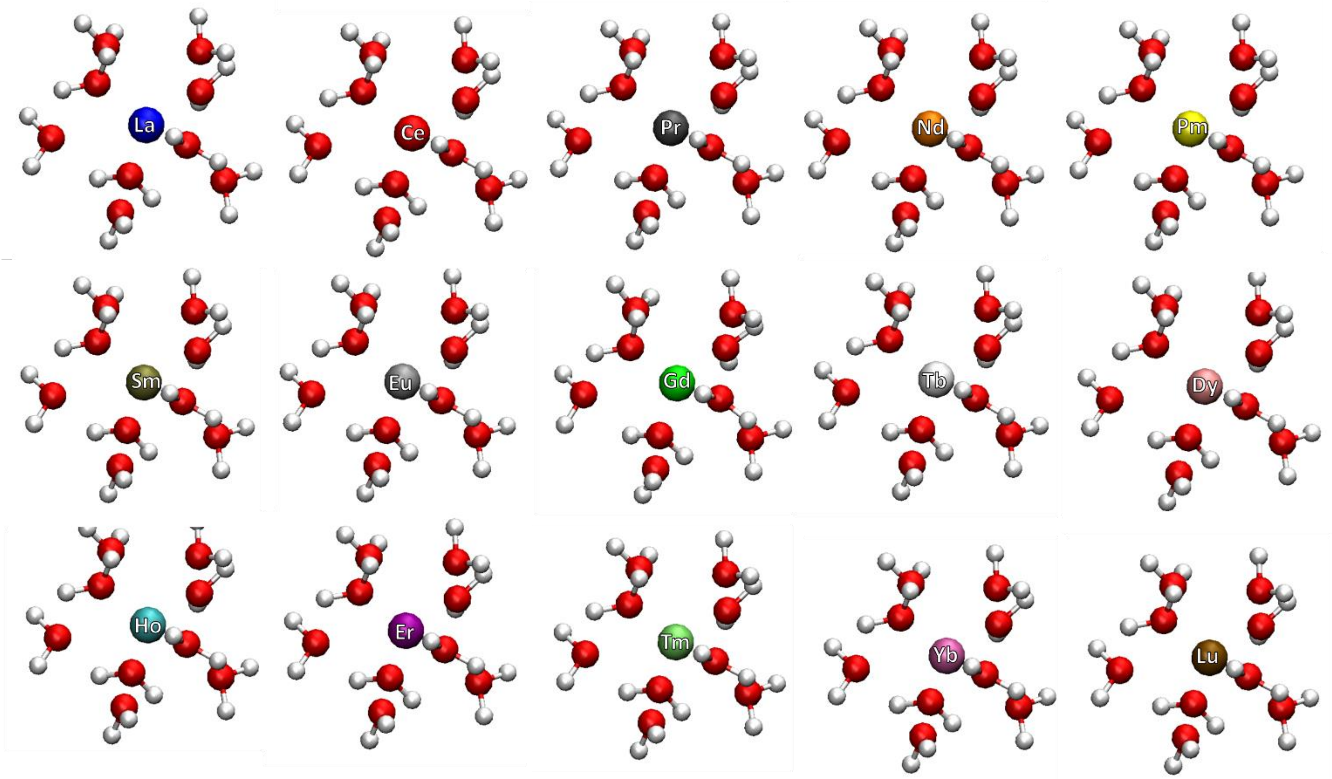

Figure S1. Optimized structures of Ln^{3+}- aquo-complexes.

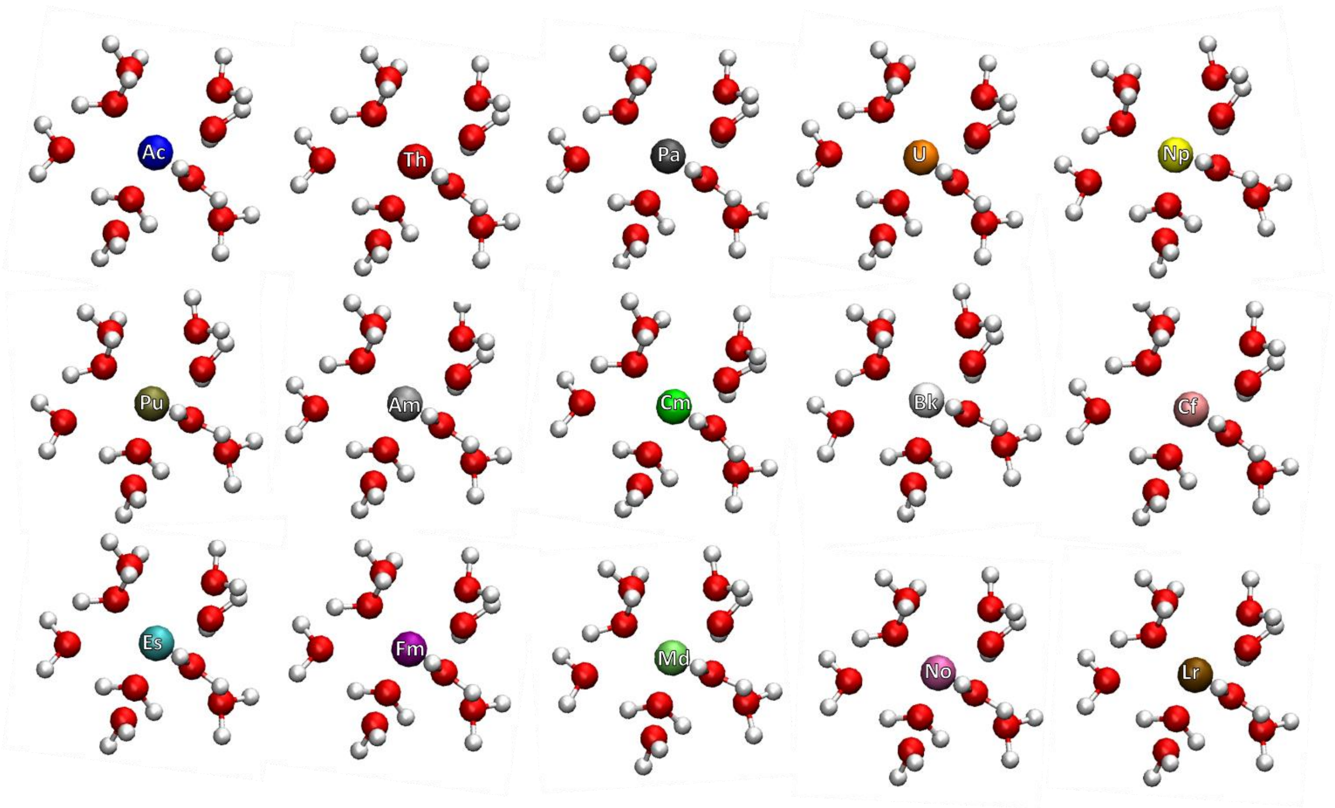

Figure S2. Optimized structures of An^{3+}- aquo-complexes.

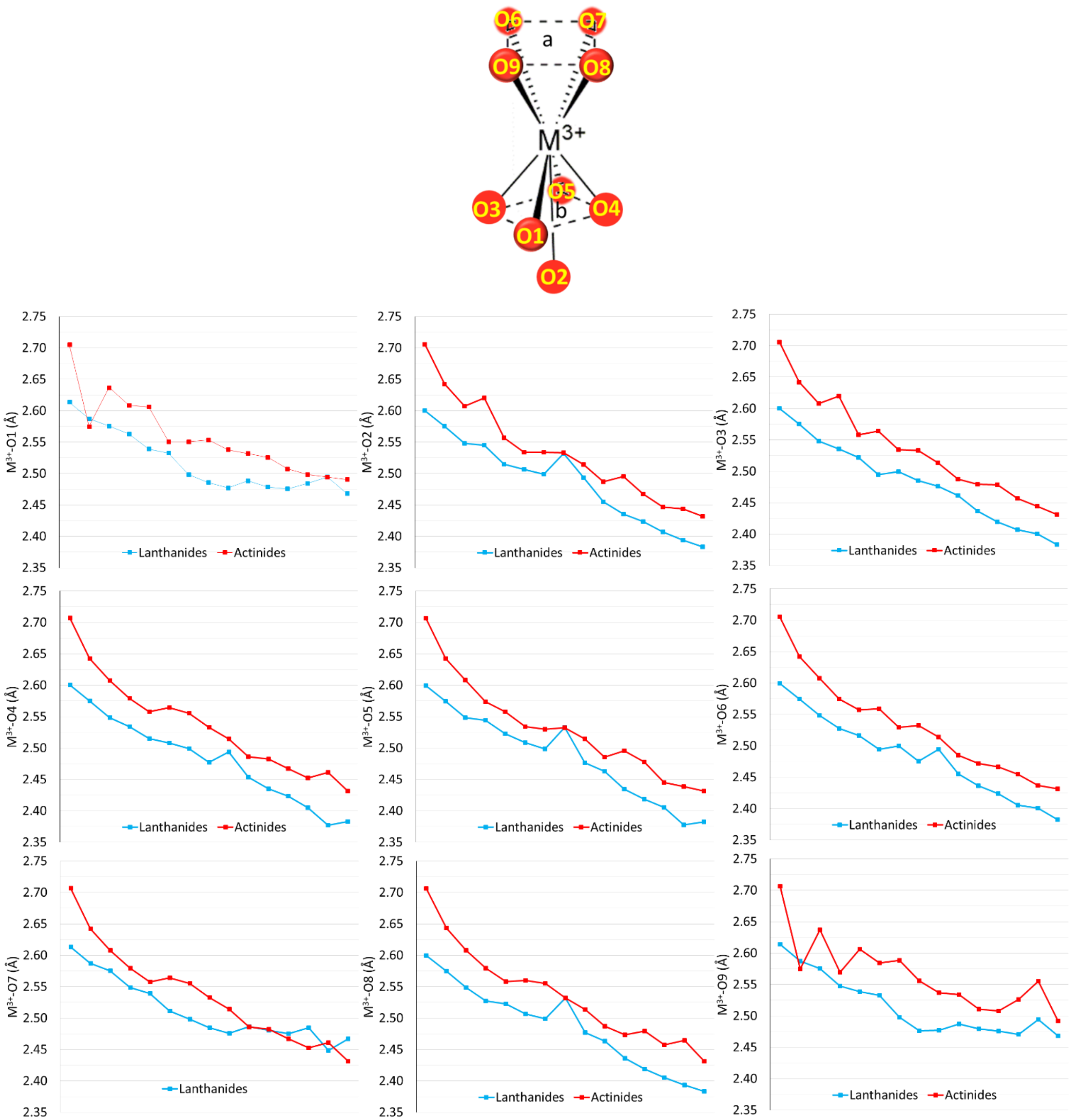

Figure S3. M^{3+}-O_w distances obtained from geometry optimization of Ln^{3+}-and An^{3+}-aquo-complexes.

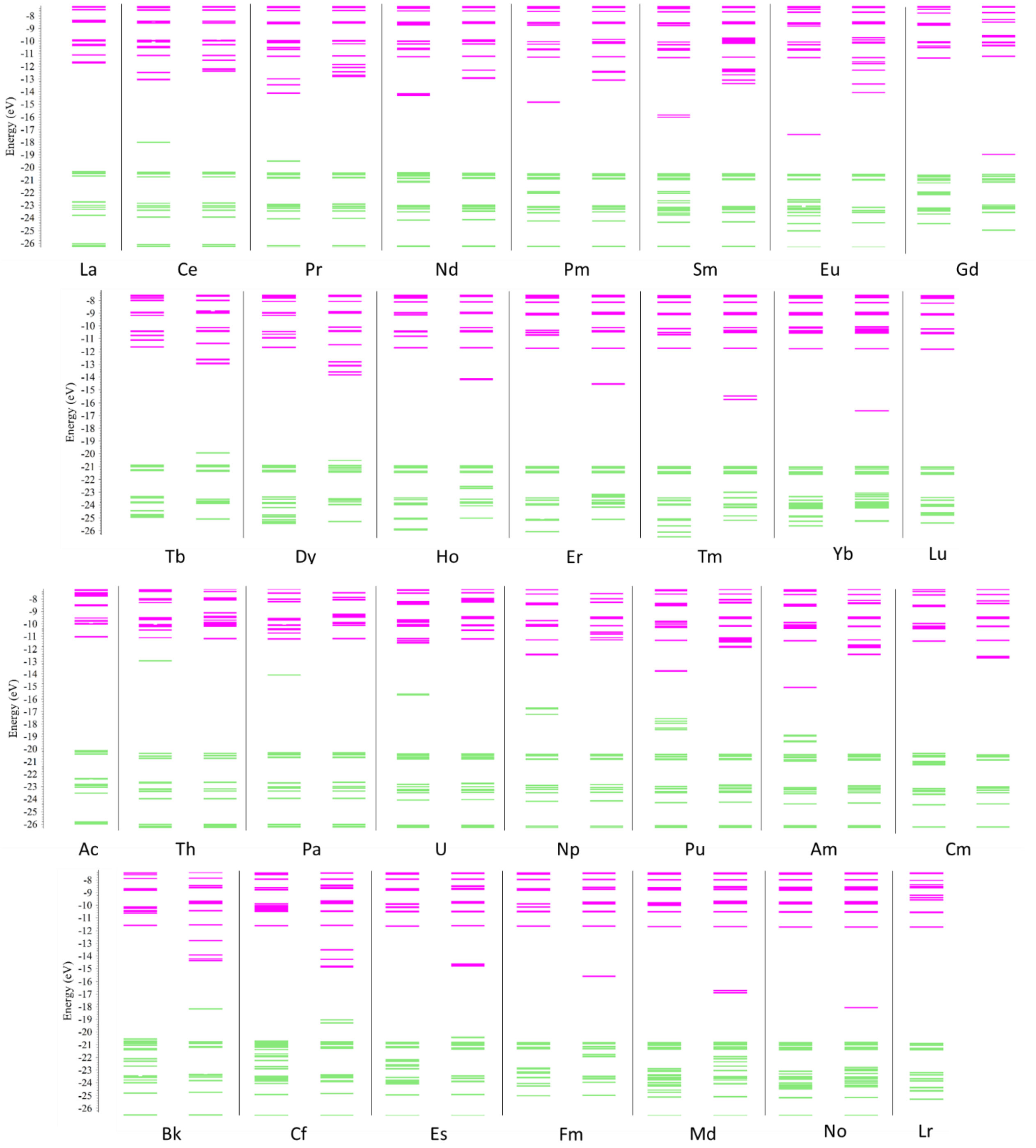

Figure S4. Molecular orbitals calculated for Ln^{3+}-and An^{3+}- aquo-complexes.

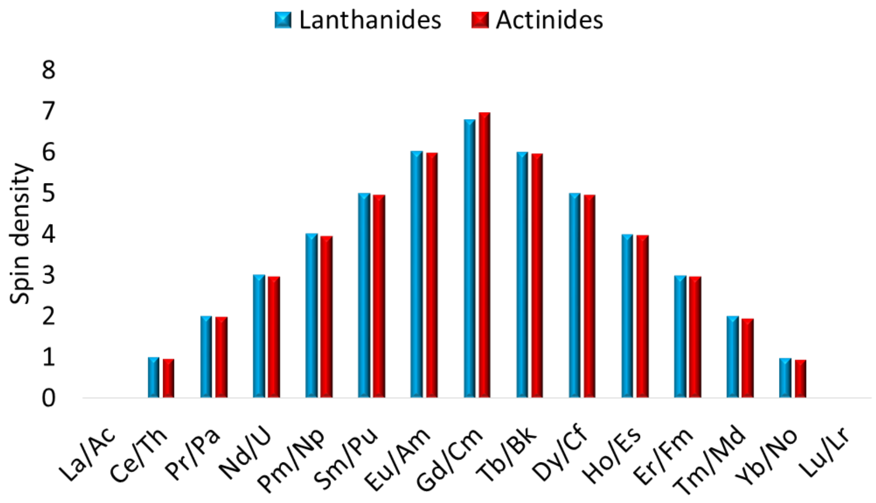

Figure S5. Calculated NBO spin density of metal ions, obtained for Ln^{3+}-and An^{3+}- aquo-complexes.

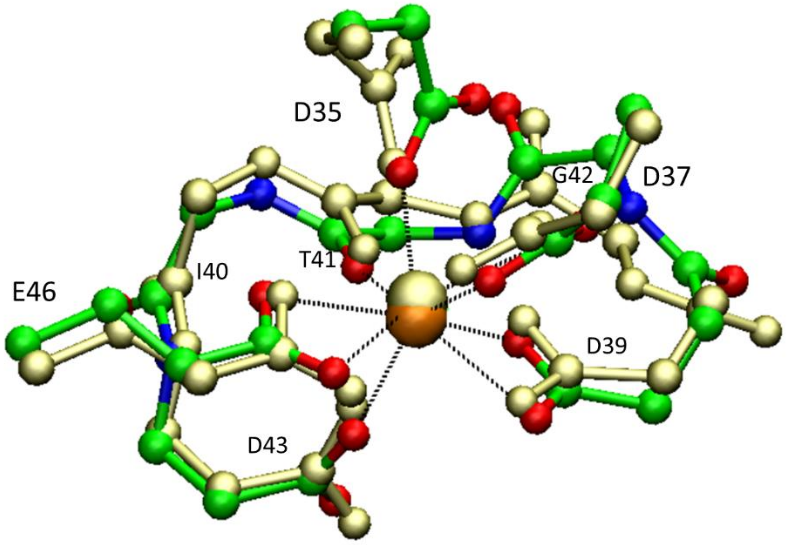

Figure S6. Superposition of NMR (PDB 6IM5, in yellow) and optimized geometry of LanM-Y^{3+}structures.

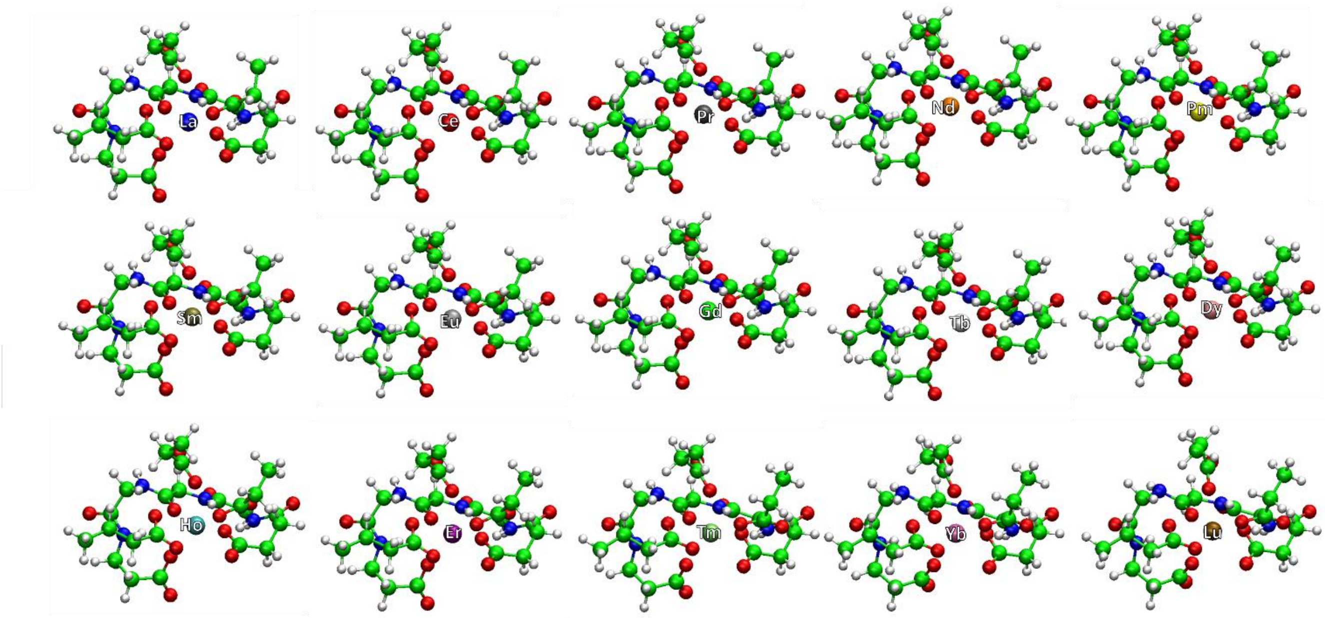

Figure S7. Optimized structures of Ln^{3+}- aquo-complexes.

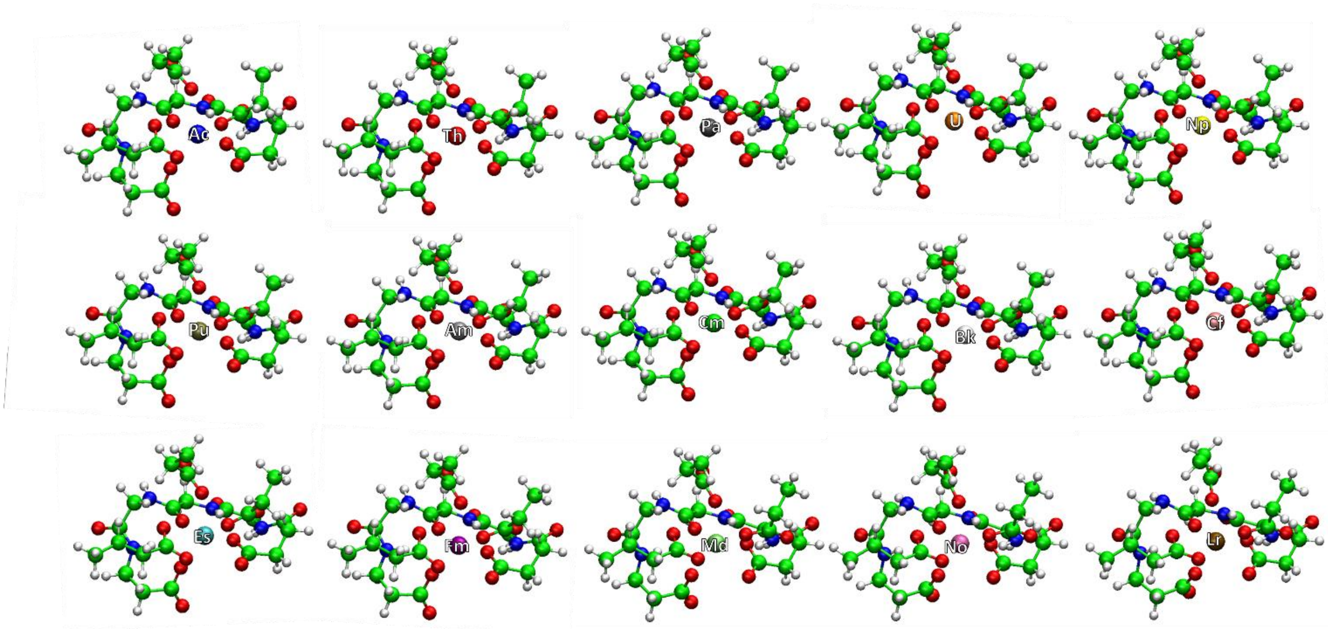

Figure S8. Optimized structures of An^{3+}- aquo-complexes.

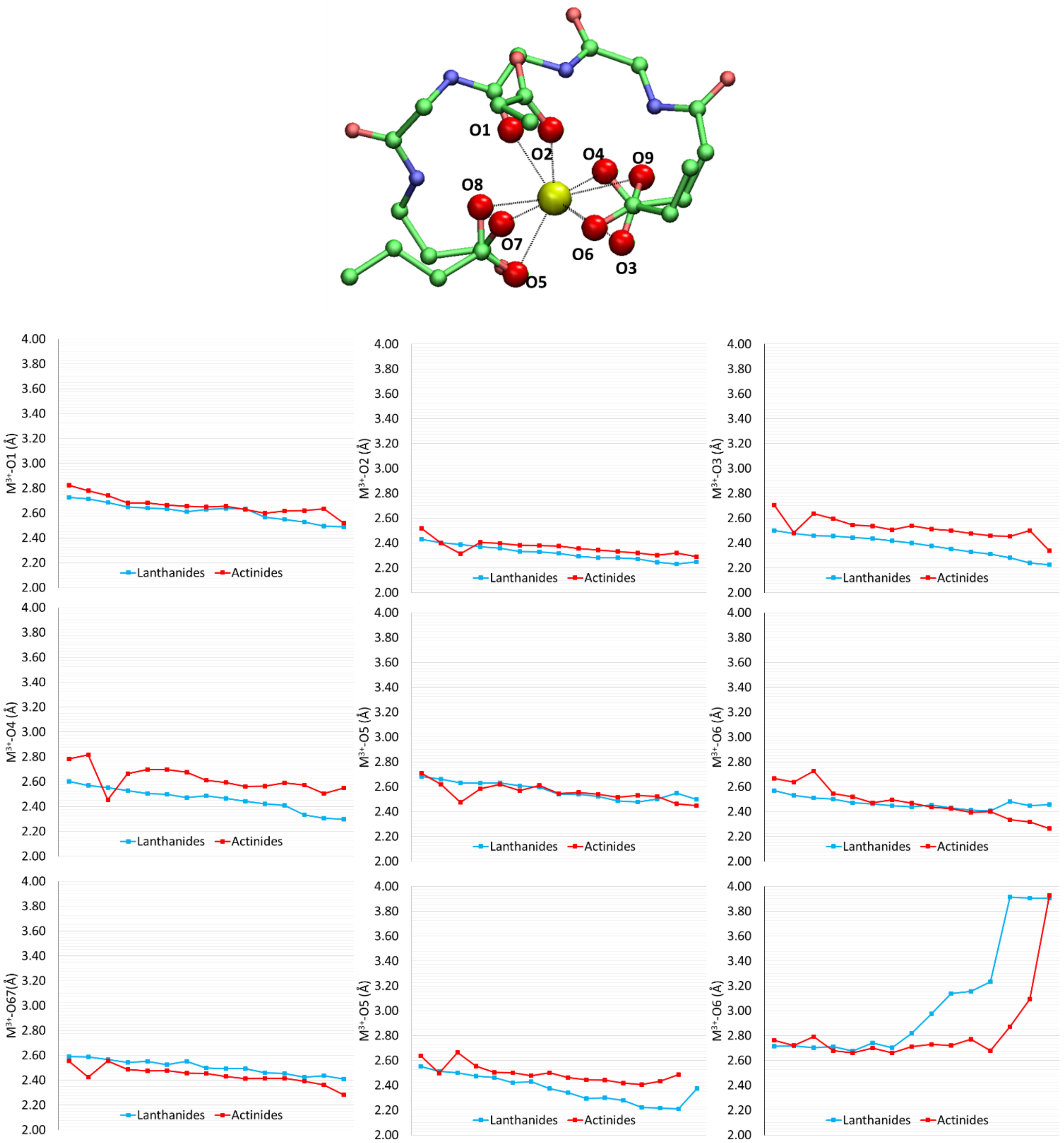

Figure S9. M^{3+}-O distances obtained for the optimized geometries of LanM-Ln^{3+} and LanM-An^{3+} series.

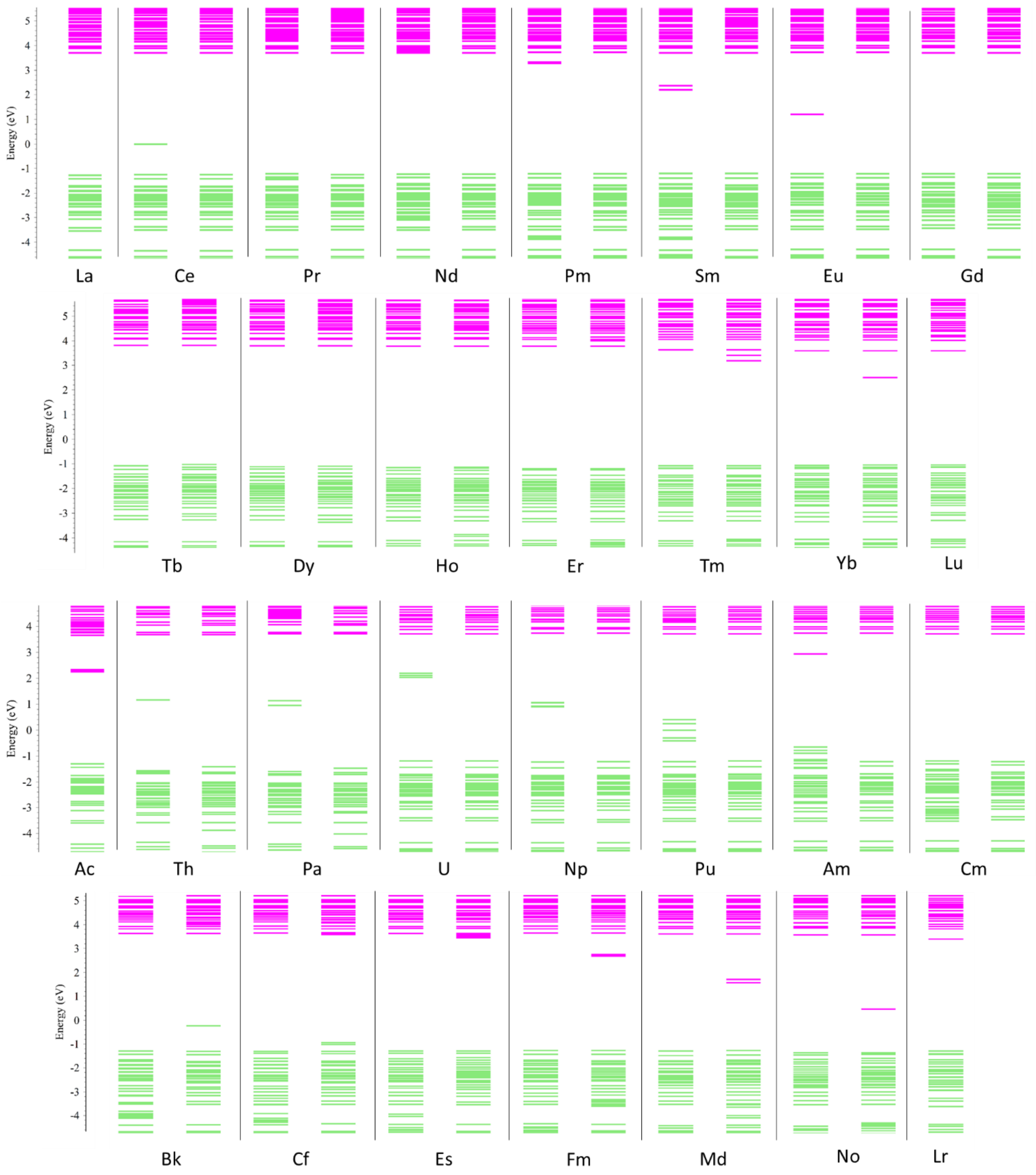

Figure S10. Molecular orbitals calculated for all Ln^{3+} and An^{3+} in complex with LanM.

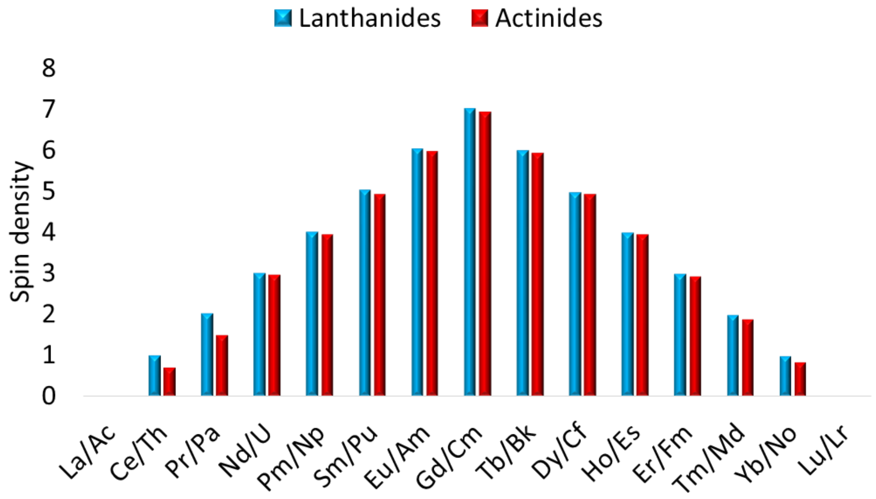

Figure S11. Calculated NBO spin density of metal ions, obtained for LanM-Ln^{3+} and LanM-An^{3+} complexes.

Table S1. Calculated spin contamination for unrestricted Kohn-Sham calculation on Ln-containing systems and selected 2S+1 multiplicities.

$$SC(\%) = \frac{\langle S^2 \rangle - \langle S_z \rangle (\langle S_z \rangle + 1)}{\langle S_z \rangle (\langle S_z \rangle + 1)} \cdot 100$$

$Ln(H_2O)_9^{3+}$	$<S^2>$	$<S_z>$	$<S_z>(<S_z>+1)$	SC (%)	2S+1
Ce	0.7506	0.5000	0.7500	0.0800	2
Pr	2.0013	1.0000	2.0000	0.0650	3
Nd	3.7522	1.5000	3.7500	0.0587	4
Pm	6.0034	2.0000	6.0000	0.0567	5
Sm	8.7559	2.5000	8.7500	0.0674	6
Eu	12.0085	3.0000	12.0000	0.0708	7
Gd	15.9695	3.5000	15.7500	1.3937	8
Tb	12.0215	3.0000	12.0000	0.1792	7
Dy	8.7558	2.5000	8.7500	0.0663	6
Ho	6.0033	2.0000	6.0000	0.0550	5
Er	3.7521	1.5000	3.7500	0.0560	4
Tm	2.0014	1.0000	2.0000	0.0700	3
Yb	0.7505	0.5000	0.7500	0.0667	2
**LanM-Ln^{3+}**	$<S^2>$	$<S_z>$	$<S_z>(<S_z>+1)$	**SC (%)**	**2S+1**
Ce	0.7508	0.5000	0.7500	0.1067	2
Pr	2.0019	1.0000	2.0000	0.0950	3
Nd	3.7531	1.5000	3.7500	0.0827	4
Pm	6.0058	2.0000	6.0000	0.0967	5
Sm	8.7631	2.5000	8.7500	0.1497	6
Eu	12.0225	3.0000	12.0000	0.1875	7
Gd	15.7577	3.5000	15.7500	0.0489	8
Tb	12.6063	3.0000	12.0000	5.0525	7
Dy	9.2846	2.5000	8.7500	6.1097	6
Ho	6.0037	2.0000	6.0000	0.0617	5
Er	3.7524	1.5000	3.7500	0.0640	4
Tm	2.0015	1.0000	2.0000	0.0750	3
Yb	0.7506	0.5000	0.7500	0.0800	2

Table S2. Calculated spin contamination for unrestricted Kohn-Sham calculation on An-containing systems and selected 2S+1 multiplicities.

$$SC(\%) = \frac{\langle S^2 \rangle - \langle S_z \rangle(\langle S_z \rangle + 1)}{\langle S_z \rangle(\langle S_z \rangle + 1)} \cdot 100$$

**$An(H_2O)_9^{3+}$**	**$<S^2>$**	**$<S_z>$**	**$<S_z>(<S_z>+1)$**	**SC (%)**	**2S+1**
Th	0.7514	0.5000	0.7500	0.1867	2
Pa	2.0016	1.0000	2.0000	0.0800	3
U	3.7519	1.5000	3.7500	0.0507	4
Np	6.0031	2.0000	6.0000	0.0517	5
Pu	8.7550	2.5000	8.7500	0.0571	6
Am	12.0063	3.0000	12.0000	0.0525	7
Cm	15.7559	3.5000	15.7500	0.0375	8
Bk	12.0053	3.0000	12.0000	0.0442	7
Cf	8.7541	2.5000	8.7500	0.0469	6
Es	6.0028	2.0000	6.0000	0.0467	5
Fm	3.7518	1.5000	3.7500	0.0480	4
Md	2.0011	1.0000	2.0000	0.0550	3
No	0.7504	0.5000	0.7500	0.0533	2
**$LanM\text{-}An^{3+}$**	**$<S^2>$**	**$<S_z>$**	**$<S_z>(<S_z>+1)$**	**SC (%)**	**2S+1**
Th	0.7531	0.5000	0.7500	0.4133	2
Pa	2.0059	1.0000	2.0000	0.2950	3
U	3.7529	1.5000	3.7500	0.0773	4
Np	6.0044	2.0000	6.0000	0.0733	5
Pu	8.758	2.5000	8.7500	0.0914	6
Am	12.011	3.0000	12.0000	0.0917	7
Cm	15.7571	3.5000	15.7500	0.0451	8
Bk	12.0065	3.0000	12.0000	0.0542	7
Cf	8.7549	2.5000	8.7500	0.0560	6
Es	6.0032	2.0000	6.0000	0.0533	5
Fm	3.7521	1.5000	3.7500	0.0560	4
Md	2.0013	1.0000	2.0000	0.0650	3
No	0.7507	0.5000	0.7500	0.0933	2

Table S3. Calculated NBO charges for all aquo- and LanM-complexes.

$Ln(H_2O)_9^{3+}$	NBO charge	$An(H_2O)_9^{3+}$	NBO charge
La	1.773	Ac	2.062
Ce	1.719	Th	1.520
Pr	1.684	Pa	1.463
Nd	1.691	U	1.482
Pm	1.684	Np	1.538
Sm	1.676	Pu	1.648
Eu	1.658	Am	1.736
Gd	1.517	Cm	1.722
Tb	1.642	Bk	1.737
Dy	1.656	Cf	1.716
Ho	1.614	Es	1.711
Er	1.618	Fm	1.704
Tm	1.613	Md	1.688
Yb	1.611	No	1.683
Lu	1.715	Lr	1.75
**LanM-Ln^{3+}**	**NBO charge**	**LanM-Ln^{3+}**	**NBO charge**
La	1.459	Ac	1.826
Ce	1.41	Th	1.786
Pr	1.381	Pa	1.552
Nd	1.364	U	1.304
Pm	1.3	Np	1.37
Sm	1.287	Pu	1.406
Eu	1.254	Am	1.622
Gd	1.205	Cm	1.656
Tb	1.129	Bk	1.641
Dy	1.163	Cf	1.623
Ho	1.264	Es	1.619
Er	1.231	Fm	1.603
Tm	1.35	Md	1.592
Yb	1.303	No	1.584
Lu	1.584	Lr	1.745

Table S4. Y^{3+}-ligand distances (in Å) comparison between NMR and optimized geometry of LanM-Y^{3+} structures.

	**PDB 6MI5**	**Optimized LanM-Y^{3+} 6-31G(d,p)**
Y^{3+}-$O1_{D35}$	2.21	2.31
Y^{3+}-$O1_{D37}$	2.36	2.36
Y^{3+}-$O2_{D37}$	2.39	3.07
Y^{3+}-$O1_{D39}$	2.28	2.38
Y^{3+}-$O2_{D39}$	2.32	2.49
Y^{3+}-O_{T41}	2.38	2.51
Y^{3+}-$O1_{D43}$	2.25	2.28
Y^{3+}-$O1_{E46}$	2.26	2.48
Y^{3+}-$O2_{E46}$	2.39	2.54
Average coordination sphere	2.32 ± 0.07	2.49 ± 0.24

Table S4. Absolute energies (in a.u) calculated for all the considered systems.

BS1=SDD for Ln or An and 6-31G(d,p) for other atoms.

BS2= SDD for Ln or An and 6-311+G(2d,2p) for other atoms

$H = E_{BS2} + ZPE_{BS1} + (E_{solv,BS1} - E_{BS1})$

ε=78.0 for aquo-complexes; ε=4.0 for LanM complexes

$Ln(H_2O)_9^{3+}$	E_{BS1}	ZPE_{BS1}	$E_{solv,BS1}$	E_{BS2}	H	
La	-1122.864565	0.222611	-1123.509174	-1123.141507	-1123.563504	
Ce	-1162.330585	0.222967	-1162.962483	-1162.607836	-1163.016766	
Pr	-1204.229025	0.223435	-1204.858468	-1204.505592	-1204.911600	
Nd	-1248.640030	0.223848	-1249.270826	-1248.916295	-1249.323244	
Pm	-1295.620980	0.224047	-1296.252209	-1295.896866	-1296.304048	
Sm	-1345.249958	0.224313	-1345.881189	-1345.525178	-1345.932096	
Eu	-1397.609494	0.224155	-1398.243316	-1397.884221	-1398.293888	
Gd	-1452.977549	0.218441	-1453.632760	-1453.252087	-1453.688857	
Tb	-1511.807888	0.224683	-1512.442828	-1512.082982	-1512.493240	
Dy	-1572.466949	0.224976	-1573.102138	-1572.739797	-1573.150010	
Ho	-1635.552618	0.225712	-1636.188385	-1635.872323	-1636.282379	
Er	-1702.685655	0.226136	-1703.321539	-1702.957001	-1703.366749	
Tm	-1772.931635	0.226160	-1773.565267	-1773.200078	-1773.607550	
Yb	-1846.522478	0.226321	-1847.169950	-1846.796952	-1847.218103	
Lu	-1923.487477	0.226635	-1924.113902	-1923.755146	-1924.154936	
**$LanM\text{-}Ln^{3+}$**	**E_{BS1}**	**ZPE_{BS1}**	**$E_{solv,BS1}$**	**E_{BS2}**	**H**	**ΔH (kcal/mol)**
La	-2605.924187	0.602225	-2606.118872	-2606.653806	-2606.246266	0.0
Ce	-2645.393029	0.602314	-2645.584161	-2646.123076	-2645.711895	-7.8
Pr	-2687.292387	0.602427	-2687.480659	-2688.022352	-2687.608197	-8.7
Nd	-2731.704782	0.602418	-2731.893656	-2732.434780	-2732.021236	-9.6
Pm	-2778.686472	0.602574	-2778.875421	-2779.416575	-2779.002950	-10.1
Sm	-2828.317188	0.602592	-2828.506069	-2829.047112	-2828.633401	-11.6
Eu	-2880.683730	0.602556	-2880.872527	-2881.413510	-2880.999750	-14.5
Gd	-2936.057495	0.602576	-2936.249128	-2936.787976	-2936.377033	-3.4
Tb	-2994.876967	0.602514	-2995.065923	-2995.606763	-2995.193205	-10.8
Dy	-3055.537283	0.602457	-3055.726658	-3056.265762	-3055.852680	-12.5
Ho	-3118.668158	0.603621	-3118.857890	-3119.396556	-3118.982667	-11.0
Er	-3185.747010	0.603104	-3185.935675	-3186.474664	-3186.060225	-6.7
Tm	-3256.004035	0.603108	-3256.192335	-3256.731561	-3256.316752	-16.6

Yb	-3329.605308	0.603163	-3329.794808	-3330.332145	-3329.918482	-11.1
Lu	-3406.572360	0.604298	-2606.118872	-3407.294153	-3406.875032	-23.4
**$An(H_2O)_9^{3+}$**	**E_{BS1}**	**ZPE_{BS1}**	**$E_{solv,BS1}$**	**E_{BS2}**	**H**	
Ac	-1062.944688	0.222228	-1063.634333	-1063.202348	-1063.669764	
Th	-1094.853882	0.218750	-1095.575667	-1095.11984	-1095.622875	
Pa	-1128.570063	0.222040	-1129.236205	-1128.841942	-1129.286044	
U	-1164.132660	0.222893	-1164.758677	-1164.405461	-1164.808585	
Np	-1201.653003	0.223346	-1202.307423	-1201.925062	-1202.356136	
Pu	-1241.127003	0.223189	-1241.778153	-1241.398956	-1241.826918	
Am	-1282.662367	0.223591	-1283.316880	-1282.934281	-1283.365204	
Cm	-1326.306498	0.223979	-1326.966680	-1326.578105	-1327.014308	
Bk	-1371.994280	0.224362	-1372.642950	-1372.260975	-1372.685283	
Cf	-1420.310631	0.224671	-1420.962387	-1420.576033	-1421.003117	
Es	-1470.910860	0.224670	-1471.563225	-1471.180924	-1471.608619	
Fm	-1523.830422	0.224714	-1524.481005	-1524.100225	-1524.526094	
Md	-1578.425625	0.224974	-1579.077782	-1578.694802	-1579.121985	
No	-1635.651695	0.225067	-1636.304853	-1635.920537	-1636.348628	
Lr	-1692.263387	0.226085	-1692.917709	-1692.534407	-1692.962644	
**$LanM\text{-}An^{3+}$**	**E_{BS1}**	**ZPE_{BS1}**	**$E_{solv,BS1}$**	**E_{BS2}**	**H**	**ΔH (kcal/mol)**
Ac	-2546.009731	0.601120	-2546.224517	-2546.706456	-2546.320122	0.0
Th	-2577.966378	0.600578	-2578.181974	-2578.681776	-2578.296794	-14.8
Pa	-2611.641010	0.599699	-2611.839976	-2612.355466	-2611.954733	-11.5
U	-2647.194377	0.601996	-2647.382184	-2647.917350	-2647.503161	-27.7
Np	-2684.719784	0.602267	-2684.918856	-2685.442687	-2685.039492	-20.7
Pu	-2724.194660	0.602190	-2724.391749	-2724.917529	-2724.512428	-22.1
Am	-2765.729909	0.602169	-2765.928162	-2766.453280	-2766.049363	-21.2
Cm	-2809.372103	0.602451	-2809.573049	-2810.095208	-2809.693703	-18.2
Bk	-2855.061977	0.602433	-2855.259522	-2855.785075	-2855.380186	-28.0
Cf	-2903.378299	0.602490	-2903.575430	-2904.101251	-2903.695893	-26.6
Es	-2953.980134	0.602452	-2954.177235	-2954.703021	-2954.297670	-24.3
Fm	-3006.901538	0.602461	-3007.098488	-3007.624548	-3007.219037	-26.7
Md	-3061.499259	0.602364	-3061.695211	-3062.222097	-3061.815685	-27.2
No	-3118.726808	0.602261	-3118.923899	-3119.449534	-3119.044365	-28.5
Lr	-3175.332982	0.602589	-3175.530220	-3176.058910	-3175.653560	-25.5

Cartesian coordinates

$La(H_2O)_9^{3+}$

La	0.000	0.000	0.000
O	1.862	-1.283	-1.282
H	2.669	-0.934	-1.693
O	-0.006	0.671	-2.526
H	-0.537	1.367	-2.944
O	-1.868	-1.284	-1.275
H	-2.674	-1.691	-0.922
O	-1.859	-0.466	1.756
H	-1.911	-1.254	2.320
O	0.002	-2.524	0.680
H	0.533	-2.941	1.377
O	-1.866	1.749	-0.468
H	-1.918	2.630	-0.067
O	0.004	1.854	1.842
H	0.535	2.665	1.852
O	1.864	1.749	-0.476
H	1.915	2.310	-1.267
H	1.915	-2.248	-1.372
H	0.523	0.276	-3.237
H	-1.923	-1.377	-2.239
H	-0.529	-3.235	0.288
H	2.673	1.929	0.028
H	-0.523	1.870	2.657
H	-2.675	1.649	-0.994
H	-2.666	0.041	1.936
O	1.867	-0.466	1.748
H	2.674	-0.994	1.649
H	1.922	-0.062	2.629

$Ce(H_2O)_9^{3+}$

Ce	0.000	0.000	0.000
O	-1.856	-1.375	1.137
H	-2.663	-1.057	1.572
O	-0.002	0.454	2.547
H	0.528	1.112	3.023
O	1.855	-1.356	1.162
H	2.662	-1.730	0.775
O	1.855	-0.327	-1.755
H	1.909	-1.064	-2.384
O	0.001	-2.433	-0.879
H	-0.528	-2.790	-1.609
O	1.855	1.684	0.595
H	1.909	2.597	0.271
O	0.001	1.980	-1.665
H	-0.530	2.790	-1.607
O	-1.855	1.675	0.620
H	-1.910	2.170	1.452
H	-1.910	-2.344	1.150
H	-0.533	-0.001	3.220
H	1.908	-1.534	2.115
H	0.531	-3.174	-0.545
H	-2.661	1.892	0.126
H	0.530	2.063	-2.474
H	2.661	1.536	1.113
H	2.661	0.196	-1.886
O	-1.853	-0.301	-1.762
H	-2.660	-0.836	-1.703
H	-1.907	0.172	-2.608

$Pr(H_2O)_9^{3+}$

Pr	0.000	0.000	0.000
O	-1.836	-0.290	1.743
H	-2.641	0.237	1.869
O	0.004	1.998	1.625
H	0.530	2.809	1.551
O	1.831	-0.274	1.751
H	2.635	-0.815	1.707
O	1.831	-1.384	-1.107
H	1.881	-2.353	-1.097
O	-0.007	-2.407	0.916
H	-0.531	-3.154	0.586
O	1.841	1.642	-0.639
H	1.896	2.119	-1.482
O	0.003	0.413	-2.542
H	-0.519	1.072	-3.026
O	-1.830	1.662	-0.618
H	-1.879	2.579	-0.304
H	-1.890	-1.020	2.379
H	-0.519	2.090	2.437
H	1.884	0.219	2.585
H	0.513	-2.751	1.659
H	-2.637	1.512	-1.136
H	0.525	-0.059	-3.209
H	2.648	1.868	-0.149
H	2.639	-1.077	-1.548
O	-1.836	-1.360	-1.129
H	-2.644	-1.728	-0.739
H	-1.887	-1.547	-2.080

$Nd(H_2O)_9^{3+}$

Nd	0.000	-0.005	-0.001
O	1.962	0.862	-1.369
H	2.752	1.329	-1.056
O	0.004	2.558	0.015
H	-0.581	3.138	0.528
O	-1.698	0.912	-1.643
H	-2.478	0.477	-2.022
O	-1.813	-1.762	-0.124
H	-1.836	-2.508	-0.744
O	0.145	-1.251	-2.218
H	0.685	-2.034	-2.410
O	-1.960	0.854	1.377
H	-2.086	0.709	2.328
O	-0.149	-1.268	2.208
H	0.325	-1.065	3.029
O	1.705	0.889	1.649
H	1.725	1.800	1.982
H	2.089	0.728	-2.322
H	0.590	3.143	-0.491
H	-1.715	1.826	-1.967
H	-0.327	-1.039	-3.038
H	2.483	0.447	2.023
H	-0.693	-2.050	2.394
H	-2.748	1.328	1.070
H	-2.654	-1.786	0.359
O	1.804	-1.772	0.109
H	2.645	-1.798	-0.374
H	1.823	-2.522	0.725

$Pm(H_2O)_9^{3+}$

Pm	-0.005	0.000	0.000
O	-1.819	-1.709	-0.391
H	-2.626	-1.864	0.125
O	-0.005	-1.711	1.876
H	0.522	-1.681	2.690
O	1.809	-1.705	-0.356
H	2.618	-1.623	-0.886
O	1.820	1.161	-1.286
H	1.884	1.196	-2.253
O	0.018	-0.780	-2.416
H	-0.502	-0.418	-3.152

O	1.801	0.530	1.668
H	1.861	1.348	2.185
O	0.016	2.483	0.532
H	-0.508	2.939	1.209
O	-1.812	0.534	1.676
H	-1.868	0.169	2.573
H	-1.870	-2.304	-1.156
H	-0.534	-2.522	1.923
H	1.866	-2.562	0.096
H	0.548	-1.502	-2.787
H	-2.614	1.066	1.551
H	0.551	3.164	0.096
H	2.605	0.024	1.866
H	2.625	1.580	-0.944
O	-1.804	1.196	-1.302
H	-2.607	0.827	-1.702
H	-1.851	2.155	-1.436

$Sm(H_2O)_9^{3+}$

Sm	0.000	0.000	-0.004
O	1.758	-0.160	1.778
H	2.498	-0.784	1.851
O	-0.236	-2.200	1.228
H	-0.855	-2.915	1.011
O	-1.765	0.185	1.768
H	-2.505	0.810	1.828
O	-1.641	1.668	-0.868
H	-1.600	2.623	-0.704
O	0.233	2.217	1.198
H	0.852	2.929	0.974
O	-1.969	-1.295	-0.858
H	-2.073	-1.625	-1.765
O	0.004	-0.018	-2.515
H	0.473	-0.642	-3.091
O	1.645	-1.679	-0.840
H	1.604	-2.632	-0.663
H	1.860	0.465	2.513
H	0.253	-2.493	2.013
H	-1.870	-0.427	2.513
H	-0.259	2.521	1.977
H	2.479	-1.522	-1.311
H	-0.464	0.597	-3.101
H	-2.797	-1.500	-0.398
H	-2.473	1.506	-1.340

O	1.971	1.283	-0.871
H	2.799	1.495	-0.412
H	2.076	1.599	-1.783

$Eu(H_2O)_9^{3+}$

Eu	0.000	0.000	0.000
O	1.781	1.283	-1.194
H	2.588	1.673	-0.822
O	0.003	2.390	0.728
H	-0.528	2.779	1.439
O	-1.789	1.273	-1.194
H	-2.595	0.925	-1.607
O	-1.788	-1.674	-0.498
H	-1.849	-2.213	-1.302
O	-0.007	-0.565	-2.433
H	0.526	-1.246	-2.871
O	-1.779	0.403	1.708
H	-1.835	-0.023	2.577
O	0.004	-1.827	1.704
H	0.539	-1.865	2.512
O	1.790	0.387	1.701
H	1.850	1.153	2.293
H	1.837	1.413	-2.154
H	0.536	3.109	0.353
H	-1.847	2.239	-1.260
H	-0.543	-0.145	-3.124
H	2.594	-0.134	1.850
H	-0.531	-2.636	1.686
H	-2.584	0.937	1.616
H	-2.591	-1.860	0.014
O	1.784	-1.670	-0.521
H	2.588	-1.542	-1.050
H	1.843	-2.567	-0.154

$Gd(H_2O)_9^{3+}$

Gd	-0.013	-0.001	0.001
O	-1.568	-0.091	1.953
H	-2.436	0.319	2.103
O	-0.036	2.175	1.326
H	0.576	2.923	1.238
O	1.967	0.001	1.542
H	2.736	-0.581	1.437
O	1.786	-1.414	-1.024
H	1.832	-2.381	-1.099
O	0.047	-2.232	1.227
H	-0.412	-3.019	0.891
O	1.733	1.524	-0.953
H	1.694	2.070	-1.755
O	-0.301	0.054	-2.529
H	-0.796	0.758	-2.979
O	-1.812	1.575	-0.722
H	-1.821	2.458	-0.318
H	-1.533	-0.880	2.518
H	-0.411	2.234	2.220
H	2.061	0.422	2.411
H	0.734	-2.555	1.831
H	-2.708	1.415	-1.060
H	0.287	-0.335	-3.197
H	2.533	1.796	-0.477
H	2.503	-1.063	-1.576
O	-1.757	-1.588	-0.825
H	-2.592	-1.886	-0.429
H	-1.856	-1.682	-1.786

$Tb(H_2O)_9^{3+}$

Tb	0.011	0.000	0.000
O	1.783	-0.541	-1.643
H	2.587	-0.039	-1.848
O	0.000	1.646	-1.851
H	-0.556	2.439	-1.907
O	-1.816	-0.556	-1.604
H	-2.622	-1.066	-1.430
O	-1.811	-1.118	1.285
H	-1.873	-2.067	1.474
O	0.008	-2.427	-0.496
H	0.537	-3.113	-0.059
O	-1.814	1.667	0.330
H	-1.877	2.306	1.057
O	0.011	0.779	2.350
H	0.537	1.502	2.727
O	1.787	1.691	0.346
H	1.838	2.548	-0.106
H	1.835	-1.362	-2.157
H	0.522	1.613	-2.667
H	-1.882	-0.246	-2.521
H	-0.547	-2.877	-1.151
H	2.595	1.614	0.877
H	-0.541	0.433	3.069
H	-2.623	1.773	-0.195

H	-2.617	-0.716	1.644
O	1.795	-1.143	1.283
H	2.601	-1.566	0.946
H	1.851	-1.181	2.251

$Dy(H_2O)_9^{3+}$

Dy	-0.005	0.000	0.000
O	1.771	1.173	-1.239
H	2.574	1.593	-0.892
O	-0.022	2.429	0.540
H	-0.548	2.870	1.224
O	-1.754	1.168	-1.264
H	-2.554	0.798	-1.668
O	-1.752	-1.676	-0.408
H	-1.793	-2.257	-1.184
O	0.018	-0.753	-2.370
H	0.562	-1.464	-2.742
O	-1.768	0.486	1.638
H	-1.820	0.101	2.527
O	-0.008	-1.690	1.824
H	0.518	-1.663	2.639
O	1.754	0.509	1.645
H	1.800	1.319	2.178
H	1.829	1.226	-2.206
H	0.511	3.118	0.113
H	-1.807	2.130	-1.379
H	-0.500	-0.388	-3.104
H	2.564	0.009	1.834
H	-0.528	-2.508	1.858
H	-2.574	1.012	1.517
H	-2.560	-1.842	0.104
O	1.790	-1.647	-0.366
H	2.601	-1.551	-0.890
H	1.846	-2.512	0.070

$Ho(H_2O)_9^{3+}$

Ho	0.000	0.001	0.000
O	-1.741	1.693	0.185
H	-2.549	1.784	-0.344
O	-0.018	1.423	-2.030
H	0.500	1.288	-2.839
O	1.745	1.696	0.114
H	2.553	1.694	0.651
O	1.756	-0.962	1.387
H	1.818	-0.862	2.350
O	0.022	1.047	2.248
H	-0.497	0.773	3.021
O	1.727	-0.738	-1.550
H	1.773	-1.623	-1.944
O	-0.003	-2.470	-0.218
H	-0.537	-2.998	-0.833
O	-1.759	-0.698	-1.534
H	-1.819	-0.432	-2.465
H	-1.789	2.367	0.881
H	-0.545	2.225	-2.171
H	1.794	2.479	-0.456
H	0.550	1.811	2.527
H	-2.560	-1.208	-1.339
H	0.528	-3.099	0.295
H	2.528	-0.268	-1.831
H	2.556	-1.432	1.105
O	-1.730	-0.994	1.397
H	-2.528	-0.579	1.758
H	-1.778	-1.935	1.629

$Er(H_2O)_9^{3+}$

Er	-0.002	0.000	0.000
O	1.736	-0.419	-1.629
H	2.537	0.099	-1.807
O	-0.001	1.767	-1.734
H	-0.527	2.582	-1.733
O	-1.718	-0.426	-1.657
H	-2.518	-0.969	-1.574
O	-1.725	-1.235	1.175
H	-1.767	-2.202	1.233
O	0.025	-2.385	-0.663
H	0.550	-3.093	-0.259
O	-1.741	1.624	0.460
H	-1.796	2.157	1.269
O	-0.009	0.616	2.397
H	0.505	1.324	2.817
O	1.712	1.646	0.453
H	1.760	2.518	0.032
H	1.795	-1.219	-2.174
H	0.523	1.779	-2.550
H	-1.765	0.007	-2.524
H	-0.491	-2.796	-1.375
H	2.514	1.551	0.989
H	-0.529	0.199	3.102
H	-2.543	1.813	-0.051
H	-2.532	-0.897	1.595
O	1.732	-1.189	1.196
H	2.540	-1.593	0.844
H	1.782	-1.263	2.162

$Tm(H_2O)_9^{3+}$

Tm	0.000	-0.003	0.000
O	1.601	-0.839	1.589
H	1.978	-0.389	2.361
O	2.128	1.269	-0.158
H	2.289	2.069	-0.682
O	1.341	-0.785	-1.837
H	1.059	-1.269	-2.629
O	-1.565	-0.888	-1.598
H	-1.855	-1.813	-1.628
O	0.049	-2.473	-0.010
H	-0.469	-3.066	0.557
O	-0.184	1.708	-1.683
H	-0.846	2.417	-1.672
O	-2.178	1.181	0.168
H	-2.373	1.970	0.698
O	0.116	1.700	1.697
H	0.749	2.435	1.693
H	1.926	-1.752	1.611
H	2.963	1.066	0.293
H	2.280	-0.574	-1.957
H	0.589	-3.040	-0.582
H	-0.365	1.750	2.537
H	-3.002	0.947	-0.287
H	0.293	1.785	-2.523
H	-1.958	-0.447	-2.367
O	-1.308	-0.852	1.831
H	-1.007	-1.331	2.619
H	-2.254	-0.680	1.953

$Yb(H_2O)_9^{3+}$

Yb	0.000	0.003	0.000
O	0.108	-1.643	-1.712
H	-0.404	-1.704	-2.533
O	-2.167	-1.225	-0.137
H	-2.983	-0.998	0.336
O	-0.127	-1.705	1.648
H	0.384	-1.803	2.467
O	1.378	0.787	1.803

H	2.321	0.579	1.889
O	2.153	-1.253	0.089
H	2.971	-1.018	-0.376
O	-1.535	0.828	1.641
H	-1.842	1.747	1.694
O	0.016	2.451	0.047
H	-0.529	3.045	-0.494
O	-1.369	0.872	-1.770
H	-2.315	0.678	-1.864
H	0.770	-2.352	-1.737
H	-2.374	-2.008	-0.672
H	-0.797	-2.407	1.646
H	2.352	-2.057	0.595
H	-1.107	1.373	-2.558
H	0.567	3.016	0.611
H	-1.885	0.371	2.421
H	1.122	1.260	2.610
O	1.543	0.872	-1.610
H	1.888	0.440	-2.407
H	1.861	1.788	-1.629

$Lu(H_2O)_9^{3+}$

Lu	0.000	0.000	0.000
O	0.080	1.685	1.682
H	-0.456	1.766	2.487
O	-2.089	1.314	0.000
H	-2.891	1.128	-0.513
O	0.050	1.677	-1.692
H	0.590	1.710	-2.497
O	1.426	-0.891	-1.688
H	2.381	-0.726	-1.734
O	2.184	1.149	-0.007
H	2.970	0.905	0.507
O	-1.485	-0.799	-1.684
H	-1.820	-1.709	-1.725
O	-0.096	-2.465	0.007
H	-0.699	-3.022	0.525
O	-1.490	-0.768	1.694
H	-2.429	-0.529	1.739
H	0.755	2.380	1.722
H	-2.269	2.119	0.511
H	-0.569	2.422	-1.737
H	2.426	1.933	-0.524
H	-1.287	-1.269	2.499
H	0.463	-3.069	-0.506
H	-1.788	-0.351	-2.489
H	1.185	-1.380	-2.490
O	1.420	-0.902	1.688
H	1.756	-0.472	2.490
H	1.685	-1.833	1.735

$Ac(H_2O)_9^{3+}$

Ac	0.000	0.000	0.000
O	1.954	0.416	-1.826
H	2.762	0.947	-1.733
O	-0.001	2.583	-0.807
H	-0.545	3.307	-0.457
O	-1.949	0.397	-1.834
H	-2.757	-0.122	-1.976
O	-1.957	-1.782	0.563
H	-2.008	-2.698	0.243
O	0.006	-1.999	-1.824
H	0.550	-2.803	-1.810
O	-1.960	1.381	1.257
H	-2.015	1.560	2.209
O	-0.005	-0.592	2.640
H	0.534	-0.181	3.335
O	1.951	1.376	1.275
H	2.002	2.340	1.373
H	2.007	0.018	-2.710
H	0.539	2.980	-1.509
H	-2.000	1.132	-2.468
H	-0.532	-2.062	-2.629
H	2.760	1.031	1.687
H	-0.547	-1.259	3.091
H	-2.768	1.760	0.875
H	-2.767	-1.644	1.080
O	1.960	-1.781	0.558
H	2.770	-1.963	0.054
H	2.013	-2.348	1.344

$Th(H_2O)_9^{3+}$

Th	0.000	0.000	0.001
O	2.005	-1.551	0.750
H	2.829	-1.725	0.266
O	0.005	-2.210	-1.320
H	-0.554	-2.433	-2.082
O	-1.998	-1.461	0.924
H	-2.822	-1.146	1.330
O	-2.005	1.526	0.799
H	-2.054	2.025	1.630
O	0.000	-0.041	2.574
H	0.559	0.493	3.162
O	-1.997	-0.076	-1.728
H	-2.044	0.396	-2.575
O	-0.006	2.251	-1.249
H	0.554	2.498	-2.003
O	1.997	0.131	-1.725
H	2.044	-0.314	-2.586
H	2.054	-2.075	1.565
H	0.569	-2.983	-1.151
H	-2.044	-2.431	0.941
H	-0.559	-0.593	3.145
H	2.819	0.641	-1.639
H	-0.569	3.018	-1.056
H	-2.819	-0.588	-1.658
H	-2.830	1.715	0.321
O	1.999	1.431	0.970
H	2.823	1.103	1.365
H	2.044	2.400	1.017

$Pa(H_2O)_9^{3+}$

Pa	0.000	0.000	0.000
O	1.872	-1.091	-1.451
H	2.684	-0.691	-1.802
O	0.004	1.081	-2.405
H	-0.503	1.854	-2.700
O	-1.875	-1.031	-1.490
H	-2.687	-1.489	-1.219
O	-1.876	-0.770	1.639
H	-1.920	-1.641	2.063
O	-0.005	-2.624	0.269
H	0.502	-3.147	0.910
O	-1.872	1.809	-0.152
H	-1.914	2.612	0.391
O	0.003	1.548	2.135
H	0.513	2.363	2.265
O	1.879	1.796	-0.216
H	1.923	2.472	-0.910
H	1.914	-2.030	-1.690
H	0.511	0.786	-3.178
H	-1.918	-0.964	-2.457
H	-0.513	-3.265	-0.253

H	2.689	1.898	0.308
H	-0.504	1.420	2.953
H	-2.682	1.806	-0.686
H	-2.685	-0.303	1.904
O	1.871	-0.718	1.669
H	2.681	-1.224	1.497
H	1.914	-0.456	2.602

$U(H_2O)_9^{3+}$

U	0.000	-0.013	-0.024
O	1.739	-1.918	0.018
H	2.531	-2.053	-0.526
O	-0.004	-1.274	-2.263
H	-0.493	-1.037	-3.067
O	-1.907	-1.741	0.045
H	-2.725	-1.666	0.562
O	-1.957	0.829	1.502
H	-2.001	0.685	2.460
O	0.011	-1.187	2.307
H	0.595	-0.972	3.051
O	-1.739	1.016	-1.627
H	-1.756	1.953	-1.875
O	-0.007	2.587	0.186
H	0.546	3.225	-0.293
O	1.904	0.941	-1.469
H	2.001	0.892	-2.434
H	1.756	-2.619	0.689
H	0.485	-2.084	-2.477
H	-2.007	-2.539	-0.499
H	-0.542	-1.927	2.603
H	2.723	1.342	-1.137
H	-0.589	3.111	0.758
H	-2.532	0.622	-2.025
H	-2.760	1.321	1.269
O	1.961	0.850	1.484
H	2.764	0.395	1.783
H	2.007	1.744	1.858

$Np(H_2O)_9^{3+}$

Np	0.000	0.000	0.000
O	1.822	0.890	1.560
H	2.630	0.454	1.874
O	0.008	-1.427	2.181
H	-0.512	-2.227	2.357
O	-1.835	0.722	1.628
H	-2.643	1.231	1.456
O	-1.835	1.031	-1.452
H	-1.884	1.971	-1.689
O	-0.019	2.602	0.145
H	0.501	3.221	-0.392
O	-1.817	-1.791	-0.180
H	-1.853	-2.465	-0.876
O	0.011	-1.177	-2.325
H	0.541	-1.947	-2.587
O	1.840	-1.777	-0.017
H	1.886	-2.522	0.602
H	1.857	1.799	1.897
H	0.530	-1.266	2.983
H	-1.877	0.456	2.560
H	-0.547	3.150	0.747
H	2.647	-1.823	-0.555
H	-0.514	-0.937	-3.105
H	-2.621	-1.905	0.350
H	-2.636	0.620	-1.813
O	1.822	0.925	-1.539
H	2.623	1.423	-1.313
H	1.865	0.762	-2.494

$Pu(H_2O)_9^{3+}$

Pu	0.000	-0.001	0.005
O	1.613	0.729	-1.844
H	2.139	1.541	-1.911
O	-0.892	2.188	-1.039
H	-1.691	2.659	-0.753
O	-1.620	-0.536	-1.902
H	-2.144	-1.338	-2.054
O	-1.173	-2.131	0.718
H	-0.867	-3.026	0.504
O	0.888	-2.070	-1.256
H	1.688	-2.565	-1.020
O	-2.298	0.644	0.942
H	-2.475	0.860	1.871
O	0.007	-0.134	2.552
H	0.303	0.552	3.171
O	1.173	2.049	0.924
H	0.863	2.960	0.800
H	1.874	0.182	-2.602
H	-0.551	2.676	-1.805
H	-1.884	0.087	-2.598
H	0.546	-2.481	-2.066
H	2.028	2.109	1.378
H	-0.289	-0.882	3.095
H	-3.159	0.651	0.496
H	-2.029	-2.233	1.163
O	2.303	-0.734	0.862
H	3.163	-0.693	0.415
H	2.482	-1.042	1.765

$Am(H_2O)_9^{3+}$

Am	0.000	0.005	0.000
O	-2.085	-0.930	-1.144
H	-2.800	-1.471	-0.774
O	0.028	-2.583	0.016
H	0.661	-3.158	0.475
O	1.383	-0.910	-1.910
H	2.091	-0.480	-2.415
O	1.815	1.709	-0.470
H	1.730	2.450	-1.091
O	-0.531	1.375	-2.086
H	-1.132	2.136	-2.114
O	2.105	-0.872	1.153
H	2.362	-0.662	2.065
O	0.503	1.409	2.069
H	0.202	1.271	2.982
O	-1.366	-0.914	1.921
H	-1.317	-1.828	2.243
H	-2.343	-0.739	-2.059
H	-0.595	-3.177	-0.433
H	1.353	-1.830	-2.217
H	-0.227	1.233	-2.996
H	-2.084	-0.491	2.418
H	1.087	2.183	2.088
H	2.830	-1.404	0.790
H	2.736	1.712	-0.165
O	-1.851	1.676	0.451
H	-2.771	1.657	0.143
H	-1.782	2.426	1.062

$Cm(H_2O)_9^{3+}$

Cm	0.000	0.000	0.000
O	-1.813	-0.655	-1.642
H	-2.621	-1.168	-1.483
O	-0.007	-2.529	-0.367
H	0.523	-3.186	0.110

O	1.796	-0.655	-1.661
H	2.600	-0.172	-1.907
O	1.812	1.752	0.255
H	1.862	2.574	-0.258
O	-0.009	1.595	-1.996
H	-0.538	2.403	-2.085
O	1.816	-1.105	1.377
H	1.872	-1.076	2.345
O	0.012	0.940	2.374
H	-0.506	0.621	3.130
O	-1.803	-1.097	1.399
H	-1.855	-2.046	1.590
H	-1.866	-0.347	-2.561
H	-0.535	-3.023	-1.014
H	1.842	-1.508	-2.122
H	0.507	1.518	-2.814
H	-2.609	-0.702	1.769
H	0.540	1.685	2.702
H	2.621	-1.552	1.073
H	2.623	1.717	0.787
O	-1.808	1.754	0.267
H	-2.619	1.878	-0.251
H	-1.853	2.393	0.996

$Bk(H_2O)_9^{3+}$

Bk	0.000	0.000	0.000
O	1.795	-0.771	-1.582
H	2.605	-0.309	-1.852
O	0.004	1.445	-2.085
H	-0.516	2.250	-2.237
O	-1.804	-0.726	-1.593
H	-2.613	-1.227	-1.407
O	-1.800	-1.009	1.436
H	-1.851	-1.946	1.681
O	-0.011	-2.529	-0.211
H	0.510	-3.159	0.311
O	-1.791	1.757	0.164
H	-1.835	2.439	0.851
O	0.007	1.085	2.294
H	0.534	1.850	2.573
O	1.806	1.745	0.126
H	1.856	2.531	-0.439
H	1.840	-1.654	-1.982
H	0.527	1.302	-2.890
H	-1.852	-0.471	-2.528
H	-0.536	-3.058	-0.832
H	2.614	1.744	0.664
H	-0.515	0.817	3.066
H	-2.600	1.850	-0.365
H	-2.605	-0.593	1.783
O	1.795	-0.996	1.451
H	2.601	-1.465	1.183
H	1.844	-0.901	2.416

$Cf(H_2O)_9^{3+}$

Cf	0.000	0.000	0.000
O	-1.856	-0.913	-1.381
H	-2.672	-1.352	-1.091
O	-0.080	-2.525	0.195
H	0.436	-3.081	0.799
O	1.689	-1.056	-1.486
H	2.495	-0.672	-1.866
O	1.832	1.669	-0.208
H	1.879	2.348	-0.900
O	-0.042	1.100	-2.279
H	-0.541	1.895	-2.527
O	1.801	-0.799	1.516
H	1.887	-0.547	2.449
O	0.119	1.437	2.081
H	-0.367	1.308	2.912
O	-1.742	-0.709	1.625
H	-1.799	-1.599	2.006
H	-1.932	-0.801	-2.341
H	-0.634	-3.129	-0.324
H	1.692	-1.995	-1.730
H	0.430	0.815	-3.078
H	-2.522	-0.225	1.941
H	0.669	2.225	2.208
H	2.584	-1.332	1.303
H	2.661	1.729	0.292
O	-1.721	1.795	-0.061
H	-2.548	1.833	-0.567
H	-1.720	2.570	0.522

$Es(H_2O)_9^{3+}$

Es	0.000	0.004	0.003
O	-1.802	1.592	0.593
H	-2.691	1.662	0.210
O	-0.416	1.594	-1.895
H	-0.028	1.553	-2.783
O	1.659	1.836	0.039
H	2.544	1.842	0.438
O	2.073	-0.975	0.988
H	2.287	-0.991	1.935
O	0.322	0.839	2.342
H	-0.042	0.450	3.153
O	1.515	-0.491	-1.900
H	1.531	-1.340	-2.370
O	0.091	-2.475	-0.471
H	-0.516	-2.983	-1.033
O	-2.007	-0.693	-1.306
H	-2.226	-0.368	-2.194
H	-1.779	2.200	1.348
H	-0.997	2.371	-1.884
H	1.575	2.665	-0.457
H	0.841	1.611	2.616
H	-2.748	-1.255	-1.031
H	0.740	-3.109	-0.126
H	2.233	0.044	-2.274
H	2.849	-1.332	0.528
O	-1.433	-1.260	1.583
H	-2.183	-0.954	2.117
H	-1.382	-2.221	1.706

$Fm(H_2O)_9^{3+}$

Fm	0.004	0.000	0.000
O	1.767	-0.948	1.463
H	2.573	-1.426	1.214
O	-0.004	-2.503	-0.157
H	-0.521	-3.042	-0.776
O	-1.757	-0.966	1.432
H	-2.568	-0.550	1.764
O	-1.770	1.711	0.116
H	-1.827	2.388	0.808
O	-0.019	1.115	2.245
H	0.497	1.893	2.513
O	-1.756	-0.768	-1.549
H	-1.810	-0.508	-2.482
O	-0.019	1.391	-2.086
H	0.499	1.239	-2.892
O	1.765	-0.782	-1.558
H	1.812	-1.677	-1.929
H	1.810	-0.821	2.423

H	0.516	-3.121	0.381
H	-1.808	-1.904	1.673
H	-0.537	0.847	3.020
H	2.569	-0.322	-1.850
H	-0.543	2.193	-2.241
H	-2.563	-1.270	-1.353
H	-2.579	1.788	-0.414
O	1.758	1.749	0.096
H	2.564	1.774	0.636
H	1.800	2.518	-0.493

$Md(H_2O)_9^{3+}$

Md	0.001	0.005	-0.001
O	-0.244	-1.787	1.663
H	0.130	-1.847	2.556
O	2.255	-1.044	0.447
H	3.110	-0.745	0.101
O	0.506	-1.745	-1.647
H	0.145	-1.869	-2.539
O	-1.142	0.714	-2.045
H	-2.041	0.433	-2.276
O	-2.083	-1.360	-0.435
H	-2.968	-1.184	-0.079
O	1.737	1.021	-1.404
H	1.957	1.966	-1.401
O	-0.202	2.495	-0.016
H	0.199	3.116	0.612
O	1.025	0.902	2.030
H	1.956	0.771	2.270
H	-0.838	-2.548	1.564
H	2.439	-1.833	0.980
H	1.206	-2.408	-1.541
H	-2.159	-2.175	-0.956
H	0.598	1.329	2.789
H	-0.690	3.029	-0.662
H	2.259	0.617	-2.116
H	-0.791	1.190	-2.814
O	-1.857	0.763	1.416
H	-2.313	0.292	2.132
H	-2.219	1.662	1.399

$No(H_2O)_9^{3+}$

No	-0.010	0.000	-0.001
O	0.910	-2.286	0.046
H	1.449	-2.717	0.727
O	2.545	0.042	0.016
H	3.121	0.816	0.108
O	0.911	0.243	-2.244
H	0.504	0.599	-3.051
O	-1.684	1.268	-1.251
H	-2.393	0.861	-1.773
O	-1.300	-1.475	-1.542
H	-2.068	-2.009	-1.288
O	0.825	2.315	-0.032
H	0.607	2.987	0.632
O	-1.374	1.422	1.528
H	-1.208	1.629	2.461
O	0.889	-0.199	2.257
H	1.818	-0.019	2.472
H	0.723	-2.963	-0.623
H	3.136	-0.722	-0.072
H	1.848	0.096	-2.448
H	-1.123	-1.672	-2.476
H	0.487	-0.575	3.056
H	-2.159	1.928	1.267
H	1.364	2.761	-0.704
H	-1.700	2.218	-1.445
O	-1.648	-1.329	1.234
H	-1.632	-2.280	1.428
H	-2.375	-0.947	1.749

$Lr(H_2O)_9^{3+}$

Lr	0.000	0.000	0.000
O	0.680	1.565	1.732
H	0.209	1.820	2.541
O	-1.513	1.980	0.023
H	-2.329	2.093	-0.489
O	0.641	1.596	-1.719
H	1.155	1.445	-2.528
O	1.034	-1.343	-1.744
H	1.984	-1.532	-1.797
O	2.472	0.316	-0.014
H	3.125	-0.188	0.496
O	-1.707	-0.216	-1.718
H	-2.346	-0.944	-1.765
O	-0.960	-2.298	-0.010
H	-1.715	-2.616	0.511
O	-1.687	-0.222	1.737
H	-2.489	0.320	1.793
H	1.552	1.988	1.775
H	-1.403	2.790	0.545
H	0.330	2.514	-1.756
H	2.972	0.968	-0.530
H	-1.667	-0.769	2.538
H	-0.654	-3.054	-0.535
H	-1.837	0.318	-2.518
H	0.633	-1.708	-2.548
O	1.042	-1.378	1.711
H	1.510	-1.100	2.515
H	0.973	-2.345	1.752

LanM-Y^{3+}

C	0.094	2.745	3.856
C	-1.062	1.745	3.736
C	-1.100	0.871	2.453
O	-0.262	1.192	1.538
O	-1.937	-0.045	2.418
H	1.060	2.228	3.868
H	-1.078	1.060	4.593
H	-2.023	2.277	3.768
C	-3.632	4.223	1.158
C	-2.720	4.290	-0.067
C	-2.017	2.946	-0.321
O	-0.794	2.994	-0.669
O	-2.684	1.896	-0.171
H	-4.201	5.152	1.298
H	-3.315	4.526	-0.961
H	-1.962	5.073	0.033
C	-4.378	0.073	-2.072
C	-4.598	-1.081	-1.083
O	-5.332	-2.030	-1.385
C	-3.231	-0.217	-3.075
C	-1.880	0.045	-2.428
O	-1.539	-0.680	-1.433
O	-1.169	1.012	-2.812
H	-5.319	0.160	-2.625
H	-3.291	-1.261	-3.406
H	-3.329	0.434	-3.949
N	-3.963	-0.990	0.119
C	-4.090	-2.015	1.132
C	-2.889	-2.928	1.438
O	-2.952	-3.666	2.424
H	-3.336	-0.209	0.302
H	-4.324	-1.556	2.097

H	-4.922	-2.661	0.839
N	-1.828	-2.909	0.584
C	-0.553	-3.470	1.016
C	0.455	-2.364	1.186
O	0.460	-1.355	0.493
H	-1.788	-2.176	-0.129
H	-0.734	-4.013	1.946
N	1.388	-2.588	2.148
C	2.572	-1.764	2.105
C	3.527	-2.308	1.027
O	3.998	-3.447	1.131
H	1.513	-3.543	2.455
H	2.273	-0.733	1.920
N	3.721	-1.480	-0.018
C	4.311	-1.932	-1.270
C	3.705	-1.193	-2.469
C	2.165	-1.391	-2.508
O	1.489	-0.373	-2.120
O	1.744	-2.496	-2.870
H	3.213	-0.588	-0.015
H	4.118	-3.005	-1.363
H	3.949	-0.126	-2.426
H	4.146	-1.612	-3.381
C	6.509	2.060	0.593
C	4.994	2.040	0.778
C	4.230	2.417	-0.494
C	2.763	1.981	-0.502
O	1.966	2.507	-1.313
O	2.412	1.036	0.286
H	6.870	3.050	0.280
H	4.678	1.039	1.083
H	4.703	2.714	1.595
H	4.275	3.492	-0.700
H	4.693	1.923	-1.362
Y	0.151	0.835	-0.709
H	6.817	1.343	-0.178
H	7.035	1.796	1.518
H	5.405	-1.799	-1.250
H	3.077	-1.825	3.075
H	-0.156	-4.165	0.263
H	-4.169	1.009	-1.547
H	-3.048	4.046	2.068
H	-4.331	3.390	1.053
H	0.011	3.341	4.776
H	0.107	3.420	2.997

LanM-La^{3+}

C	1.015	2.732	3.909
C	1.353	1.249	3.720
C	0.260	0.415	3.012
O	-0.206	0.921	1.927
O	-0.055	-0.684	3.505
H	0.778	3.183	2.942
H	2.247	1.179	3.086
H	1.576	0.769	4.680
C	-2.877	4.695	1.825
C	-2.402	4.686	0.370
C	-1.631	3.397	0.075
O	-0.406	3.489	-0.237
O	-2.247	2.300	0.186
H	-3.476	5.586	2.056
H	-3.268	4.730	-0.302
H	-1.757	5.544	0.156
C	-4.754	0.647	-1.056
C	-5.177	-0.721	-0.513
O	-6.271	-0.883	0.035
C	-3.909	0.677	-2.356
C	-2.404	0.570	-2.093
O	-2.007	-0.392	-1.338
O	-1.623	1.429	-2.557
H	-5.693	1.187	-1.214
H	-4.214	-0.136	-3.028
H	-4.080	1.619	-2.884
N	-4.265	-1.721	-0.670
C	-4.457	-3.028	-0.090
C	-3.257	-3.623	0.656
O	-3.321	-4.781	1.080
H	-3.348	-1.441	-1.048
H	-5.297	-2.964	0.609
H	-4.734	-3.781	-0.842
N	-2.162	-2.845	0.814
C	-0.989	-3.367	1.480
C	0.200	-2.482	1.273
O	0.217	-1.558	0.464
H	-2.097	-1.908	0.430
H	-1.174	-3.469	2.559
N	1.270	-2.886	1.984
C	2.488	-2.120	1.950
C	3.384	-2.637	0.806
O	4.272	-3.475	0.996
H	1.106	-3.496	2.769
H	2.260	-1.060	1.818
N	3.046	-2.126	-0.395
C	3.570	-2.595	-1.662
C	3.391	-1.526	-2.754
C	1.919	-1.069	-2.988
O	1.353	-0.555	-1.946
O	1.430	-1.225	-4.108
H	2.266	-1.472	-0.488
H	3.055	-3.520	-1.967
H	3.983	-0.643	-2.482
H	3.776	-1.918	-3.700
C	6.601	1.062	-0.367
C	5.143	1.080	0.067
C	4.309	2.067	-0.749
C	2.807	1.863	-0.569
O	2.031	2.328	-1.445
O	2.412	1.204	0.447
H	7.070	2.051	-0.266
H	4.713	0.081	-0.041
H	5.059	1.324	1.132
H	4.555	3.105	-0.483
H	4.521	1.973	-1.821
La	0.031	0.988	-0.491
H	6.695	0.768	-1.420
H	7.195	0.354	0.224
H	4.626	-2.853	-1.529
H	3.025	-2.259	2.889
H	-0.771	-4.373	1.100
H	-4.203	1.183	-0.273
H	-2.025	4.673	2.512
H	-3.482	3.805	2.021
H	1.856	3.281	4.354
H	0.145	2.865	4.564

LanM-Ce^{3+}

C	1.028	2.776	3.875
C	1.375	1.295	3.683
C	0.275	0.450	2.999
O	-0.189	0.930	1.901
O	-0.049	-0.633	3.521
H	0.772	3.223	2.910
H	2.257	1.231	3.033
H	1.620	0.820	4.640

C	-2.858	4.735	1.773
C	-2.255	4.737	0.367
C	-1.542	3.411	0.085
O	-0.310	3.449	-0.215
O	-2.208	2.345	0.181
H	-3.426	5.652	1.979
H	-3.054	4.857	-0.377
H	-1.543	5.558	0.237
C	-4.760	0.662	-1.056
C	-5.188	-0.707	-0.520
O	-6.291	-0.872	0.010
C	-3.895	0.697	-2.341
C	-2.393	0.589	-2.059
O	-2.008	-0.359	-1.281
O	-1.602	1.434	-2.533
H	-5.697	1.201	-1.226
H	-4.188	-0.115	-3.021
H	-4.058	1.639	-2.870
N	-4.272	-1.704	-0.663
C	-4.466	-3.010	-0.083
C	-3.273	-3.599	0.680
O	-3.342	-4.755	1.110
H	-3.349	-1.420	-1.023
H	-5.316	-2.949	0.605
H	-4.729	-3.766	-0.837
N	-2.180	-2.820	0.845
C	-1.013	-3.340	1.522
C	0.183	-2.468	1.304
O	0.205	-1.552	0.487
H	-2.109	-1.885	0.456
H	-1.200	-3.425	2.602
N	1.252	-2.871	2.018
C	2.471	-2.106	1.972
C	3.365	-2.640	0.834
O	4.254	-3.475	1.032
H	1.087	-3.467	2.813
H	2.245	-1.048	1.825
N	3.025	-2.145	-0.373
C	3.545	-2.631	-1.636
C	3.326	-1.589	-2.747
C	1.842	-1.157	-2.956
O	1.301	-0.606	-1.921
O	1.321	-1.368	-4.053
H	2.244	-1.493	-0.472
H	3.048	-3.574	-1.914
H	3.911	-0.691	-2.508
H	3.695	-1.997	-3.693
C	6.598	1.026	-0.390
C	5.129	1.034	0.010
C	4.307	2.026	-0.813
C	2.802	1.824	-0.644
O	2.032	2.248	-1.543
O	2.400	1.206	0.396
H	7.060	2.015	-0.266
H	4.707	0.033	-0.116
H	5.019	1.267	1.074
H	4.554	3.062	-0.539
H	4.529	1.935	-1.884
Ce	0.042	0.975	-0.490
H	6.718	0.744	-1.444
H	7.179	0.313	0.208
H	4.609	-2.862	-1.513
H	3.010	-2.234	2.912
H	-0.803	-4.354	1.158
H	-4.222	1.197	-0.263
H	-2.073	4.646	2.533
H	-3.524	3.875	1.887
H	1.871	3.333	4.305
H	0.167	2.903	4.544

LanM-Pr^{3+}

C	1.034	2.824	3.829
C	1.377	1.339	3.660
C	0.275	0.487	2.988
O	-0.188	0.950	1.883
O	-0.053	-0.586	3.528
H	0.780	3.256	2.857
H	2.259	1.262	3.011
H	1.620	0.878	4.624
C	-2.845	4.770	1.704
C	-2.159	4.761	0.337
C	-1.472	3.417	0.077
O	-0.234	3.426	-0.202
O	-2.164	2.366	0.159
H	-3.398	5.702	1.881
H	-2.908	4.914	-0.452
H	-1.416	5.560	0.255
C	-4.760	0.671	-1.078
C	-5.200	-0.688	-0.527
O	-6.307	-0.840	-0.004
C	-3.871	0.687	-2.348
C	-2.375	0.574	-2.039
O	-2.008	-0.360	-1.238
O	-1.569	1.403	-2.518
H	-5.693	1.210	-1.272
H	-4.157	-0.131	-3.024
H	-4.020	1.624	-2.890
N	-4.287	-1.692	-0.649
C	-4.486	-2.986	-0.043
C	-3.292	-3.568	0.722
O	-3.365	-4.717	1.170
H	-3.359	-1.415	-0.999
H	-5.331	-2.907	0.649
H	-4.760	-3.753	-0.781
N	-2.193	-2.794	0.869
C	-1.026	-3.313	1.547
C	0.171	-2.444	1.324
O	0.195	-1.534	0.501
H	-2.117	-1.864	0.468
H	-1.213	-3.392	2.627
N	1.240	-2.844	2.040
C	2.462	-2.085	1.983
C	3.353	-2.637	0.851
O	4.236	-3.477	1.058
H	1.077	-3.437	2.838
H	2.240	-1.028	1.822
N	3.017	-2.153	-0.361
C	3.535	-2.655	-1.618
C	3.285	-1.635	-2.745
C	1.793	-1.223	-2.936
O	1.274	-0.631	-1.913
O	1.247	-1.486	-4.010
H	2.241	-1.495	-0.465
H	3.051	-3.611	-1.874
H	3.864	-0.728	-2.533
H	3.643	-2.058	-3.689
C	6.599	1.006	-0.415
C	5.129	0.996	-0.018
C	4.300	1.995	-0.826
C	2.796	1.779	-0.663
O	2.026	2.194	-1.565
O	2.394	1.160	0.377
H	7.053	1.997	-0.273

H	4.715	-0.006	-0.161
H	5.016	1.211	1.050
H	4.538	3.028	-0.532
H	4.525	1.924	-1.897
Pr	0.048	0.957	-0.492
H	6.724	0.743	-1.473
H	7.185	0.289	0.173
H	4.604	-2.865	-1.503
H	3.001	-2.201	2.925
H	-0.817	-4.328	1.187
H	-4.234	1.216	-0.284
H	-2.110	4.656	2.509
H	-3.540	3.930	1.773
H	1.878	3.385	4.251
H	0.174	2.963	4.495

LanM-Nd^{3+}

C	1.037	2.877	3.793
C	1.382	1.390	3.658
C	0.285	0.529	2.987
O	-0.170	0.986	1.877
O	-0.045	-0.539	3.534
H	0.789	3.288	2.810
H	2.272	1.299	3.022
H	1.612	0.947	4.634
C	-2.861	4.781	1.664
C	-2.199	4.726	0.286
C	-1.510	3.376	0.061
O	-0.276	3.381	-0.232
O	-2.194	2.325	0.184
H	-3.416	5.716	1.817
H	-2.961	4.848	-0.495
H	-1.460	5.524	0.164
C	-4.764	0.643	-1.069
C	-5.188	-0.723	-0.521
O	-6.296	-0.890	-0.002
C	-3.872	0.670	-2.336
C	-2.374	0.577	-2.026
O	-1.995	-0.358	-1.229
O	-1.578	1.416	-2.501
H	-5.703	1.170	-1.266
H	-4.145	-0.150	-3.014
H	-4.031	1.606	-2.878
N	-4.261	-1.714	-0.637
C	-4.449	-3.013	-0.037
C	-3.255	-3.582	0.738
O	-3.317	-4.732	1.182
H	-3.335	-1.427	-0.988
H	-5.302	-2.947	0.645
H	-4.704	-3.782	-0.781
N	-2.169	-2.792	0.899
C	-0.998	-3.294	1.583
C	0.189	-2.414	1.351
O	0.197	-1.501	0.530
H	-2.104	-1.861	0.498
H	-1.183	-3.362	2.664
N	1.266	-2.804	2.058
C	2.485	-2.042	1.992
C	3.374	-2.603	0.863
O	4.255	-3.443	1.075
H	1.125	-3.432	2.832
H	2.257	-0.989	1.820
N	3.035	-2.128	-0.352
C	3.547	-2.643	-1.607
C	3.271	-1.644	-2.745
C	1.769	-1.258	-2.924
O	1.255	-0.650	-1.908
O	1.212	-1.556	-3.983
H	2.262	-1.468	-0.458
H	3.073	-3.610	-1.843
H	3.839	-0.725	-2.552
H	3.624	-2.075	-3.687
C	6.595	1.047	-0.453
C	5.130	1.026	-0.038
C	4.273	1.976	-0.875
C	2.774	1.748	-0.686
O	1.984	2.174	-1.567
O	2.392	1.110	0.349
H	7.032	2.050	-0.355
H	4.733	0.012	-0.137
H	5.025	1.281	1.022
H	4.498	3.024	-0.633
H	4.485	1.862	-1.945
Nd	0.041	0.941	-0.484
H	6.713	0.744	-1.501
H	7.201	0.363	0.155
H	4.620	-2.837	-1.501
H	3.025	-2.147	2.934
H	-0.782	-4.311	1.233
H	-4.247	1.193	-0.273
H	-2.113	4.698	2.460
H	-3.552	3.940	1.774
H	1.876	3.449	4.211
H	0.170	3.029	4.449

LanM-Pm^{3+}

C	1.071	2.912	3.738
C	1.405	1.419	3.642
C	0.304	0.542	2.999
O	-0.160	0.967	1.880
O	-0.020	-0.514	3.573
H	0.834	3.302	2.744
H	2.293	1.307	3.006
H	1.636	1.000	4.628
C	-2.814	4.832	1.599
C	-2.002	4.790	0.304
C	-1.367	3.413	0.080
O	-0.133	3.371	-0.215
O	-2.092	2.388	0.188
H	-3.335	5.790	1.725
H	-2.660	4.992	-0.552
H	-1.210	5.545	0.298
C	-4.778	0.680	-1.067
C	-5.209	-0.692	-0.538
O	-6.327	-0.865	-0.044
C	-3.865	0.720	-2.320
C	-2.371	0.621	-1.992
O	-2.010	-0.300	-1.170
O	-1.562	1.439	-2.478
H	-5.714	1.209	-1.271
H	-4.130	-0.092	-3.011
H	-4.016	1.661	-2.854
N	-4.277	-1.679	-0.643
C	-4.472	-2.982	-0.055
C	-3.292	-3.552	0.742
O	-3.361	-4.704	1.180
H	-3.346	-1.385	-0.971
H	-5.339	-2.925	0.610
H	-4.708	-3.747	-0.809
N	-2.210	-2.760	0.926
C	-1.048	-3.265	1.624
C	0.153	-2.409	1.374
O	0.170	-1.508	0.542
H	-2.138	-1.829	0.527

H	-1.234	-3.304	2.707
N	1.228	-2.804	2.081
C	2.451	-2.050	2.001
C	3.336	-2.633	0.879
O	4.222	-3.464	1.102
H	1.077	-3.400	2.879
H	2.230	-0.997	1.814
N	2.986	-2.183	-0.342
C	3.490	-2.714	-1.594
C	3.197	-1.728	-2.741
C	1.697	-1.325	-2.895
O	1.209	-0.700	-1.876
O	1.117	-1.625	-3.940
H	2.210	-1.526	-0.457
H	3.019	-3.685	-1.813
H	3.777	-0.811	-2.574
H	3.525	-2.175	-3.684
C	6.588	0.955	-0.505
C	5.113	0.934	-0.123
C	4.279	1.929	-0.931
C	2.777	1.718	-0.749
O	1.996	2.073	-1.666
O	2.384	1.161	0.331
H	7.034	1.947	-0.353
H	4.709	-0.072	-0.272
H	4.988	1.144	0.944
H	4.523	2.962	-0.642
H	4.494	1.853	-2.004
Pm	0.055	0.929	-0.467
H	6.726	0.698	-1.563
H	7.171	0.237	0.086
H	4.566	-2.902	-1.496
H	2.994	-2.143	2.943
H	-0.850	-4.293	1.298
H	-4.272	1.220	-0.257
H	-2.167	4.682	2.470
H	-3.551	4.025	1.597
H	1.913	3.485	4.147
H	0.202	3.087	4.385

LanM-Sm^{3+}

C	1.050	2.909	3.751
C	1.400	1.422	3.621
C	0.298	0.550	2.973
O	-0.157	0.978	1.850
O	-0.039	-0.501	3.547
H	0.796	3.316	2.768
H	2.281	1.331	2.972
H	1.645	0.987	4.596
C	-2.838	4.815	1.608
C	-2.045	4.765	0.302
C	-1.439	3.376	0.068
O	-0.212	3.320	-0.260
O	-2.172	2.365	0.206
H	-3.345	5.780	1.744
H	-2.711	4.981	-0.545
H	-1.241	5.507	0.287
C	-4.774	0.661	-1.077
C	-5.205	-0.706	-0.538
O	-6.323	-0.876	-0.042
C	-3.850	0.690	-2.322
C	-2.358	0.594	-1.984
O	-1.997	-0.323	-1.157
O	-1.547	1.410	-2.470
H	-5.708	1.186	-1.296
H	-4.109	-0.127	-3.008
H	-3.995	1.627	-2.865
N	-4.274	-1.694	-0.638
C	-4.467	-2.992	-0.038
C	-3.281	-3.558	0.752
O	-3.348	-4.707	1.200
H	-3.342	-1.403	-0.963
H	-5.328	-2.927	0.636
H	-4.713	-3.763	-0.783
N	-2.195	-2.769	0.919
C	-1.029	-3.272	1.611
C	0.167	-2.408	1.366
O	0.182	-1.506	0.535
H	-2.123	-1.839	0.515
H	-1.215	-3.322	2.693
N	1.241	-2.797	2.079
C	2.463	-2.040	2.001
C	3.350	-2.617	0.879
O	4.232	-3.454	1.100
H	1.095	-3.406	2.866
H	2.239	-0.988	1.815
N	3.008	-2.158	-0.341
C	3.514	-2.686	-1.593
C	3.222	-1.700	-2.740
C	1.720	-1.302	-2.899
O	1.226	-0.682	-1.880
O	1.146	-1.602	-3.948
H	2.233	-1.499	-0.455
H	3.045	-3.658	-1.814
H	3.798	-0.782	-2.570
H	3.555	-2.145	-3.682
C	6.589	0.995	-0.484
C	5.119	0.956	-0.086
C	4.268	1.941	-0.887
C	2.768	1.709	-0.712
O	1.985	2.090	-1.617
O	2.375	1.110	0.344
H	7.024	1.994	-0.337
H	4.725	-0.055	-0.231
H	5.003	1.164	0.983
H	4.495	2.977	-0.595
H	4.485	1.874	-1.960
Sm	0.058	0.925	-0.472
H	6.719	0.740	-1.543
H	7.189	0.286	0.101
H	4.590	-2.872	-1.494
H	3.004	-2.133	2.944
H	-0.824	-4.297	1.277
H	-4.276	1.211	-0.268
H	-2.181	4.654	2.469
H	-3.586	4.018	1.614
H	1.891	3.484	4.161
H	0.188	3.061	4.413

LanM-Eu^{3+}

C	1.070	2.934	3.719
C	1.406	1.441	3.631
C	0.311	0.564	2.979
O	-0.133	0.984	1.850
O	-0.030	-0.484	3.558
H	0.842	3.320	2.722
H	2.300	1.327	3.003
H	1.628	1.026	4.621
C	-2.812	4.845	1.567
C	-1.951	4.791	0.305
C	-1.357	3.396	0.076
O	-0.131	3.325	-0.248
O	-2.102	2.390	0.203
H	-3.307	5.818	1.686

H	-2.565	5.033	-0.573
H	-1.133	5.517	0.341
C	-4.775	0.680	-1.080
C	-5.214	-0.686	-0.544
O	-6.337	-0.854	-0.059
C	-3.840	0.708	-2.316
C	-2.351	0.610	-1.965
O	-2.002	-0.292	-1.117
O	-1.529	1.408	-2.462
H	-5.707	1.207	-1.307
H	-4.093	-0.109	-3.005
H	-3.978	1.645	-2.862
N	-4.282	-1.675	-0.632
C	-4.480	-2.971	-0.029
C	-3.298	-3.537	0.766
O	-3.367	-4.686	1.212
H	-3.347	-1.385	-0.947
H	-5.344	-2.903	0.641
H	-4.724	-3.743	-0.773
N	-2.213	-2.748	0.940
C	-1.049	-3.252	1.633
C	0.150	-2.393	1.384
O	0.164	-1.489	0.554
H	-2.140	-1.817	0.538
H	-1.233	-3.296	2.716
N	1.226	-2.787	2.090
C	2.451	-2.036	2.007
C	3.336	-2.628	0.890
O	4.213	-3.468	1.117
H	1.083	-3.402	2.874
H	2.231	-0.985	1.812
N	2.993	-2.179	-0.333
C	3.492	-2.718	-1.584
C	3.168	-1.749	-2.737
C	1.660	-1.364	-2.867
O	1.190	-0.715	-1.855
O	1.057	-1.702	-3.888
H	2.223	-1.515	-0.449
H	3.034	-3.700	-1.784
H	3.740	-0.824	-2.592
H	3.483	-2.205	-3.681
C	6.590	0.955	-0.510
C	5.114	0.913	-0.135
C	4.273	1.914	-0.929
C	2.770	1.688	-0.763
O	1.996	2.044	-1.683
O	2.371	1.119	0.309
H	7.026	1.949	-0.339
H	4.720	-0.094	-0.302
H	4.982	1.104	0.936
H	4.505	2.944	-0.620
H	4.496	1.860	-2.002
Eu	0.058	0.912	-0.469
H	6.736	0.718	-1.571
H	7.178	0.233	0.071
H	4.573	-2.887	-1.498
H	2.991	-2.123	2.952
H	-0.848	-4.279	1.304
H	-4.284	1.229	-0.266
H	-2.205	4.663	2.461
H	-3.574	4.062	1.527
H	1.908	3.510	4.134
H	0.195	3.111	4.358

LanM-Gd^{3+}

C	1.028	2.913	3.758
C	1.362	1.423	3.611
C	0.262	0.567	2.938
O	-0.167	1.006	1.809
O	-0.096	-0.482	3.504
H	0.786	3.335	2.779
H	2.251	1.332	2.973
H	1.591	0.974	4.584
C	-2.846	4.820	1.590
C	-1.958	4.779	0.348
C	-1.433	3.363	0.081
O	-0.207	3.254	-0.250
O	-2.221	2.395	0.192
H	-3.299	5.810	1.741
H	-2.535	5.089	-0.534
H	-1.106	5.461	0.434
C	-4.767	0.665	-1.104
C	-5.213	-0.695	-0.560
O	-6.338	-0.855	-0.076
C	-3.810	0.682	-2.324
C	-2.328	0.577	-1.950
O	-1.987	-0.331	-1.110
O	-1.498	1.383	-2.429
H	-5.695	1.188	-1.354
H	-4.057	-0.136	-3.014
H	-3.935	1.618	-2.875
N	-4.286	-1.689	-0.640
C	-4.487	-2.976	-0.019
C	-3.297	-3.543	0.764
O	-3.367	-4.689	1.217
H	-3.347	-1.405	-0.949
H	-5.341	-2.894	0.662
H	-4.749	-3.754	-0.751
N	-2.207	-2.758	0.921
C	-1.042	-3.268	1.609
C	0.156	-2.403	1.375
O	0.180	-1.503	0.543
H	-2.132	-1.828	0.519
H	-1.230	-3.331	2.690
N	1.226	-2.794	2.095
C	2.448	-2.037	2.020
C	3.342	-2.618	0.904
O	4.221	-3.456	1.131
H	1.074	-3.398	2.885
H	2.226	-0.986	1.830
N	3.009	-2.159	-0.318
C	3.522	-2.687	-1.567
C	3.218	-1.709	-2.719
C	1.715	-1.316	-2.879
O	1.218	-0.699	-1.860
O	1.144	-1.615	-3.930
H	2.235	-1.499	-0.434
H	3.064	-3.665	-1.784
H	3.790	-0.786	-2.555
H	3.553	-2.156	-3.660
C	6.592	0.994	-0.441
C	5.118	0.951	-0.056
C	4.266	1.916	-0.880
C	2.767	1.669	-0.719
O	1.985	2.059	-1.621
O	2.372	1.048	0.323
H	7.019	1.997	-0.308
H	4.733	-0.064	-0.188
H	4.992	1.176	1.009
H	4.478	2.959	-0.603
H	4.495	1.835	-1.950
Gd	0.076	0.910	-0.492
H	6.733	0.721	-1.495

H	7.190	0.298	0.160
H	4.600	-2.861	-1.468
H	2.985	-2.129	2.966
H	-0.835	-4.289	1.265
H	-4.292	1.225	-0.289
H	-2.272	4.570	2.489
H	-3.641	4.076	1.496
H	1.872	3.474	4.181
H	0.163	3.066	4.415

LanM-Tb^{3+}

C	1.007	2.897	3.804
C	1.357	1.422	3.563
C	0.238	0.583	2.898
O	-0.179	1.019	1.761
O	-0.148	-0.450	3.473
H	0.718	3.367	2.859
H	2.218	1.377	2.885
H	1.629	0.931	4.504
C	-2.879	4.788	1.641
C	-2.012	4.769	0.385
C	-1.561	3.340	0.056
O	-0.349	3.194	-0.316
O	-2.390	2.411	0.170
H	-3.287	5.786	1.849
H	-2.588	5.144	-0.473
H	-1.129	5.407	0.486
C	-4.761	0.637	-1.085
C	-5.196	-0.726	-0.541
O	-6.317	-0.896	-0.053
C	-3.809	0.657	-2.308
C	-2.323	0.554	-1.951
O	-1.963	-0.349	-1.109
O	-1.499	1.351	-2.452
H	-5.693	1.152	-1.336
H	-4.058	-0.159	-3.000
H	-3.937	1.594	-2.855
N	-4.263	-1.715	-0.626
C	-4.458	-3.006	-0.012
C	-3.262	-3.571	0.763
O	-3.320	-4.723	1.204
H	-3.327	-1.427	-0.939
H	-5.308	-2.929	0.674
H	-4.720	-3.782	-0.745
N	-2.179	-2.778	0.927
C	-1.009	-3.285	1.608
C	0.182	-2.410	1.378
O	0.206	-1.513	0.542
H	-2.114	-1.842	0.536
H	-1.194	-3.362	2.688
N	1.248	-2.784	2.111
C	2.470	-2.029	2.034
C	3.360	-2.606	0.912
O	4.223	-3.463	1.130
H	1.107	-3.414	2.883
H	2.247	-0.977	1.848
N	3.044	-2.121	-0.305
C	3.556	-2.645	-1.556
C	3.254	-1.665	-2.706
C	1.745	-1.303	-2.879
O	1.238	-0.660	-1.881
O	1.177	-1.651	-3.916
H	2.280	-1.451	-0.411
H	3.098	-3.623	-1.775
H	3.810	-0.736	-2.531
H	3.605	-2.105	-3.644
C	6.594	1.052	-0.399
C	5.129	1.029	0.015
C	4.266	1.949	-0.848
C	2.770	1.688	-0.687
O	1.982	2.122	-1.565
O	2.382	1.013	0.322
H	7.022	2.062	-0.328
H	4.740	0.009	-0.059
H	5.021	1.309	1.069
H	4.462	3.006	-0.619
H	4.500	1.826	-1.913
Tb	0.079	0.901	-0.515
H	6.715	0.723	-1.438
H	7.205	0.391	0.228
H	4.634	-2.819	-1.457
H	3.011	-2.124	2.977
H	-0.796	-4.301	1.252
H	-4.288	1.203	-0.274
H	-2.301	4.471	2.516
H	-3.704	4.082	1.525
H	1.860	3.448	4.220
H	0.168	3.001	4.503

LanM-Dy^{3+}

C	0.995	2.917	3.809
C	1.346	1.452	3.509
C	0.206	0.623	2.865
O	-0.204	1.044	1.719
O	-0.205	-0.384	3.466
H	0.675	3.418	2.891
H	2.183	1.433	2.801
H	1.653	0.938	4.427
C	-2.887	4.794	1.627
C	-1.916	4.789	0.450
C	-1.601	3.347	0.028
O	-0.396	3.117	-0.336
O	-2.519	2.508	0.071
H	-3.200	5.811	1.903
H	-2.365	5.304	-0.412
H	-0.982	5.309	0.683
C	-4.756	0.629	-1.087
C	-5.194	-0.733	-0.546
O	-6.320	-0.908	-0.070
C	-3.789	0.647	-2.296
C	-2.306	0.537	-1.930
O	-1.959	-0.328	-1.043
O	-1.465	1.286	-2.475
H	-5.686	1.142	-1.351
H	-4.033	-0.166	-2.995
H	-3.905	1.586	-2.843
N	-4.257	-1.721	-0.617
C	-4.451	-3.009	0.003
C	-3.257	-3.567	0.785
O	-3.315	-4.716	1.235
H	-3.318	-1.428	-0.912
H	-5.306	-2.932	0.683
H	-4.707	-3.789	-0.728
N	-2.173	-2.774	0.945
C	-1.006	-3.277	1.634
C	0.185	-2.401	1.404
O	0.213	-1.504	0.567
H	-2.108	-1.837	0.555
H	-1.195	-3.347	2.714
N	1.246	-2.761	2.151
C	2.470	-2.015	2.064
C	3.357	-2.600	0.945
O	4.201	-3.475	1.165
H	1.127	-3.443	2.881

H	2.246	-0.964	1.872
N	3.060	-2.104	-0.272
C	3.566	-2.644	-1.520
C	3.246	-1.691	-2.685
C	1.729	-1.367	-2.852
O	1.229	-0.676	-1.883
O	1.148	-1.788	-3.854
H	2.300	-1.430	-0.380
H	3.116	-3.630	-1.716
H	3.787	-0.749	-2.531
H	3.600	-2.145	-3.616
C	6.596	1.062	-0.369
C	5.130	1.067	0.041
C	4.270	1.932	-0.880
C	2.773	1.684	-0.719
O	1.987	2.121	-1.596
O	2.379	1.011	0.291
H	7.027	2.073	-0.361
H	4.739	0.046	0.029
H	5.021	1.413	1.076
H	4.465	3.002	-0.720
H	4.511	1.741	-1.934
Dy	0.098	0.887	-0.536
H	6.719	0.668	-1.386
H	7.205	0.440	0.299
H	4.647	-2.807	-1.428
H	3.012	-2.102	3.008
H	-0.792	-4.295	1.285
H	-4.294	1.199	-0.273
H	-2.429	4.331	2.508
H	-3.769	4.203	1.374
H	1.859	3.459	4.216
H	0.178	2.993	4.538

LanM-Ho^{3+}

C	0.988	2.989	3.751
C	1.339	1.512	3.519
C	0.214	0.662	2.874
O	-0.185	1.060	1.719
O	-0.187	-0.344	3.485
H	0.687	3.450	2.806
H	2.195	1.464	2.834
H	1.622	1.032	4.463
C	-2.898	4.828	1.545
C	-1.908	4.779	0.386
C	-1.593	3.323	0.013
O	-0.389	3.080	-0.338
O	-2.517	2.488	0.079
H	-3.213	5.854	1.780
H	-2.338	5.270	-0.500
H	-0.974	5.300	0.618
C	-4.759	0.623	-1.112
C	-5.199	-0.734	-0.558
O	-6.331	-0.904	-0.094
C	-3.764	0.636	-2.300
C	-2.290	0.529	-1.903
O	-1.959	-0.331	-1.006
O	-1.435	1.270	-2.438
H	-5.686	1.125	-1.404
H	-3.991	-0.179	-3.001
H	-3.870	1.573	-2.852
N	-4.260	-1.720	-0.603
C	-4.455	-2.995	0.043
C	-3.253	-3.545	0.820
O	-3.300	-4.694	1.269
H	-3.318	-1.430	-0.890
H	-5.301	-2.902	0.732
H	-4.724	-3.788	-0.670
N	-2.176	-2.742	0.979
C	-1.001	-3.238	1.661
C	0.185	-2.357	1.424
O	0.209	-1.468	0.578
H	-2.121	-1.805	0.588
H	-1.183	-3.310	2.742
N	1.249	-2.710	2.169
C	2.472	-1.963	2.074
C	3.362	-2.564	0.965
O	4.209	-3.433	1.197
H	1.127	-3.374	2.915
H	2.250	-0.916	1.866
N	3.062	-2.089	-0.260
C	3.570	-2.639	-1.501
C	3.225	-1.710	-2.680
C	1.704	-1.411	-2.839
O	1.201	-0.717	-1.874
O	1.120	-1.849	-3.832
H	2.304	-1.415	-0.375
H	3.138	-3.636	-1.680
H	3.753	-0.758	-2.546
H	3.579	-2.174	-3.606
C	6.593	1.088	-0.402
C	5.123	1.056	0.001
C	4.244	1.919	-0.906
C	2.751	1.648	-0.734
O	1.951	2.064	-1.610
O	2.372	0.978	0.283
H	7.002	2.108	-0.371
H	4.755	0.026	-0.031
H	5.003	1.380	1.041
H	4.425	2.989	-0.732
H	4.481	1.746	-1.964
Ho	0.105	0.842	-0.534
H	6.730	0.717	-1.425
H	7.209	0.466	0.258
H	4.654	-2.780	-1.416
H	3.013	-2.038	3.020
H	-0.783	-4.255	1.309
H	-4.319	1.207	-0.296
H	-2.457	4.395	2.449
H	-3.778	4.230	1.297
H	1.844	3.546	4.154
H	0.156	3.096	4.459

LanM-Er^{3+}

C	0.988	2.999	3.740
C	1.323	1.514	3.531
C	0.211	0.660	2.867
O	-0.183	1.063	1.712
O	-0.183	-0.357	3.465
H	0.708	3.452	2.785
H	2.193	1.449	2.866
H	1.581	1.040	4.485
C	-2.902	4.830	1.534
C	-1.876	4.778	0.407
C	-1.616	3.320	-0.004
O	-0.416	3.036	-0.341
O	-2.580	2.532	0.022
H	-3.177	5.861	1.799
H	-2.255	5.316	-0.474
H	-0.928	5.249	0.688
C	-4.763	0.618	-1.112
C	-5.203	-0.740	-0.561
O	-6.337	-0.914	-0.103
C	-3.756	0.634	-2.289

C	-2.284	0.524	-1.883
O	-1.962	-0.318	-0.964
O	-1.419	1.240	-2.433
H	-5.689	1.116	-1.414
H	-3.976	-0.180	-2.994
H	-3.856	1.571	-2.841
N	-4.261	-1.725	-0.601
C	-4.455	-2.997	0.051
C	-3.249	-3.546	0.822
O	-3.288	-4.700	1.260
H	-3.317	-1.429	-0.873
H	-5.297	-2.899	0.745
H	-4.730	-3.792	-0.657
N	-2.178	-2.737	0.991
C	-0.999	-3.234	1.664
C	0.188	-2.353	1.424
O	0.214	-1.469	0.573
H	-2.128	-1.797	0.605
H	-1.174	-3.308	2.747
N	1.253	-2.708	2.165
C	2.474	-1.956	2.071
C	3.368	-2.555	0.965
O	4.228	-3.409	1.202
H	1.121	-3.344	2.934
H	2.250	-0.908	1.862
N	3.057	-2.091	-0.262
C	3.568	-2.638	-1.504
C	3.224	-1.702	-2.678
C	1.702	-1.400	-2.836
O	1.197	-0.718	-1.863
O	1.118	-1.830	-3.831
H	2.288	-1.430	-0.379
H	3.137	-3.635	-1.688
H	3.751	-0.750	-2.538
H	3.577	-2.160	-3.607
C	6.589	1.094	-0.415
C	5.118	1.023	-0.025
C	4.236	1.939	-0.877
C	2.745	1.651	-0.718
O	1.949	2.045	-1.607
O	2.364	0.990	0.304
H	6.990	2.112	-0.313
H	4.759	-0.005	-0.125
H	4.988	1.277	1.033
H	4.413	2.996	-0.629
H	4.473	1.837	-1.943
Er	0.112	0.827	-0.529
H	6.736	0.794	-1.460
H	7.207	0.434	0.205
H	4.653	-2.778	-1.417
H	3.015	-2.029	3.017
H	-0.784	-4.250	1.309
H	-4.334	1.205	-0.293
H	-2.514	4.342	2.435
H	-3.799	4.285	1.233
H	1.846	3.548	4.149
H	0.147	3.127	4.434

LanM-Tm^{3+}

C	1.042	2.964	3.721
C	1.325	1.462	3.609
C	0.220	0.647	2.894
O	-0.121	1.095	1.738
O	-0.225	-0.368	3.453
H	0.838	3.377	2.729
H	2.240	1.325	3.018
H	1.489	1.017	4.597
C	-2.963	4.690	1.639
C	-2.002	4.799	0.452
C	-2.012	3.463	-0.316
O	-0.895	2.839	-0.335
O	-3.097	3.097	-0.798
H	-3.045	5.632	2.199
H	-2.339	5.597	-0.225
H	-0.986	5.034	0.782
C	-4.749	0.451	-1.015
C	-5.134	-0.924	-0.470
O	-6.257	-1.156	-0.012
C	-3.762	0.504	-2.201
C	-2.294	0.459	-1.784
O	-2.005	-0.138	-0.673
O	-1.405	0.990	-2.480
H	-5.695	0.922	-1.298
H	-3.950	-0.314	-2.910
H	-3.891	1.444	-2.739
N	-4.153	-1.872	-0.522
C	-4.294	-3.160	0.113
C	-3.044	-3.687	0.823
O	-3.009	-4.865	1.193
H	-3.224	-1.531	-0.767
H	-5.112	-3.095	0.840
H	-4.576	-3.948	-0.598
N	-2.015	-2.830	1.017
C	-0.788	-3.306	1.613
C	0.342	-2.346	1.384
O	0.303	-1.434	0.565
H	-2.030	-1.862	0.703
H	-0.913	-3.462	2.693
N	1.427	-2.645	2.122
C	2.650	-1.916	1.949
C	3.450	-2.557	0.793
O	4.055	-3.623	0.945
H	1.409	-3.448	2.730
H	2.425	-0.868	1.750
N	3.337	-1.902	-0.382
C	3.674	-2.516	-1.662
C	3.095	-1.691	-2.822
C	1.559	-1.489	-2.677
O	1.244	-0.497	-1.918
O	0.807	-2.285	-3.241
H	2.748	-1.070	-0.416
H	3.265	-3.534	-1.687
H	3.592	-0.714	-2.860
H	3.299	-2.224	-3.757
C	6.595	1.303	-0.600
C	5.193	1.388	-0.003
C	4.186	2.029	-0.958
C	2.722	1.768	-0.605
O	1.831	2.356	-1.272
O	2.449	0.915	0.298
H	6.986	2.294	-0.868
H	4.838	0.384	0.245
H	5.216	1.945	0.943
H	4.335	3.112	-1.050
H	4.320	1.625	-1.971
Tm	0.096	0.855	-0.485
H	6.593	0.697	-1.515
H	7.308	0.845	0.097
H	4.765	-2.612	-1.759
H	3.243	-1.992	2.864
H	-0.533	-4.282	1.179
H	-4.330	1.054	-0.205
H	-2.625	3.913	2.333

H	-3.955	4.407	1.277
H	1.892	3.504	4.159
H	0.163	3.159	4.349

LanM-Yb^{3+}

C	1.062	3.014	3.711
C	1.274	1.497	3.650
C	0.165	0.734	2.884
O	-0.058	1.169	1.694
O	-0.396	-0.221	3.441
H	0.946	3.412	2.699
H	2.218	1.298	3.125
H	1.349	1.068	4.656
C	-2.942	4.727	1.617
C	-1.915	4.783	0.484
C	-1.959	3.459	-0.306
O	-0.843	2.840	-0.399
O	-3.064	3.104	-0.750
H	-3.006	5.674	2.170
H	-2.166	5.604	-0.202
H	-0.904	4.956	0.866
C	-4.732	0.468	-1.003
C	-5.108	-0.915	-0.474
O	-6.236	-1.167	-0.038
C	-3.768	0.537	-2.206
C	-2.292	0.488	-1.820
O	-1.990	-0.095	-0.702
O	-1.417	0.999	-2.542
H	-5.684	0.943	-1.261
H	-3.971	-0.270	-2.924
H	-3.908	1.485	-2.727
N	-4.112	-1.847	-0.513
C	-4.252	-3.149	0.093
C	-3.010	-3.673	0.819
O	-2.964	-4.856	1.168
H	-3.185	-1.494	-0.754
H	-5.085	-3.106	0.804
H	-4.509	-3.926	-0.639
N	-2.000	-2.803	1.051
C	-0.773	-3.271	1.652
C	0.346	-2.289	1.450
O	0.295	-1.362	0.652
H	-2.023	-1.833	0.749
H	-0.904	-3.446	2.729
N	1.426	-2.568	2.217
C	2.666	-1.881	1.976
C	3.404	-2.581	0.817
O	3.863	-3.720	0.957
H	1.479	-3.481	2.644
H	2.456	-0.835	1.755
N	3.405	-1.888	-0.341
C	3.688	-2.510	-1.630
C	2.988	-1.745	-2.762
C	1.457	-1.668	-2.503
O	1.076	-0.577	-1.944
O	0.774	-2.651	-2.804
H	2.936	-0.979	-0.343
H	3.322	-3.542	-1.600
H	3.409	-0.737	-2.851
H	3.169	-2.283	-3.699
C	6.612	1.315	-0.600
C	5.200	1.475	-0.042
C	4.202	1.973	-1.090
C	2.736	1.710	-0.745
O	1.841	2.299	-1.401
O	2.466	0.847	0.155
H	7.004	2.260	-0.999
H	4.845	0.513	0.337
H	5.207	2.158	0.817
H	4.324	3.041	-1.306
H	4.376	1.454	-2.044
Yb	0.108	0.841	-0.506
H	6.628	0.585	-1.418
H	7.315	0.965	0.166
H	4.774	-2.557	-1.804
H	3.285	-1.943	2.876
H	-0.499	-4.235	1.204
H	-4.296	1.059	-0.192
H	-2.683	3.937	2.331
H	-3.925	4.490	1.200
H	1.906	3.523	4.195
H	0.154	3.266	4.272

LanM-Lu^{3+}

C	-0.983	-3.065	3.671
C	-0.233	-1.777	4.033
C	0.265	-0.881	2.861
O	-0.065	-1.261	1.683
O	0.925	0.123	3.174
H	-1.863	-2.841	3.062
H	-0.865	-1.134	4.661
H	0.648	-2.000	4.648
C	2.972	-4.724	1.444
C	1.903	-4.766	0.349
C	1.916	-3.434	-0.428
O	0.796	-2.815	-0.469
O	3.003	-3.072	-0.909
H	3.058	-5.679	1.980
H	2.130	-5.580	-0.354
H	0.907	-4.945	0.765
C	4.672	-0.414	-1.153
C	5.069	0.955	-0.608
O	6.224	1.218	-0.259
C	3.648	-0.453	-2.306
C	2.194	-0.439	-1.844
O	1.938	0.084	-0.688
O	1.289	-0.924	-2.549
H	5.611	-0.876	-1.474
H	3.803	0.382	-3.004
H	3.771	-1.379	-2.869
N	4.056	1.862	-0.529
C	4.227	3.153	0.089
C	2.983	3.685	0.798
O	2.898	4.885	1.074
H	3.118	1.503	-0.715
H	5.054	3.090	0.807
H	4.506	3.933	-0.633
N	2.007	2.795	1.098
C	0.768	3.262	1.668
C	-0.341	2.258	1.497
O	-0.316	1.351	0.677
H	2.078	1.801	0.903
H	0.883	3.491	2.737
N	-1.394	2.500	2.324
C	-2.655	1.849	2.060
C	-3.380	2.594	0.923
O	-3.755	3.761	1.079
H	-1.448	3.424	2.730
H	-2.467	0.803	1.820
N	-3.479	1.895	-0.227
C	-3.775	2.532	-1.506
C	-3.078	1.789	-2.654
C	-1.544	1.725	-2.407
O	-1.139	0.610	-1.915

O	-0.881	2.735	-2.655
H	-3.058	0.963	-0.242
H	-3.416	3.565	-1.460
H	-3.490	0.779	-2.758
H	-3.271	2.340	-3.582
C	-6.654	-1.323	-0.461
C	-5.235	-1.552	0.054
C	-4.259	-1.970	-1.048
C	-2.792	-1.687	-0.724
O	-1.895	-2.263	-1.389
O	-2.524	-0.817	0.167
H	-7.068	-2.223	-0.936
H	-4.860	-0.632	0.509
H	-5.239	-2.307	0.852
H	-4.362	-3.026	-1.321
H	-4.471	-1.401	-1.966
Lu	-0.166	-0.825	-0.520
H	-6.671	-0.523	-1.211
H	-7.338	-1.033	0.346
H	-4.863	2.574	-1.674
H	-3.271	1.906	2.964
H	0.482	4.198	1.172
H	4.280	-1.030	-0.339
H	2.741	-3.945	2.179
H	3.938	-4.480	0.993
H	-1.305	-3.604	4.574
H	-0.351	-3.732	3.078

LanM-Ac^{3+}

C	-0.878	-2.432	4.124
C	0.191	-1.350	4.331
C	0.277	-0.306	3.192
O	0.290	-0.791	2.007
O	0.359	0.900	3.503
H	-1.883	-1.995	4.099
H	0.032	-0.811	5.272
H	1.179	-1.827	4.396
C	2.929	-4.474	1.960
C	2.509	-4.602	0.492
C	1.697	-3.376	0.074
O	0.487	-3.534	-0.269
O	2.265	-2.246	0.134
H	3.542	-5.325	2.284
H	3.401	-4.661	-0.144
H	1.911	-5.504	0.328
C	4.672	-0.543	-1.159
C	5.110	0.829	-0.639
O	6.200	0.988	-0.082
C	3.858	-0.584	-2.477
C	2.351	-0.472	-2.236
O	1.947	0.512	-1.512
O	1.576	-1.354	-2.670
H	5.604	-1.103	-1.285
H	4.178	0.225	-3.148
H	4.040	-1.530	-2.993
N	4.215	1.841	-0.831
C	4.427	3.161	-0.290
C	3.261	3.777	0.490
O	3.364	4.925	0.931
H	3.299	1.562	-1.211
H	5.292	3.112	0.378
H	4.676	3.893	-1.072
N	2.145	3.030	0.656
C	1.007	3.566	1.370
C	-0.187	2.671	1.240
O	-0.238	1.722	0.459
H	2.041	2.104	0.254
H	1.249	3.690	2.434
N	-1.222	3.094	1.987
C	-2.444	2.336	2.036
C	-3.392	2.791	0.909
O	-4.297	3.608	1.109
H	-1.040	3.770	2.710
H	-2.212	1.269	1.960
N	-3.086	2.251	-0.288
C	-3.678	2.664	-1.544
C	-3.649	1.534	-2.585
C	-2.231	0.998	-2.922
O	-1.557	0.600	-1.894
O	-1.868	0.989	-4.103
H	-2.290	1.622	-0.396
H	-3.141	3.539	-1.944
H	-4.249	0.693	-2.214
H	-4.108	1.894	-3.511
C	-6.643	-0.941	0.024
C	-5.160	-1.022	0.355
C	-4.437	-2.097	-0.459
C	-2.919	-1.955	-0.378
O	-2.214	-2.384	-1.332
O	-2.437	-1.366	0.644
H	-7.156	-1.894	0.214
H	-4.690	-0.053	0.162
H	-5.014	-1.212	1.423
H	-4.716	-3.102	-0.110
H	-4.719	-2.051	-1.518
Ac	-0.089	-0.938	-0.478
H	-6.799	-0.698	-1.035
H	-7.151	-0.170	0.615
H	-4.705	2.989	-1.345
H	-2.944	2.525	2.988
H	0.762	4.562	0.980
H	4.101	-1.052	-0.373
H	2.051	-4.423	2.612
H	3.503	-3.555	2.105
H	-0.853	-3.177	4.931
H	-0.725	-2.938	3.168

LanM-Th^{3+}

C	-0.968	-3.147	3.482
C	-0.329	-1.759	3.623
C	0.866	-1.636	2.665
O	0.677	-0.863	1.639
O	1.892	-2.279	2.903
H	-1.323	-3.305	2.460
H	-1.060	-0.975	3.411
H	0.041	-1.625	4.648
C	2.713	-4.888	0.880
C	1.305	-4.839	0.272
C	0.826	-3.437	-0.099
O	-0.386	-3.125	0.129
O	1.611	-2.613	-0.656
H	2.990	-5.919	1.135
H	1.268	-5.439	-0.650
H	0.564	-5.263	0.956
C	4.632	-0.494	-1.405
C	5.197	0.735	-0.687
O	6.365	0.754	-0.291
C	3.691	-0.274	-2.619
C	2.231	-0.228	-2.200
O	1.963	0.446	-1.135
O	1.334	-0.865	-2.801
H	5.514	-1.054	-1.728
H	3.938	0.663	-3.134
H	3.806	-1.084	-3.343

N	4.329	1.764	-0.502
C	4.653	2.906	0.325
C	3.510	3.439	1.200
O	3.654	4.540	1.742
H	3.364	1.603	-0.788
H	5.497	2.630	0.965
H	4.984	3.766	-0.273
N	2.405	2.678	1.327
C	1.200	3.186	1.970
C	0.039	2.254	1.770
O	-0.205	1.855	0.529
H	2.302	1.795	0.844
H	1.397	3.351	3.040
N	-1.089	2.791	2.478
C	-2.302	2.016	2.433
C	-3.315	2.447	1.360
O	-4.536	2.359	1.565
H	-0.843	3.102	3.409
H	-2.102	0.948	2.255
N	-2.759	2.882	0.208
C	-3.545	3.126	-0.983
C	-3.387	2.008	-2.033
C	-1.932	1.884	-2.539
O	-1.263	0.865	-2.087
O	-1.505	2.740	-3.314
H	-1.746	2.701	0.117
H	-3.235	4.075	-1.441
H	-3.730	1.065	-1.604
H	-4.017	2.254	-2.898
C	-6.679	-0.574	-0.129
C	-5.233	-0.881	0.266
C	-4.437	-1.556	-0.858
C	-2.923	-1.444	-0.722
O	-2.180	-1.857	-1.659
O	-2.431	-0.879	0.314
H	-7.237	-1.480	-0.404
H	-4.746	0.053	0.556
H	-5.212	-1.516	1.160
H	-4.689	-2.620	-0.957
H	-4.680	-1.107	-1.830
Th	-0.155	-0.684	-0.605
H	-6.711	0.106	-0.989
H	-7.214	-0.086	0.693
H	-4.589	3.210	-0.669
H	-2.842	2.091	3.383
H	0.984	4.185	1.543
H	4.117	-1.115	-0.662
H	2.752	-4.266	1.778
H	3.447	-4.495	0.171
H	-1.815	-3.271	4.170
H	-0.226	-3.922	3.706

LanM-Pa^{3+}

C	-1.173	-2.664	3.813
C	-1.543	-1.187	3.637
C	-0.392	-0.336	3.074
O	0.015	-0.678	1.887
O	0.068	0.592	3.748
H	-0.892	-3.101	2.851
H	-2.382	-1.117	2.935
H	-1.846	-0.746	4.593
C	2.588	-4.858	1.718
C	1.801	-4.740	0.414
C	1.215	-3.343	0.240
O	-0.020	-3.247	-0.065
O	1.955	-2.335	0.386
H	3.074	-5.837	1.812
H	2.465	-4.923	-0.441
H	0.988	-5.469	0.364
C	4.806	-0.884	-1.021
C	5.318	0.489	-0.571
O	6.453	0.614	-0.101
C	3.867	-0.948	-2.255
C	2.386	-0.757	-1.925
O	2.059	0.233	-1.177
O	1.525	-1.569	-2.343
H	5.708	-1.471	-1.217
H	4.158	-0.186	-2.991
H	3.965	-1.921	-2.743
N	4.443	1.520	-0.714
C	4.703	2.835	-0.171
C	3.600	3.459	0.700
O	3.823	4.558	1.220
H	3.497	1.272	-1.017
H	5.612	2.772	0.433
H	4.903	3.568	-0.965
N	2.437	2.789	0.829
C	1.321	3.331	1.589
C	0.051	2.661	1.247
O	-0.067	1.854	0.253
H	2.261	1.910	0.357
H	1.528	3.223	2.673
N	-0.964	2.981	2.160
C	-2.246	2.339	1.981
C	-3.128	2.820	0.819
O	-4.328	3.068	0.997
H	-0.635	2.774	3.108
H	-2.157	1.249	1.852
N	-2.513	2.860	-0.386
C	-3.240	2.999	-1.627
C	-3.230	1.705	-2.474
C	-1.807	1.255	-2.875
O	-1.207	0.514	-1.989
O	-1.331	1.635	-3.943
H	-1.564	2.466	-0.422
H	-2.802	3.809	-2.227
H	-3.727	0.912	-1.908
H	-3.793	1.892	-3.395
C	-6.556	-0.453	-0.476
C	-5.184	-0.802	0.085
C	-4.407	-1.756	-0.829
C	-2.897	-1.672	-0.648
O	-2.134	-2.137	-1.528
O	-2.451	-1.067	0.392
H	-7.183	-1.344	-0.619
H	-4.610	0.118	0.221
H	-5.278	-1.239	1.086
H	-4.720	-2.799	-0.682
H	-4.603	-1.534	-1.886
Pa	-0.150	-0.801	-0.419
H	-6.464	0.039	-1.451
H	-7.098	0.233	0.186
H	-4.266	3.278	-1.372
H	-2.840	2.512	2.882
H	1.286	4.420	1.416
H	4.296	-1.354	-0.170
H	1.933	-4.721	2.585
H	3.356	-4.080	1.761
H	-2.016	-3.238	4.218
H	-0.327	-2.783	4.502

LanM-U^{3+}

C	-1.025	-2.615	3.951
C	-1.373	-1.136	3.762

C	-0.280	-0.299	3.060
O	0.203	-0.808	1.980
O	0.028	0.802	3.550
H	-0.788	-3.067	2.984
H	-2.264	-1.070	3.124
H	-1.601	-0.656	4.720
C	2.872	-4.601	1.895
C	2.373	-4.595	0.448
C	1.603	-3.308	0.144
O	0.399	-3.398	-0.240
O	2.194	-2.202	0.308
H	3.473	-5.493	2.117
H	3.230	-4.645	-0.237
H	1.725	-5.452	0.244
C	4.755	-0.584	-1.025
C	5.181	0.783	-0.482
O	6.273	0.941	0.071
C	3.903	-0.608	-2.320
C	2.403	-0.517	-2.033
O	2.002	0.462	-1.302
O	1.628	-1.412	-2.440
H	5.692	-1.125	-1.186
H	4.195	0.216	-2.984
H	4.078	-1.543	-2.859
N	4.276	1.787	-0.648
C	4.468	3.094	-0.068
C	3.265	3.694	0.667
O	3.335	4.849	1.099
H	3.358	1.511	-1.027
H	5.303	3.028	0.638
H	4.755	3.845	-0.819
N	2.161	2.926	0.808
C	0.986	3.457	1.462
C	-0.201	2.569	1.266
O	-0.212	1.630	0.472
H	2.088	1.992	0.416
H	1.170	3.577	2.539
N	-1.274	2.982	1.968
C	-2.492	2.216	1.938
C	-3.387	2.723	0.789
O	-4.278	3.560	0.968
H	-1.114	3.602	2.745
H	-2.266	1.155	1.813
N	-3.044	2.203	-0.406
C	-3.567	2.653	-1.681
C	-3.348	1.578	-2.760
C	-1.864	1.140	-2.954
O	-1.327	0.610	-1.906
O	-1.338	1.332	-4.053
H	-2.266	1.544	-0.489
H	-3.070	3.588	-1.988
H	-3.935	0.689	-2.494
H	-3.716	1.957	-3.719
C	-6.602	-0.990	-0.354
C	-5.144	-0.961	0.085
C	-4.301	-2.007	-0.644
C	-2.803	-1.764	-0.494
O	-2.025	-2.223	-1.370
O	-2.402	-1.078	0.504
H	-7.063	-1.970	-0.172
H	-4.721	0.030	-0.104
H	-5.063	-1.114	1.167
H	-4.531	-3.019	-0.279
H	-4.522	-2.016	-1.718
U	-0.025	-0.894	-0.414
H	-6.694	-0.784	-1.428
H	-7.203	-0.240	0.176
H	-4.631	2.887	-1.562
H	-3.030	2.362	2.876
H	0.768	4.457	1.067
H	4.208	-1.118	-0.237
H	2.030	-4.578	2.597
H	3.479	-3.712	2.082
H	-1.860	-3.169	4.399
H	-0.152	-2.742	4.603

LanM-Np^{3+}

C	-1.067	-2.666	3.899
C	-1.416	-1.182	3.745
C	-0.316	-0.322	3.083
O	0.169	-0.788	1.986
O	-0.005	0.758	3.617
H	-0.824	-3.092	2.922
H	-2.299	-1.104	3.098
H	-1.659	-0.729	4.713
C	2.803	-4.686	1.827
C	2.066	-4.711	0.487
C	1.390	-3.369	0.197
O	0.165	-3.370	-0.130
O	2.079	-2.314	0.296
H	3.347	-5.621	2.012
H	2.781	-4.904	-0.325
H	1.308	-5.499	0.457
C	4.783	-0.648	-0.999
C	5.213	0.739	-0.512
O	6.326	0.925	-0.009
C	3.910	-0.725	-2.278
C	2.410	-0.622	-1.985
O	2.033	0.339	-1.215
O	1.614	-1.473	-2.434
H	5.718	-1.194	-1.154
H	4.192	0.071	-2.980
H	4.078	-1.679	-2.783
N	4.288	1.726	-0.667
C	4.489	3.053	-0.138
C	3.320	3.658	0.647
O	3.399	4.822	1.049
H	3.362	1.426	-1.005
H	5.363	3.021	0.521
H	4.719	3.784	-0.926
N	2.233	2.881	0.860
C	1.078	3.415	1.548
C	-0.133	2.566	1.323
O	-0.163	1.647	0.510
H	2.153	1.942	0.484
H	1.268	3.482	2.628
N	-1.201	2.987	2.027
C	-2.427	2.233	1.977
C	-3.315	2.771	0.836
O	-4.213	3.596	1.034
H	-1.030	3.572	2.830
H	-2.208	1.173	1.834
N	-2.957	2.288	-0.370
C	-3.470	2.765	-1.638
C	-3.248	1.711	-2.739
C	-1.768	1.256	-2.919
O	-1.245	0.726	-1.863
O	-1.230	1.435	-4.014
H	-2.169	1.642	-0.468
H	-2.967	3.703	-1.923
H	-3.848	0.823	-2.502
H	-3.597	2.115	-3.693
C	-6.584	-0.844	-0.404

C	-5.104	-0.867	-0.043
C	-4.315	-1.920	-0.827
C	-2.806	-1.743	-0.667
O	-2.043	-2.069	-1.609
O	-2.387	-1.229	0.427
H	-7.064	-1.813	-0.214
H	-4.665	0.118	-0.230
H	-4.971	-1.047	1.029
H	-4.589	-2.931	-0.493
H	-4.544	-1.874	-1.898
Np	-0.050	-0.874	-0.399
H	-6.728	-0.615	-1.468
H	-7.130	-0.086	0.171
H	-4.534	3.001	-1.522
H	-2.967	2.364	2.916
H	0.889	4.436	1.195
H	4.248	-1.152	-0.184
H	2.101	-4.534	2.654
H	3.513	-3.855	1.843
H	-1.906	-3.231	4.327
H	-0.199	-2.808	4.556

LanM-Pu^{3+}

C	-1.056	-2.693	3.883
C	-1.397	-1.206	3.737
C	-0.301	-0.350	3.061
O	0.184	-0.825	1.969
O	0.006	0.736	3.586
H	-0.821	-3.116	2.903
H	-2.287	-1.118	3.101
H	-1.625	-0.754	4.709
C	2.830	-4.677	1.807
C	2.131	-4.670	0.447
C	1.466	-3.319	0.170
O	0.246	-3.316	-0.188
O	2.148	-2.270	0.312
H	3.370	-5.616	1.986
H	2.867	-4.846	-0.349
H	1.373	-5.457	0.378
C	4.780	-0.615	-1.006
C	5.205	0.767	-0.502
O	6.315	0.952	0.007
C	3.904	-0.677	-2.284
C	2.403	-0.584	-1.989
O	2.018	0.365	-1.207
O	1.614	-1.431	-2.454
H	5.718	-1.154	-1.173
H	4.181	0.130	-2.975
H	4.074	-1.623	-2.803
N	4.277	1.753	-0.650
C	4.471	3.074	-0.105
C	3.292	3.669	0.672
O	3.363	4.830	1.084
H	3.352	1.453	-0.991
H	5.338	3.036	0.564
H	4.710	3.814	-0.882
N	2.205	2.887	0.868
C	1.043	3.411	1.549
C	-0.158	2.549	1.323
O	-0.178	1.628	0.511
H	2.131	1.949	0.486
H	1.229	3.484	2.630
N	-1.232	2.962	2.024
C	-2.453	2.201	1.974
C	-3.342	2.738	0.832
O	-4.238	3.565	1.030
H	-1.073	3.568	2.813
H	-2.228	1.143	1.828
N	-2.988	2.252	-0.374
C	-3.498	2.736	-1.641
C	-3.269	1.689	-2.747
C	-1.787	1.235	-2.929
O	-1.262	0.710	-1.872
O	-1.255	1.409	-4.027
H	-2.203	1.602	-0.473
H	-2.998	3.678	-1.918
H	-3.867	0.798	-2.518
H	-3.617	2.098	-3.701
C	-6.584	-0.901	-0.419
C	-5.115	-0.878	-0.019
C	-4.290	-1.939	-0.752
C	-2.788	-1.718	-0.598
O	-2.019	-2.076	-1.524
O	-2.375	-1.143	0.466
H	-7.048	-1.875	-0.211
H	-4.692	0.109	-0.228
H	-5.006	-1.020	1.062
H	-4.537	-2.944	-0.378
H	-4.514	-1.946	-1.825
Pu	-0.046	-0.870	-0.402
H	-6.704	-0.709	-1.493
H	-7.164	-0.139	0.117
H	-4.563	2.967	-1.527
H	-2.994	2.329	2.913
H	0.844	4.430	1.193
H	4.250	-1.134	-0.197
H	2.106	-4.543	2.618
H	3.539	-3.846	1.861
H	-1.895	-3.255	4.314
H	-0.185	-2.842	4.533

LanM-Am^{3+}

C	-1.070	-2.700	3.875
C	-1.402	-1.209	3.757
C	-0.314	-0.354	3.066
O	0.146	-0.827	1.964
O	0.010	0.726	3.593
H	-0.857	-3.110	2.884
H	-2.306	-1.106	3.143
H	-1.606	-0.767	4.739
C	2.803	-4.701	1.791
C	2.028	-4.706	0.474
C	1.370	-3.350	0.198
O	0.147	-3.336	-0.135
O	2.070	-2.305	0.306
H	3.335	-5.648	1.957
H	2.713	-4.914	-0.359
H	1.253	-5.478	0.463
C	4.781	-0.641	-1.004
C	5.214	0.742	-0.510
O	6.330	0.926	-0.014
C	3.893	-0.708	-2.273
C	2.396	-0.609	-1.968
O	2.025	0.328	-1.165
O	1.593	-1.439	-2.442
H	5.715	-1.184	-1.175
H	4.166	0.094	-2.973
H	4.057	-1.657	-2.790
N	4.287	1.730	-0.650
C	4.488	3.052	-0.107
C	3.314	3.650	0.676
O	3.390	4.814	1.081
H	3.358	1.431	-0.977
H	5.358	3.013	0.557

H	4.723	3.790	-0.887
N	2.229	2.870	0.885
C	1.070	3.400	1.568
C	-0.135	2.542	1.343
O	-0.156	1.617	0.536
H	2.151	1.930	0.510
H	1.257	3.470	2.649
N	-1.209	2.964	2.038
C	-2.434	2.211	1.987
C	-3.321	2.758	0.849
O	-4.208	3.594	1.049
H	-1.051	3.578	2.820
H	-2.214	1.152	1.835
N	-2.972	2.273	-0.359
C	-3.475	2.767	-1.626
C	-3.221	1.735	-2.740
C	-1.733	1.295	-2.898
O	-1.230	0.743	-1.844
O	-1.172	1.506	-3.976
H	-2.193	1.616	-0.459
H	-2.982	3.718	-1.886
H	-3.816	0.837	-2.532
H	-3.554	2.153	-3.695
C	-6.585	-0.854	-0.420
C	-5.107	-0.849	-0.050
C	-4.302	-1.903	-0.816
C	-2.794	-1.701	-0.673
O	-2.037	-2.049	-1.610
O	-2.372	-1.149	0.402
H	-7.053	-1.827	-0.217
H	-4.682	0.139	-0.251
H	-4.978	-1.011	1.025
H	-4.555	-2.913	-0.460
H	-4.538	-1.885	-1.886
Am	-0.047	-0.863	-0.408
H	-6.727	-0.644	-1.488
H	-7.146	-0.096	0.141
H	-4.544	2.985	-1.523
H	-2.972	2.336	2.928
H	0.875	4.419	1.212
H	4.256	-1.153	-0.188
H	2.130	-4.539	2.640
H	3.530	-3.884	1.790
H	-1.905	-3.262	4.315
H	-0.187	-2.865	4.505

LanM-Cm^{3+}

C	-1.024	-2.682	3.908
C	-1.350	-1.194	3.744
C	-0.255	-0.362	3.036
O	0.196	-0.858	1.940
O	0.079	0.724	3.545
H	-0.798	-3.119	2.931
H	-2.250	-1.105	3.122
H	-1.557	-0.724	4.713
C	2.853	-4.661	1.808
C	2.132	-4.664	0.460
C	1.472	-3.311	0.182
O	0.237	-3.301	-0.118
O	2.175	-2.269	0.263
H	3.385	-5.604	1.991
H	2.854	-4.854	-0.346
H	1.367	-5.446	0.414
C	4.760	-0.607	-1.044
C	5.203	0.760	-0.513
O	6.313	0.918	0.003
C	3.859	-0.637	-2.306
C	2.367	-0.530	-1.979
O	2.003	0.419	-1.194
O	1.559	-1.378	-2.421
H	5.692	-1.146	-1.242
H	4.133	0.177	-2.990
H	4.009	-1.578	-2.842
N	4.290	1.762	-0.645
C	4.491	3.063	-0.055
C	3.297	3.657	0.700
O	3.371	4.811	1.134
H	3.360	1.481	-0.985
H	5.335	2.991	0.640
H	4.768	3.821	-0.802
N	2.195	2.887	0.854
C	1.028	3.418	1.522
C	-0.164	2.536	1.326
O	-0.182	1.599	0.534
H	2.116	1.953	0.462
H	1.219	3.527	2.599
N	-1.236	2.953	2.025
C	-2.458	2.195	1.984
C	-3.353	2.730	0.847
O	-4.230	3.578	1.042
H	-1.089	3.603	2.780
H	-2.234	1.137	1.838
N	-3.024	2.222	-0.357
C	-3.538	2.705	-1.624
C	-3.273	1.673	-2.736
C	-1.781	1.249	-2.906
O	-1.263	0.710	-1.854
O	-1.236	1.454	-3.993
H	-2.249	1.560	-0.455
H	-3.060	3.662	-1.888
H	-3.856	0.768	-2.521
H	-3.619	2.084	-3.690
C	-6.597	-0.937	-0.355
C	-5.127	-0.904	0.040
C	-4.287	-1.912	-0.746
C	-2.788	-1.670	-0.594
O	-2.011	-2.082	-1.493
O	-2.388	-1.030	0.434
H	-7.041	-1.929	-0.190
H	-4.723	0.099	-0.126
H	-5.011	-1.093	1.113
H	-4.509	-2.940	-0.424
H	-4.519	-1.872	-1.817
Cm	-0.045	-0.867	-0.424
H	-6.726	-0.699	-1.418
H	-7.189	-0.212	0.218
H	-4.610	2.909	-1.519
H	-2.993	2.325	2.927
H	0.812	4.423	1.137
H	4.244	-1.143	-0.238
H	2.143	-4.509	2.629
H	3.572	-3.838	1.840
H	-1.865	-3.231	4.352
H	-0.149	-2.831	4.554

LanM-Bk^{3+}

C	-1.038	-2.717	3.879
C	-1.368	-1.229	3.714
C	-0.267	-0.388	3.025
O	0.180	-0.860	1.917
O	0.077	0.682	3.560
H	-0.802	-3.153	2.904
H	-2.260	-1.143	3.080
H	-1.591	-0.764	4.681

C	2.826	-4.702	1.763
C	1.976	-4.715	0.493
C	1.390	-3.331	0.198
O	0.155	-3.266	-0.103
O	2.144	-2.327	0.259
H	3.313	-5.670	1.941
H	2.598	-5.000	-0.367
H	1.157	-5.437	0.560
C	4.769	-0.631	-1.041
C	5.218	0.739	-0.525
O	6.336	0.903	-0.029
C	3.847	-0.671	-2.287
C	2.359	-0.557	-1.945
O	2.009	0.376	-1.135
O	1.536	-1.381	-2.405
H	5.698	-1.171	-1.251
H	4.112	0.135	-2.984
H	3.986	-1.616	-2.818
N	4.300	1.738	-0.645
C	4.505	3.041	-0.062
C	3.322	3.631	0.715
O	3.405	4.783	1.153
H	3.364	1.451	-0.964
H	5.362	2.976	0.616
H	4.763	3.798	-0.816
N	2.223	2.862	0.882
C	1.065	3.393	1.566
C	-0.138	2.530	1.356
O	-0.166	1.605	0.550
H	2.134	1.930	0.486
H	1.260	3.477	2.644
N	-1.208	2.947	2.061
C	-2.432	2.192	2.009
C	-3.324	2.744	0.877
O	-4.198	3.592	1.081
H	-1.052	3.570	2.837
H	-2.210	1.136	1.849
N	-2.995	2.248	-0.333
C	-3.502	2.751	-1.595
C	-3.210	1.744	-2.724
C	-1.711	1.333	-2.874
O	-1.219	0.750	-1.832
O	-1.139	1.588	-3.935
H	-2.221	1.587	-0.435
H	-3.034	3.720	-1.834
H	-3.791	0.832	-2.540
H	-3.539	2.174	-3.675
C	-6.592	-0.881	-0.371
C	-5.114	-0.863	-0.005
C	-4.291	-1.863	-0.820
C	-2.789	-1.641	-0.671
O	-2.018	-2.024	-1.586
O	-2.379	-1.042	0.380
H	-7.038	-1.873	-0.212
H	-4.708	0.141	-0.161
H	-4.979	-1.070	1.062
H	-4.523	-2.894	-0.517
H	-4.530	-1.798	-1.888
Bk	-0.061	-0.862	-0.427
H	-6.740	-0.627	-1.429
H	-7.167	-0.161	0.223
H	-4.578	2.938	-1.500
H	-2.969	2.311	2.952
H	0.861	4.408	1.202
H	4.267	-1.163	-0.223
H	2.213	-4.466	2.639
H	3.594	-3.929	1.684
H	-1.882	-3.269	4.314
H	-0.170	-2.864	4.533

LanM-Cf^{3+}

C	-1.051	-2.770	3.824
C	-1.375	-1.278	3.685
C	-0.269	-0.429	3.012
O	0.180	-0.882	1.898
O	0.077	0.630	3.569
H	-0.818	-3.190	2.841
H	-2.266	-1.178	3.051
H	-1.598	-0.829	4.659
C	2.804	-4.746	1.683
C	1.866	-4.740	0.477
C	1.312	-3.340	0.192
O	0.076	-3.244	-0.094
O	2.091	-2.355	0.236
H	3.265	-5.731	1.839
H	2.411	-5.065	-0.420
H	1.026	-5.427	0.612
C	4.770	-0.647	-1.064
C	5.232	0.713	-0.532
O	6.356	0.861	-0.043
C	3.823	-0.665	-2.293
C	2.342	-0.544	-1.925
O	2.012	0.374	-1.090
O	1.502	-1.348	-2.390
H	5.692	-1.187	-1.299
H	4.082	0.148	-2.985
H	3.946	-1.605	-2.837
N	4.319	1.718	-0.628
C	4.532	3.009	-0.017
C	3.348	3.594	0.760
O	3.435	4.739	1.214
H	3.379	1.441	-0.937
H	5.383	2.923	0.667
H	4.804	3.778	-0.755
N	2.242	2.829	0.911
C	1.084	3.360	1.595
C	-0.122	2.503	1.377
O	-0.154	1.586	0.563
H	2.150	1.901	0.506
H	1.277	3.437	2.674
N	-1.190	2.918	2.085
C	-2.419	2.171	2.020
C	-3.306	2.746	0.896
O	-4.172	3.601	1.110
H	-1.033	3.531	2.868
H	-2.204	1.116	1.845
N	-2.982	2.262	-0.320
C	-3.484	2.785	-1.576
C	-3.163	1.801	-2.720
C	-1.658	1.402	-2.848
O	-1.192	0.768	-1.825
O	-1.057	1.715	-3.878
H	-2.217	1.591	-0.426
H	-3.026	3.764	-1.791
H	-3.741	0.881	-2.565
H	-3.476	2.248	-3.668
C	-6.593	-0.849	-0.404
C	-5.115	-0.817	-0.035
C	-4.287	-1.829	-0.829
C	-2.784	-1.599	-0.685
O	-2.015	-1.978	-1.602
O	-2.373	-0.998	0.365
H	-7.035	-1.838	-0.227

H	-4.713	0.186	-0.209
H	-4.981	-1.003	1.036
H	-4.514	-2.855	-0.505
H	-4.525	-1.786	-1.898
Cf	-0.067	-0.847	-0.431
H	-6.740	-0.615	-1.466
H	-7.172	-0.120	0.176
H	-4.564	2.954	-1.491
H	-2.955	2.279	2.964
H	0.885	4.378	1.237
H	4.281	-1.190	-0.245
H	2.265	-4.476	2.598
H	3.594	-4.004	1.540
H	-1.897	-3.326	4.251
H	-0.183	-2.932	4.475

LanM-Es^{3+}

C	-1.036	-2.799	3.822
C	-1.363	-1.307	3.699
C	-0.269	-0.456	3.010
O	0.164	-0.916	1.892
O	0.083	0.604	3.558
H	-0.813	-3.209	2.833
H	-2.266	-1.201	3.084
H	-1.567	-0.864	4.681
C	2.842	-4.732	1.682
C	1.966	-4.694	0.431
C	1.410	-3.290	0.173
O	0.181	-3.198	-0.147
O	2.177	-2.302	0.275
H	3.310	-5.715	1.826
H	2.561	-4.978	-0.449
H	1.130	-5.397	0.499
C	4.772	-0.608	-1.052
C	5.212	0.761	-0.526
O	6.334	0.930	-0.040
C	3.828	-0.645	-2.282
C	2.343	-0.546	-1.919
O	1.993	0.381	-1.102
O	1.520	-1.372	-2.374
H	5.703	-1.134	-1.284
H	4.075	0.168	-2.977
H	3.966	-1.586	-2.822
N	4.284	1.753	-0.626
C	4.482	3.052	-0.029
C	3.297	3.625	0.756
O	3.370	4.775	1.200
H	3.348	1.463	-0.939
H	5.342	2.985	0.646
H	4.733	3.818	-0.777
N	2.207	2.843	0.923
C	1.044	3.357	1.611
C	-0.150	2.483	1.392
O	-0.164	1.559	0.585
H	2.127	1.912	0.523
H	1.236	3.432	2.691
N	-1.225	2.887	2.093
C	-2.448	2.134	2.028
C	-3.340	2.702	0.903
O	-4.214	3.548	1.119
H	-1.092	3.544	2.844
H	-2.224	1.080	1.851
N	-3.008	2.226	-0.313
C	-3.514	2.745	-1.569
C	-3.189	1.767	-2.714
C	-1.679	1.393	-2.852
O	-1.192	0.779	-1.826
O	-1.094	1.704	-3.892
H	-2.236	1.562	-0.422
H	-3.064	3.728	-1.783
H	-3.753	0.839	-2.554
H	-3.517	2.209	-3.660
C	-6.589	-0.921	-0.410
C	-5.113	-0.868	-0.035
C	-4.261	-1.841	-0.851
C	-2.763	-1.594	-0.688
O	-1.975	-1.983	-1.586
O	-2.369	-0.973	0.356
H	-7.012	-1.923	-0.259
H	-4.731	0.146	-0.182
H	-4.980	-1.079	1.032
H	-4.474	-2.881	-0.564
H	-4.490	-1.770	-1.921
Es	-0.060	-0.832	-0.427
H	-6.738	-0.663	-1.466
H	-7.185	-0.219	0.186
H	-4.595	2.908	-1.481
H	-2.986	2.236	2.972
H	0.831	4.373	1.257
H	4.290	-1.155	-0.232
H	2.252	-4.504	2.577
H	3.625	-3.974	1.607
H	-1.875	-3.361	4.254
H	-0.159	-2.965	4.460

LanM-Fm^{3+}

C	-0.961	-2.715	3.933
C	-1.338	-1.264	3.598
C	-0.215	-0.427	2.938
O	0.243	-0.872	1.822
O	0.146	0.621	3.504
H	-0.628	-3.231	3.027
H	-2.174	-1.277	2.888
H	-1.658	-0.734	4.502
C	2.891	-4.686	1.782
C	1.946	-4.680	0.584
C	1.583	-3.240	0.197
O	0.372	-3.050	-0.173
O	2.464	-2.364	0.266
H	3.242	-5.698	2.028
H	2.433	-5.148	-0.284
H	1.029	-5.244	0.780
C	4.771	-0.620	-1.071
C	5.232	0.741	-0.546
O	6.357	0.897	-0.061
C	3.797	-0.643	-2.276
C	2.323	-0.514	-1.886
O	1.992	0.419	-1.070
O	1.480	-1.324	-2.338
H	5.693	-1.148	-1.334
H	4.044	0.162	-2.982
H	3.904	-1.588	-2.813
N	4.317	1.746	-0.642
C	4.528	3.034	-0.028
C	3.334	3.623	0.731
O	3.411	4.774	1.172
H	3.373	1.465	-0.939
H	5.369	2.945	0.668
H	4.813	3.804	-0.760
N	2.231	2.856	0.883
C	1.070	3.395	1.555
C	-0.137	2.536	1.350
O	-0.192	1.640	0.514
H	2.144	1.921	0.490

H	1.263	3.496	2.633
N	-1.190	2.933	2.096
C	-2.412	2.173	2.049
C	-3.316	2.714	0.922
O	-4.203	3.549	1.132
H	-1.004	3.500	2.907
H	-2.193	1.116	1.893
N	-2.992	2.220	-0.289
C	-3.530	2.701	-1.545
C	-3.313	1.665	-2.663
C	-1.830	1.247	-2.903
O	-1.258	0.702	-1.882
O	-1.338	1.457	-4.014
H	-2.212	1.569	-0.390
H	-3.047	3.651	-1.827
H	-3.885	0.760	-2.419
H	-3.703	2.072	-3.601
C	-6.579	-0.945	-0.260
C	-5.112	-0.912	0.147
C	-4.263	-1.895	-0.660
C	-2.769	-1.618	-0.543
O	-1.991	-2.072	-1.421
O	-2.361	-0.904	0.430
H	-7.018	-1.943	-0.127
H	-4.713	0.096	0.008
H	-5.005	-1.127	1.216
H	-4.452	-2.932	-0.347
H	-4.515	-1.852	-1.727
No	-0.105	-0.803	-0.471
H	-6.700	-0.678	-1.318
H	-7.180	-0.240	0.326
H	-4.596	2.920	-1.411
H	-2.945	2.297	2.993
H	0.868	4.406	1.178
H	4.309	-1.174	-0.245
H	2.395	-4.277	2.669
H	3.752	-4.050	1.570
H	-1.816	-3.263	4.350
H	-0.145	-2.758	4.665

LanM-Md^{3+}

C	-1.041	-2.804	3.821
C	-1.408	-1.327	3.619
C	-0.294	-0.459	2.987
O	0.167	-0.875	1.861
O	0.054	0.578	3.579
H	-0.750	-3.246	2.864
H	-2.265	-1.276	2.937
H	-1.693	-0.864	4.570
C	2.829	-4.750	1.680
C	1.863	-4.710	0.499
C	1.415	-3.277	0.193
O	0.200	-3.122	-0.171
O	2.241	-2.345	0.304
H	3.230	-5.758	1.851
H	2.355	-5.101	-0.404
H	0.977	-5.329	0.673
C	4.786	-0.626	-1.036
C	5.225	0.752	-0.534
O	6.353	0.936	-0.067
C	3.826	-0.682	-2.252
C	2.341	-0.577	-1.887
O	1.993	0.336	-1.050
O	1.512	-1.378	-2.369
H	5.716	-1.150	-1.275
H	4.066	0.119	-2.965
H	3.957	-1.630	-2.779
N	4.288	1.735	-0.633
C	4.487	3.044	-0.058
C	3.315	3.617	0.746
O	3.392	4.769	1.184
H	3.349	1.435	-0.926
H	5.362	2.994	0.598
H	4.713	3.802	-0.821
N	2.229	2.832	0.936
C	1.076	3.349	1.637
C	-0.134	2.503	1.398
O	-0.168	1.608	0.561
H	2.145	1.901	0.538
H	1.269	3.397	2.718
N	-1.201	2.899	2.121
C	-2.423	2.143	2.044
C	-3.311	2.719	0.920
O	-4.185	3.564	1.137
H	-1.038	3.479	2.927
H	-2.202	1.090	1.862
N	-2.979	2.246	-0.298
C	-3.480	2.775	-1.552
C	-3.175	1.795	-2.702
C	-1.672	1.396	-2.847
O	-1.192	0.774	-1.824
O	-1.085	1.699	-3.888
H	-2.204	1.588	-0.409
H	-3.015	3.750	-1.766
H	-3.753	0.876	-2.544
H	-3.499	2.246	-3.645
C	-6.577	-0.879	-0.412
C	-5.097	-0.838	-0.048
C	-4.260	-1.835	-0.854
C	-2.758	-1.604	-0.703
O	-1.981	-1.970	-1.617
O	-2.346	-1.015	0.356
H	-7.011	-1.874	-0.241
H	-4.705	0.170	-0.214
H	-4.959	-1.033	1.021
H	-4.484	-2.867	-0.546
H	-4.494	-1.778	-1.923
Md	-0.079	-0.818	-0.426
H	-6.731	-0.637	-1.471
H	-7.159	-0.160	0.178
H	-4.558	2.954	-1.461
H	-2.966	2.242	2.986
H	0.883	4.377	1.306
H	4.317	-1.164	-0.203
H	2.333	-4.423	2.600
H	3.659	-4.063	1.498
H	-1.887	-3.374	4.226
H	-0.199	-2.916	4.516

LanM-No^{3+}

C	-0.961	-2.715	3.933
C	-1.338	-1.264	3.598
C	-0.215	-0.427	2.938
O	0.243	-0.872	1.822
O	0.146	0.621	3.504
H	-0.628	-3.231	3.027
H	-2.174	-1.277	2.888
H	-1.658	-0.734	4.502
C	2.891	-4.686	1.782
C	1.946	-4.680	0.584
C	1.583	-3.240	0.197
O	0.372	-3.050	-0.173
O	2.464	-2.364	0.266
H	3.242	-5.698	2.028

H	2.433	-5.148	-0.284
H	1.029	-5.244	0.780
C	4.771	-0.620	-1.071
C	5.232	0.741	-0.546
O	6.357	0.897	-0.061
C	3.797	-0.643	-2.276
C	2.323	-0.514	-1.886
O	1.992	0.419	-1.070
O	1.480	-1.324	-2.338
H	5.693	-1.148	-1.334
H	4.044	0.162	-2.982
H	3.904	-1.588	-2.813
N	4.317	1.746	-0.642
C	4.528	3.034	-0.028
C	3.334	3.623	0.731
O	3.411	4.774	1.172
H	3.373	1.465	-0.939
H	5.369	2.945	0.668
H	4.813	3.804	-0.760
N	2.231	2.856	0.883
C	1.070	3.395	1.555
C	-0.137	2.536	1.350
O	-0.192	1.640	0.514
H	2.144	1.921	0.490
H	1.263	3.496	2.633
N	-1.190	2.933	2.096
C	-2.412	2.173	2.049
C	-3.316	2.714	0.922
O	-4.203	3.549	1.132
H	-1.004	3.500	2.907
H	-2.193	1.116	1.893
N	-2.992	2.220	-0.289
C	-3.530	2.701	-1.545
C	-3.313	1.665	-2.663
C	-1.830	1.247	-2.903
O	-1.258	0.702	-1.882
O	-1.338	1.457	-4.014
H	-2.212	1.569	-0.390
H	-3.047	3.651	-1.827
H	-3.885	0.760	-2.419
H	-3.703	2.072	-3.601
C	-6.579	-0.945	-0.260
C	-5.112	-0.912	0.147
C	-4.263	-1.895	-0.660
C	-2.769	-1.618	-0.543
O	-1.991	-2.072	-1.421
O	-2.361	-0.904	0.430
H	-7.018	-1.943	-0.127
H	-4.713	0.096	0.008
H	-5.005	-1.127	1.216
H	-4.452	-2.932	-0.347
H	-4.515	-1.852	-1.727
No	-0.105	-0.803	-0.471
H	-6.700	-0.678	-1.318
H	-7.180	-0.240	0.326
H	-4.596	2.920	-1.411
H	-2.945	2.297	2.993
H	0.868	4.406	1.178
H	4.309	-1.174	-0.245
H	2.395	-4.277	2.669
H	3.752	-4.050	1.570
H	-1.816	-3.263	4.350
H	-0.145	-2.758	4.665

LanM-Lr^{3+}

C	-0.980	-2.899	3.773
C	-0.230	-1.614	4.149
C	0.239	-0.699	2.981
O	0.025	-1.147	1.798
O	0.782	0.371	3.297
H	-1.893	-2.669	3.208
H	-0.845	-0.977	4.809
H	0.670	-1.853	4.739
C	2.980	-4.605	1.594
C	2.028	-4.744	0.403
C	1.996	-3.405	-0.359
O	0.863	-2.811	-0.370
O	3.070	-3.010	-0.844
H	3.094	-5.545	2.151
H	2.398	-5.526	-0.274
H	1.020	-5.017	0.728
C	4.687	-0.353	-1.093
C	5.077	1.024	-0.559
O	6.216	1.272	-0.151
C	3.697	-0.408	-2.273
C	2.237	-0.385	-1.836
O	1.968	0.193	-0.707
O	1.343	-0.914	-2.524
H	5.633	-0.824	-1.378
H	3.869	0.418	-2.976
H	3.833	-1.342	-2.819
N	4.081	1.956	-0.561
C	4.238	3.254	0.049
C	2.999	3.787	0.771
O	2.945	4.978	1.091
H	3.148	1.605	-0.777
H	5.071	3.201	0.759
H	4.504	4.031	-0.681
N	1.999	2.914	1.033
C	0.775	3.383	1.636
C	-0.339	2.386	1.469
O	-0.316	1.469	0.656
H	2.041	1.931	0.781
H	0.915	3.588	2.706
N	-1.394	2.649	2.276
C	-2.649	1.979	2.049
C	-3.394	2.674	0.893
O	-3.830	3.823	1.028
H	-1.433	3.559	2.712
H	-2.454	0.924	1.845
N	-3.438	1.965	-0.253
C	-3.760	2.582	-1.535
C	-3.127	1.800	-2.694
C	-1.593	1.669	-2.486
O	-1.242	0.565	-1.930
O	-0.882	2.623	-2.813
H	-2.981	1.050	-0.263
H	-3.374	3.607	-1.525
H	-3.584	0.807	-2.772
H	-3.323	2.350	-3.621
C	-6.640	-1.250	-0.413
C	-5.239	-1.447	0.153
C	-4.257	-1.980	-0.892
C	-2.792	-1.690	-0.580
O	-1.902	-2.299	-1.228
O	-2.517	-0.784	0.273
H	-7.058	-2.185	-0.810
H	-4.856	-0.494	0.523
H	-5.268	-2.123	1.017
H	-4.374	-3.056	-1.067
H	-4.448	-1.497	-1.861
Lr	-0.141	-0.785	-0.457
H	-6.629	-0.524	-1.235

H	-7.338	-0.876	0.347	H	0.484	4.331	1.164	H	3.963	-4.286	1.236
H	-4.851	2.652	-1.662	H	4.272	-0.954	-0.278	H	-1.259	-3.472	4.670
H	-3.258	2.063	2.955	H	2.614	-3.843	2.291	H	-0.364	-3.538	3.127
